# The skull of the gerrhonotine lizard *Elgaria panamintina* (Squamata: Anguidae)

**DOI:** 10.1371/journal.pone.0199584

**Published:** 2018-06-28

**Authors:** David T. Ledesma, Simon G. Scarpetta

**Affiliations:** Jackson School of Geosciences, The University of Texas, Austin, Texas, United States of America; The Mammoth Site, UNITED STATES

## Abstract

We provide the first description of the skull, osteoderms, and hyoid apparatus of the poorly known alligator lizard *Elgaria panamintina*, and compare the cranial osteology of that species to the widespread and well-studied taxon *Elgaria multicarinata*. Patterns of morphological variation resulting from ontogenetic transformations and pathology are discussed. We employed x-ray computed tomography (CT) scans to examine two adult specimens of *Elgaria panamintina* and two adult specimens of *Elgaria multicarinata*, in addition to examining multiple traditionally prepared skeletal specimens of the latter species. CT scans provide simultaneous study of both articulated and disarticulated elements, allowing us to describe and document the morphology of the skull with exceptional precision and detail. The description of the skull of *Elgaria panamintina* serves as a generalization for all *Elgaria*; here we provide the first complete description of the skull of this genus for future uses in morphological and phylogenetic studies of both extant species and fossils.

## Introduction

Lizards of the anguid clade Gerrhonotinae have been the subject of previous phylogenetic [1–6], paleontological [5, 7, 8], ecological [9], and biogeographic studies [10]. Recent authors variably grouped the Gerrhonotinae in a clade with Diploglossinae [11], Anguinae [2, 12], or as a sister group to a monophyletic Anguinae + Diploglossinae [13] among extant anguids. There are six genera of extant gerrhonotine lizards, *Abronia*, *Barisia*, *Coloptychon*, *Elgaria*, *Gerrhonotus*, and *Mesaspis* [14]. There are over 50 species of extant gerrhonotines distributed across the western and central United States, Mexico, and Central America [5]. Despite their relative popularity in the literature, important aspects of gerrhonotine biology, such as taxonomy, intra-clade phylogenetic relationships, and cranial osteology are still poorly understood.

Classification of Gerrhonotinae dates to Wiegmann (1828) [15] with the description of six species assigned to the genus *Gerrhonotus*. Five of those species originally assigned to *Gerrhonotus* have since been re-assigned to other genera, including *Elgaria coerulea*, *Abronia taeniatus*, *Abronia deppi*, *Barisia rudicollis*, and *Barisia imbricata*. Generic assignation varied occasionally between *Gerrhonotus* and *Elgaria* [16, 17] and *Gerrhonotus* and *Barisia* [16]. Other gerrhonotine species identified and described during the 19^th^ century include *Elgaria multicarinata* (*Gerrhonotus multicarinatus*, Blainville 1835 [18]), and *E*. *kingii* (*Gerrhonotus kingii*, Gray 1838 [19]). The genera *Elgaria*, *Barisia*, and *Abronia* were described by Gray in 1838 [19], and the genus *Mesaspis* was described by Cope in 1877 [20].

Taxonomic controversy regarding the recognition of gerrhonotine genera began in the late 1940’s, and has continued through the present day. The genera *Elgaria*, *Gerrhonotus*, *Barisia*, *Abronia* and *Coloptychon* were recognized by Tihen in 1949 [21, 22]. Only *Gerrhonotus* (subgenera *Gerrhonotus* and *Barisia*), *Abronia*, and *Coloptychon* were accepted by Stebbins in 1958 [23]. A less inclusive “gerrhonotiform” lizard group including *Elgaria*, *Gerrhonotus*, and *Barisia*, but excluding *Abronia* and *Coloptychon*, was recognized by Criley [24]. *Elgaria*, *Gerrhonotus*, and *Barisia* (specifically refuting Stebbins [23]), as well as *Abronia* and *Coloptychon*, were accepted by Waddick & Smith in 1974 [25]. All genera excluding *Mesaspis* were accepted in 1982 by Gauthier [26]. However, Gauthier [26] also pointed to the likely paraphyly of *Barisia* (as containing both *Barisia* and *Mesaspis*), and considered some *Barisia* more closely related to *Abronia*. All six currently recognized genera were accepted and discussed in 1987 by Good [3]. The paraphyly of *Barisia* was again recovered by Good [3]. Recent molecular studies [6] recovered paraphyletic *Abronia* and *Mespasis*.

Studies focused specifically on the cranial osteology of gerrhonotine lizards were relatively limited in scope and included only a few species, usually using exemplars from commonly available taxa like *Abronia deppii*, *Abronia oaxacae*, *Barisia imbricata*, *Elgaria multicarinata*, *Elgaria coerulea*, *Elgaria kingii*, *Gerrhonotus infernalis*, and *Gerrhonotus liocephalus*. The most comprehensive study to date of gerrhonotine cranial osteology, which was conducted in a phylogenetic context, included 13 species from 5 genera [3]. The cranial anatomy of gerrhonotine lizards independent of phylogenetic context was described by Criley [24], who included descriptions of the general form and structure of each cranial element and comparisons between different genera within Gerrhonotinae as well as to other lizard clades. *Elgaria*, *Gerronotus*, and *Barisia* were indistinguishable from each other to Criley [24] on the basis of osteocranial characters. Other authors [1, 2, 11, 26–28] included gerrhonotine cranial osteology as part of their higher-level phylogenetic analyses of Anguimorpha or Squamata. A large assemblage of fossil anguids and xenosaurids from the Eocene of Wyoming included specimens referred to Gerrhonotinae [26], and a comprehensive phylogenetic analysis of that assemblage and other extant and extinct Anguioidea produced a detailed description of the relationships and apomorphies of a sample of gerrhonotine lizard taxa. A substantial number of other fossils were assigned to Gerrhonotinae by numerous authors [8, 29–39]. In an apomorphy-based reanalysis of most of those specimens, only 3 of 166 specimens examined met an apomorphic criterion for identification to the Gerrhonotinae [5].

The osteology of most species of gerrhonotine lizard remains unexamined, and no comprehensive description of any one genus or species exists. Here, we describe in detail the skull of the largely unstudied species *Elgaria panamintina*, and in the process, provide a baseline description of the cranial osteology of the genus *Elgaria* for future morphological and phylogenetic analyses. As far as we know, only one skeletal specimen of *Elgaria panamintina* currently exists, and only a handful of wet specimens are accessioned in museum collections. In light of this paucity of material, we use x-ray computed tomography (CT) scans of multiple alcohol-preserved specimens to document the skull, ontogenetic variation, bone pathology, and intraspecific and intraspecimen variation of *Elgaria panamintina* for the first time.

*Elgaria panamintina* is a relatively large, robust anguid like most other gerrhonotines. The species is secretive, generally occupies mesic areas, and is a good climber [23, 40]. It has been found across a relatively limited elevational range from 3800 to 5100 ft. (1158–1554 m), although some specimens have been found near the top of mountains at 6000 ft. (1829 m) [23, 40]. It lives in the Panamint Mountains of California, where acceptable temperature and humidity conditions exist above the extremely arid Mojave Desert. A strong connection with open water was noted for *Elgaria panamintina* by Stebbins [23], but later observers found the lizard in arid localities in the mountains well away from water [40]. *Elgaria panamintina* is generally considered to be the sister taxon to the well-known species *Elgaria multicarinata* in phylogenetic analyses of molecular data [6, 17, 41, 42]. Although recent work called into question the monophyly of *Elgaria multicarinata* [42], a close relationship between *Elgaria panamintina* and some subset of the *Elgaria multicarinata* complex likely still exists. Because of this close relationship we also provide a comparison of the cranial osteology of *Elgaria panamintina* and *Elgaria multicarinata*.

## Methods

We examined two specimens of *Elgaria panamintina* loaned from the University of California at Berkeley Museum of Vertebrate Zoology (MVZ), MVZ 75918 and MVZ 191076. CT scans permit exceptional analysis of each individual element, as well as the articulation and positional relationships between different bones. We also examined several traditionally prepared articulated and disarticulated specimens of the closely related species *Elgaria multicarinata* as well as two CT data sets of *Elgaria multicarinata*.

Both specimens of *Elgaria panamintina* are adult males from the Panamint Mountains in Inyo County, California. The specimen MVZ 75918 is from Daisy Canyon, and was collected on June 11, 1959. The other specimen MVZ 191076 was collected from Grapevine Canyon, June 1991. Both CT-scanned *Elgaria multicarinata* (Texas Natural History Collection, TNHC 4478 and TNHC 35666) are adults from Los Angeles, California.

All specimens were scanned at The University of Texas at Austin High-Resolution X-ray Computed Tomography (CT) Facility (UTCT) on a high-resolution NSI scanner with a Fein Focus high power source. The raw data set consists of 1774 CT slices for MVZ 75918, 1771 slices for MVZ 191076, and 1735 slices for both TNHC 4478 and TNHC 35666. The voxel size for both MVZ 75918 and MVZ 191076 is 0.0181 mm, and for both TNHC 4478 and TNHC 35666 it is 0.0186 mm. The raw data were resliced in order to provide three slice planes for digital processing. We digitally disarticulated the skulls by segmenting CT slices into three-dimensional models of each cranial element using the Avizo 3D 8.1 software. The segmented cranial elements were then examined in articulation with each other and as disarticulated isolated elements. Some elements were largely fused to each other (i.e., the surangular and articular, some parts of the braincase); in those cases, we left the entire element intact rather than using sutures or approximating the boundaries between different elements to produce separate bones. In our descriptions and comparisons, we follow the anatomical terminology of Evans [43] unless otherwise noted. See Table 1 for abbreviations of anatomical terms. Some figures contain a reference skull or skulls that highlight relevant anatomy; references skulls are located at the top or top left of the figure. Arrows in the figures face anteriorly.

**Table 1:** Anatomical abbreviations.

Abbreviation	Anatomical structure	Abbreviation	Anatomical structure
1c	first ceratobranchial	N.ft	nasal facets
Aar	anterior ampullar recess	no.lhv	notch for the lateral head vein
ac.r	acoustic recess	nu	nuchal
add.fs	adductor fossa	nv.f	neurovascular foramen
a.i.a.f	anterior inferior alveolar foramen	occ	osseous common crus
aip	anterior inferior process	o.pr	orbital process
al.pr	anterolateral process	Os	orbitosphenoid
al.Px.pr	anterolateral lappet of premaxillary process	Ot	otooccipital
alv.p	alveolar plate	P	parietal
am.Co.pr	amteromedial process of the coronoid	p.o	parietal osteoderm
a.m.f	anterior mylohyoid foramen	Pa	palatine
am.pr	anteromedial process	Pal	palpebral
An	angular	Pa.Mx.pr	maxillary process of palatine
An.pr	angular process	Pa.pl	palatal plate
ant.f	anterolateral foramen	pa.pr	palatal process
Art	articular	Par	posterior ampullar recess
art.s	articular surface for quadrate	Pa.V.pr	vomerine process of the palatine
a.San.f	anterior surangular foramen	pd.c	canal for the perilymphatic duct
asc	anterior semicircular canal	p.d.pr	posterodorsal process
a.Spl.pr	anterior process of the splenial	p.Ep.g	postepipterygoid groove
a.vc	anterior vidian canal opening	Per.f	perilymphatic foramen
bac.f	basicranial fenestra	P.f	parietal foramen
bh	basihyal	Pfr	postfrontal
Bo	basioccipital	Pfr.ft	postfrontal facet
Bo.co	basioccipital condyle	Pfr.p.pr	postfrontal posterior process
b.tb	basal tubercle	Pfr.s	postfrontal spur
Bt.pr	basipterygoid process	P.ft	parietal facet
cch	conch	pit.f	pituitary fossa
c.co	central column of quadrate	pl	processus lingualis
ce.co	cephalic condyle	pm.pr	posteromedial process
ch	choana	Po	postorbital
ch.g	choanal groove	poc.pr	paroccipital process
Co	coronoid	Po.pr	postorbital process
Co.pr	coronoid process	Ppas	pit for the processus ascendens
cn.ts	ossified connective tissue forming vidian bridge	pp.pr	postparietal process
cr.cr	crista cranii	p.Pt.pr	posterior pterygoid process
cr.if	crista interfenestralis	pr.as	processus ascendens
cr.Pro	crista prootica	pre.Art.pr	prearticular process
cr.s	crista sellaris	Prf	prefrontal
cr.tb	crista tuberalis	Prf.ft	prefrontal facet
cr.tv	crista transversalis	Prf.m.b	prefrontal main body
cv.ca	cavum capularis	Pro	prootic
D	dentary	Pro.a.pr	Prootic alar process
d.s	dorsum sella	Pro.p.pr	posterior process of the prootic
Ec	ectopterygoid	p.San.f	posterior surangular foramen
Ec.s	lateral ectopterygoid spur	psc	posterior semicircular canal
Ep	epipterygoid	Psp.pr	parasphenoid process
Ep.pr	epipterygoid process	Pt	pterygoid
f.6	abducent foramen	Pt.fl	pterygoid flange
f.7	foramen for facial nerve	Pt.ft	pterygoid facet
f.8	foramen for vestibulocochlear nerve	Pt.lm	lower medial pterygoid lamina
f.9	foramen for glossopharyngeal nerve	p.vc	posterior vidian canal opening
f.12	foramina for hypoglossal nerve	pv.pr.Prf	posteroventral process of the prefrontal
f.ch.ty	foramen for chorda tympani branch of facial nerve	p.v.pr.Spl	posteroventral process of the splenial
f.co	fossa columella	Px	premaxilla
fe	free epibranchial	Px.a.f	anterior premaxillary foramen
f.end	endolymphatic foramen	Px.n.pr	nasal process
f.mg	foramen magnum	Px.pr	premaxillary process
f.m.Pa.n	foramen for the medial palatine nerve	Q	quadrate
f.Mx.5	maxillary trigeminal foramina	Q.pr	quadrate process
f.o	fenestra ovalis	rapr	retroarticular process
f.Op.5	foramen for ophthalmic branch of cranial nerve 5	r.v.j	recessus vena jugularis
Fr	frontal	sac	superior alveolar canal
fr.o	frontal osteoderm	San	surangular
fr.p	frontoparietal	San.cr	surangular crest
Fr.pr	frontal process	San.pr	surangular process
H	hyoid apparatus	scl	scleral ossicles
hc	hyoid cornu	sd.sh	subdental shelf
hsc	horizontal semicircular canal	s.l	supralabial
icf	internal carotid foramen	So	supraoccipital
i.l	infralabial	s.oc	supraocular
ims	intramandibular septum	So.cr	supraoccipital crest
in.pr	incisive process	sof	suborbital fenestra
i.o.f	infraorbital foramen	Sp	sphenoid
i.p	interparietal	Sp.a.pr	sphenoid alar process
i.pro	incisura prootica	Spl	splenial
i.Pt.v	interpterygoid vacuity	spr.cl	supracilliary
J	jugal	Sp.w	sphenoid wing
J.pr	jugal process	Sq	squamosal
J.s	jugal spur	Sq.pr	squamosal process
L	lacrimal	Sq.so	squamosal socket
la.pr	lateral process	St	supratemporal
L.d.f	lacrimal dorsal flange	s.t	sella turcica
L.f	lacrimal foramen	stat.m	statolithic mass
lg.r	lagenar recess	stf	supratemporal fenestra
lrst	lateral aperature for the recessus scali tympani	Stp	stapes
ma.co	mandibular condyle	st.pr	supratrigeminal process
m.g	medial groove	Sx	septomaxilla
Mk.fs	meckelian fossa	Sx.p.pr	posterior process of septomaxilla
m.r	medial recess for nasal sac	Sx.pr	anterolateral projection of septomaxilla
mrst	medial aperature for the recessus scali tympani	sy	symphysis
Mx	maxilla	tr	trabecula
Mx.f.pr	facial process	ty.cr	tympanic crest
Mx.lp	maxillary lappet	u.r	utricular recess
Mx.p.o.pr	posterior orbital process of maxilla	V	vomer
Mx.pr	maxillary process	v.f	vagus foramen
Mx.s	maxilla spur	vno	vomeronasal opening
Mx.sh	maxillary shelf	V.p.Pa.pr	posterior palatine process of vomer
N	nasal	V.re	central recess of the vomer
n.f	nutrient foramina		

## Description

### General features of the skull of *Elgaria panamintina*

**Fig 1:**
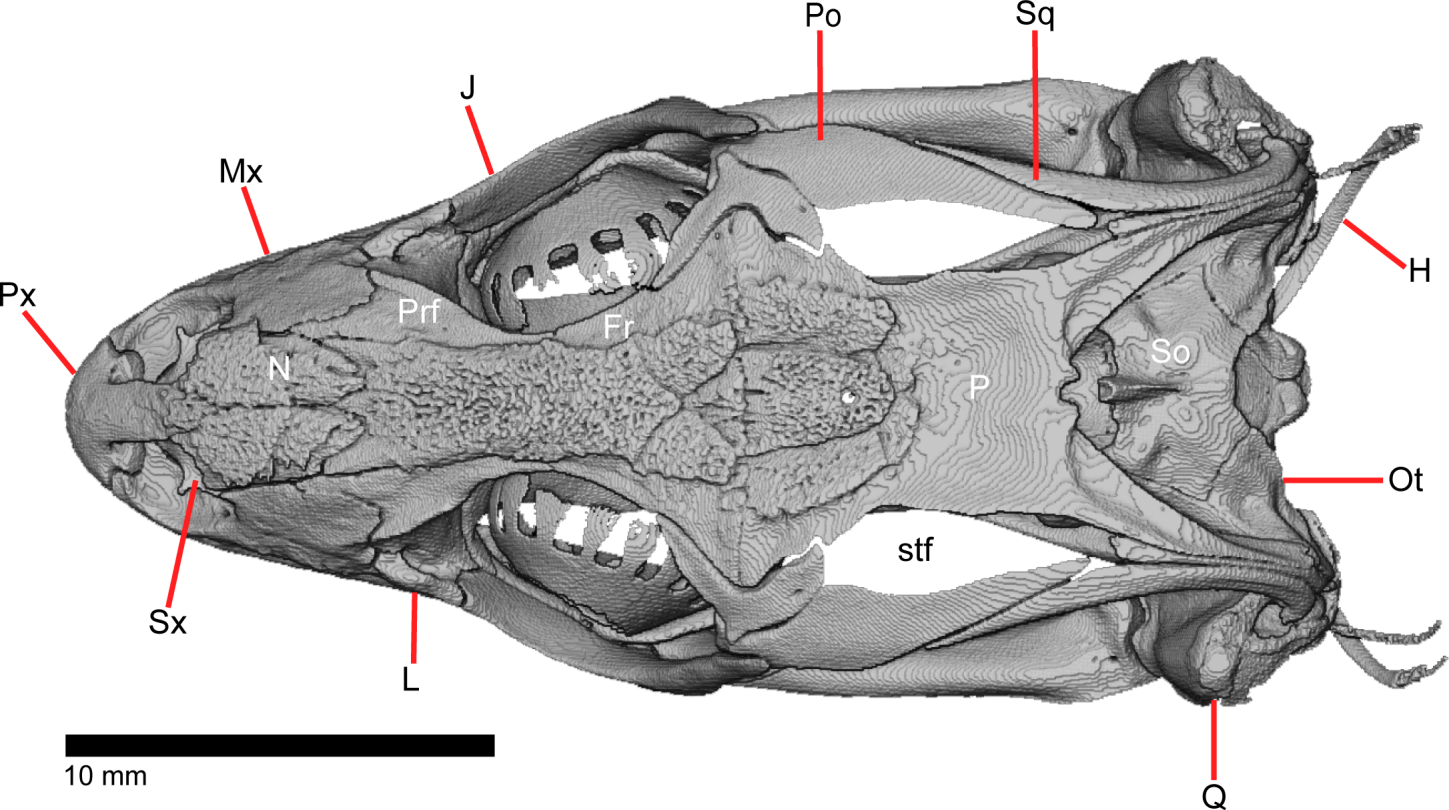
Skull of *Elgaria panamintina* MVZ 191076 in dorsal view, anterior to left.

**Fig 2:**
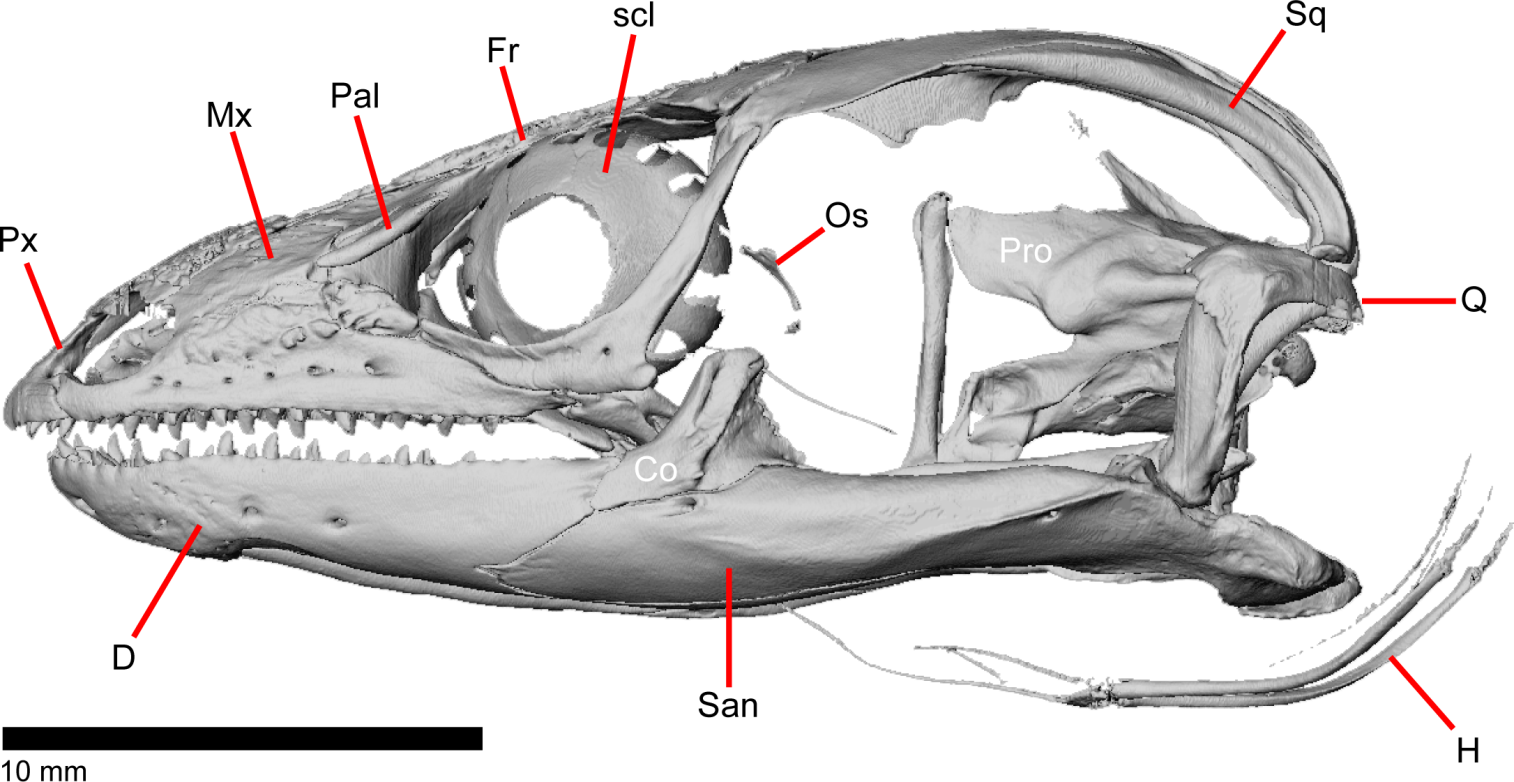
Skull of *Elgaria panamintina* MVZ 75918 in lateral view, anterior to left.

**Fig 3:**
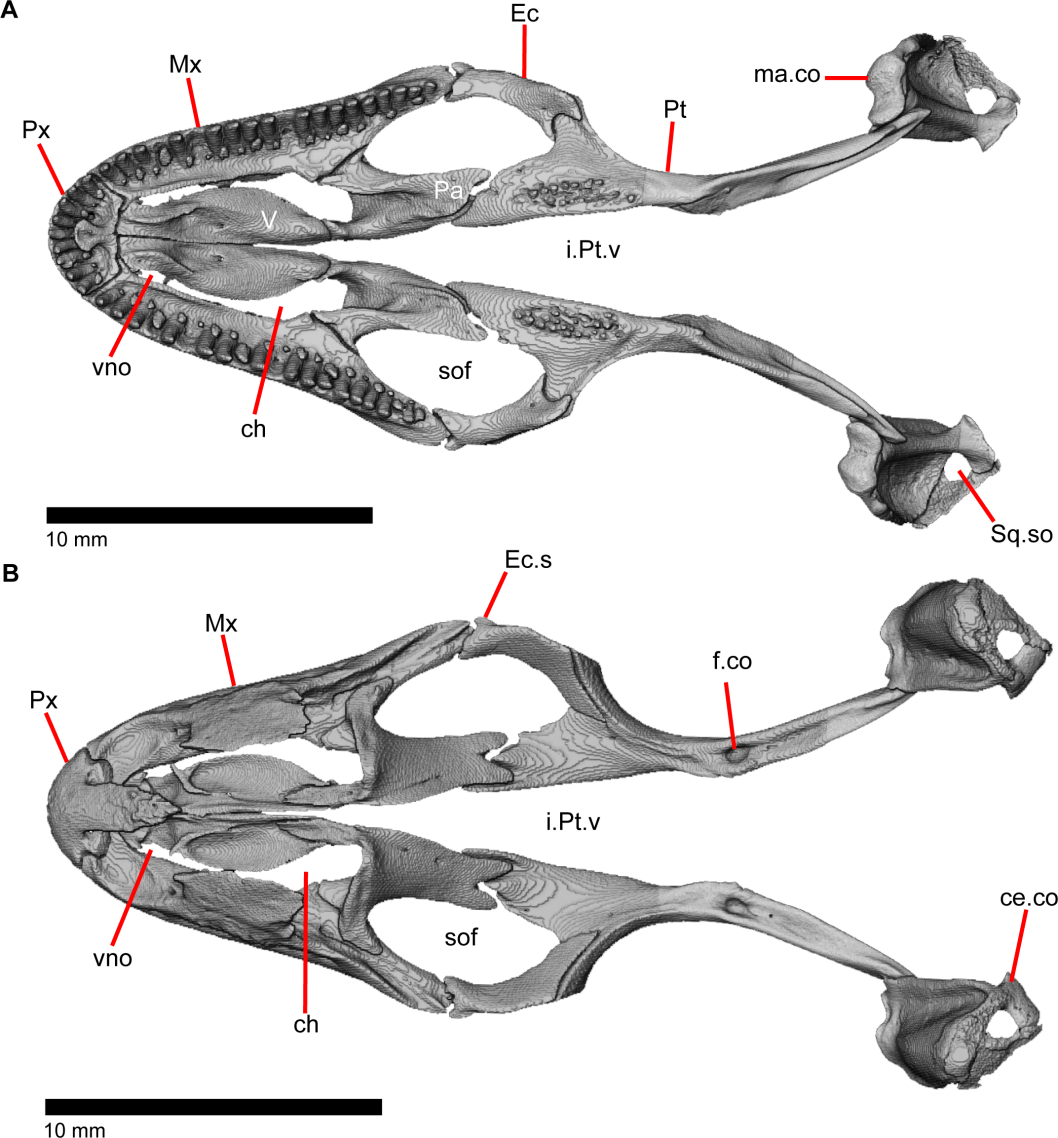
Palatal bones of *Elgaria panamintina* MVZ 191076, anterior to left. (A) Ventral view. (B) Dorsal view.

**Fig 4:**
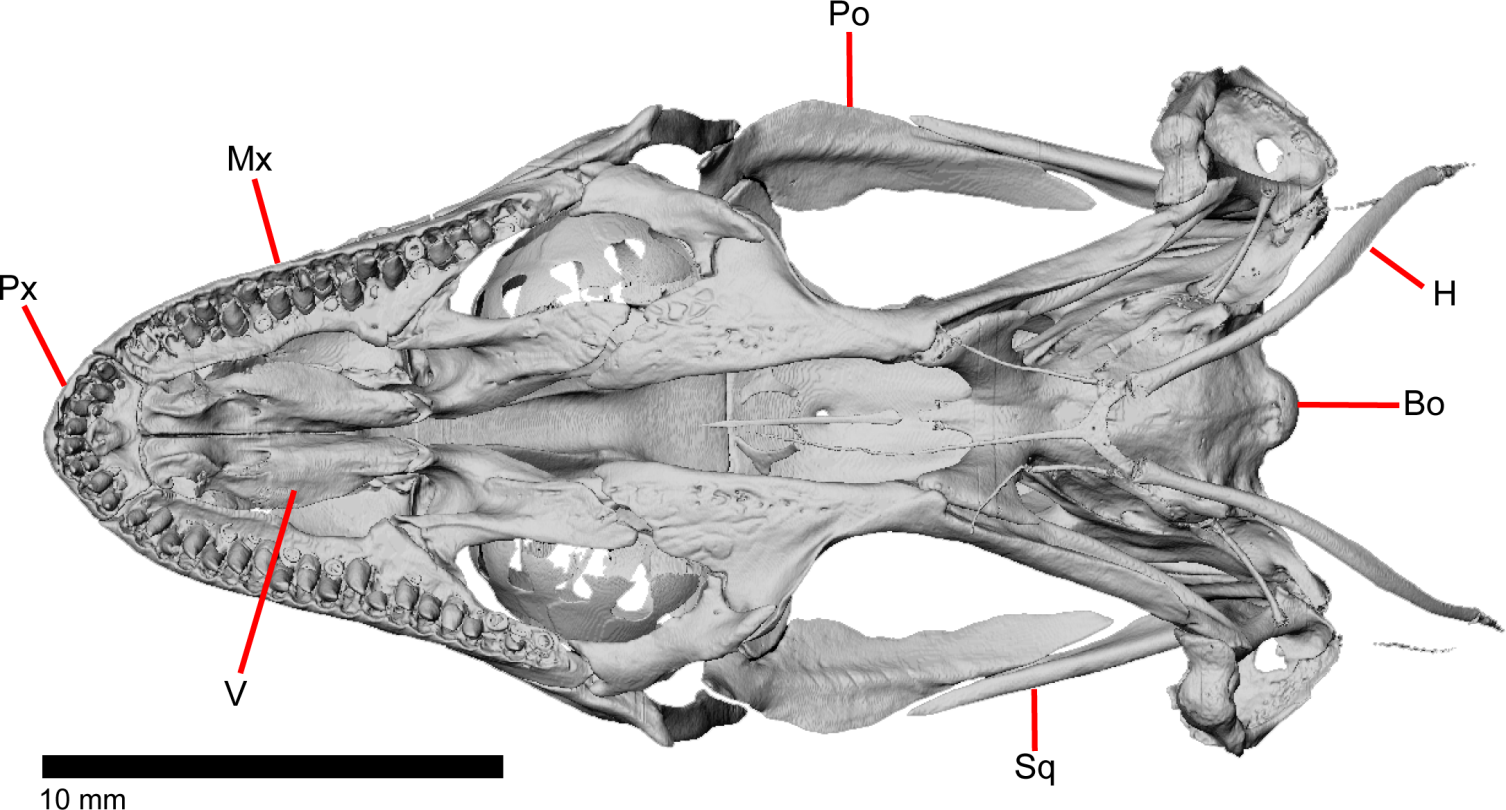
Skull of *Elgaria panamintina* MVZ 75918 in ventral view, anterior to left.

The skull of *Elgaria panamintina* resembles the typical anguid condition of being narrowed and elongated in the preorbital and postorbital regions of the skull (Fig 1) [43]. The supratemporal fenestra is open but mediolaterally compressed, and is bounded laterally by a complete postorbital and supratemporal bar (Figs 1 and 2). Cranial osteoderms are fused to the midline roofing bones including the nasals, the fused frontals, and the anterior portion of the parietal. In palatal view, *Elgaria panamintina* has the incomplete neochoanate condition (Fig 3A and 3B). The suborbital fenestra (inferior orbital foramen of Criley [24]) is a large ovoid shape and the interpterygoid vacuity (pyriform space of Criley [24]) narrows anteriorly and terminates between the posterior ends of the vomers (Figs 3A and 4). *Elgaria panamintina* possesses a single large posttemporal fenestra. The posttemporal fenestra is visibly larger in *Elgaria panamintina* MVZ 75918. The skull of *Elgaria panamintina*, like other anguids, possesses joints that enable amphikinetic movement, and may also be capable of hypokinetic bending [43, 44].

### Dentary

**Fig 5:**
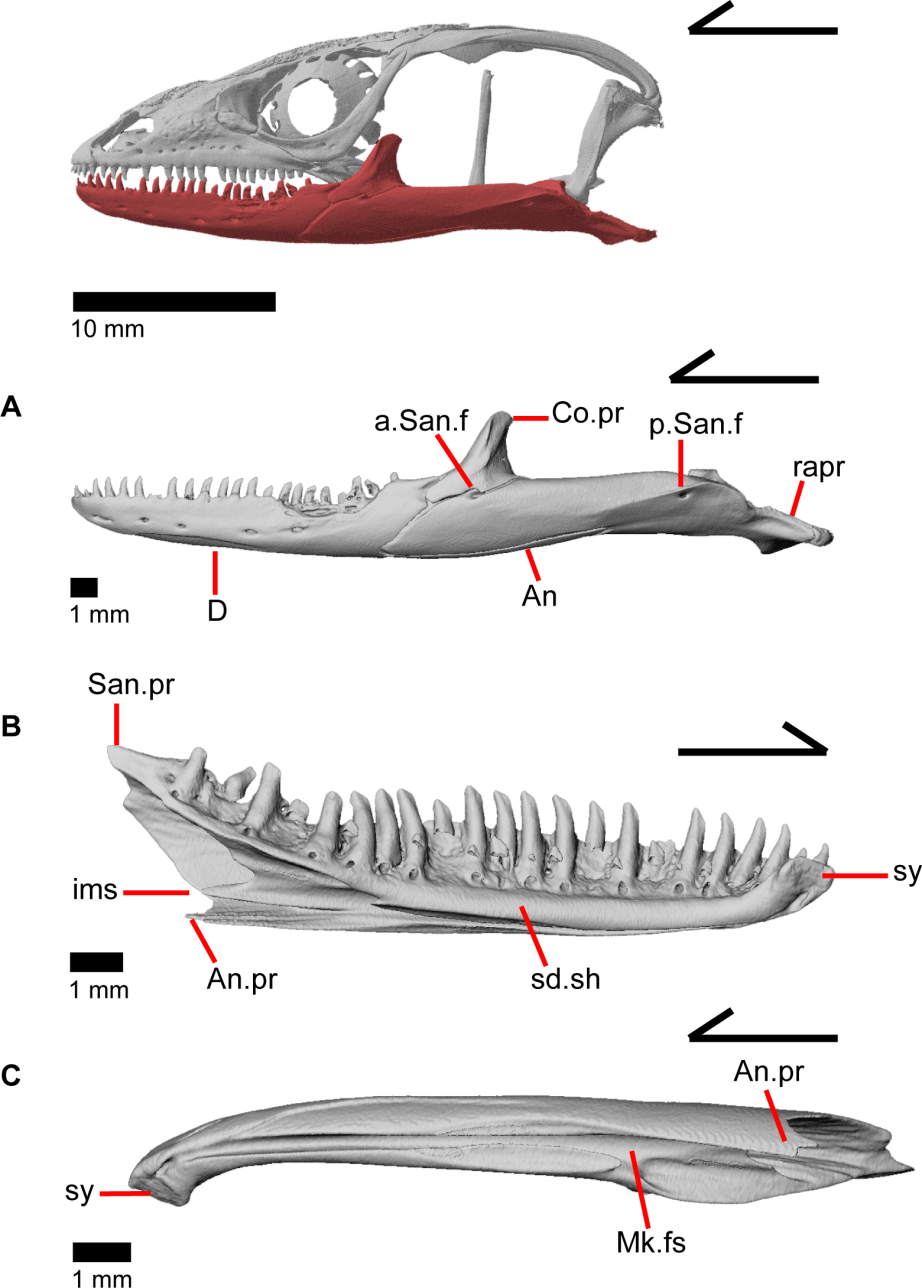
Lower jaw bones of *Elgaria panamintina* MVZ 191076. (A) Left mandible from a lateral view. (B) Left dentary from a medial view. (C) Left dentary from a ventral view.

**Fig 6:**
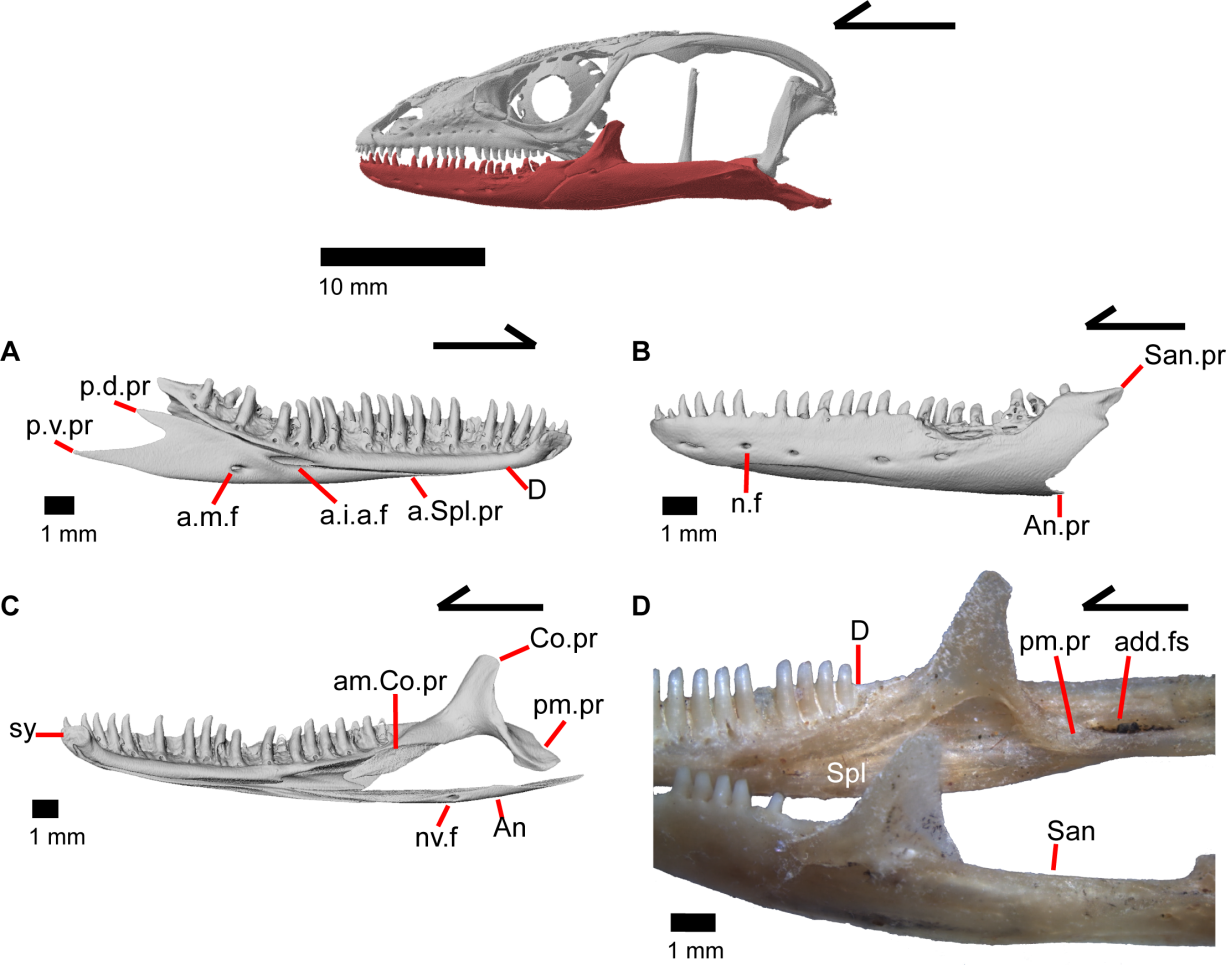
Lower jaw bones of *Elgaria*. (A) Left dentary and splenial of *Elgaria panamintina* MVZ 191076 in medial view. (B) Left dentary of *Elgaria panamintina* MVZ 191076 in lateral view. (C) Some right mandibular bones of *Elgaria panamintina* MVZ 191076 in medial view. (D) Mandibles of *Elgaria multicarinata* TMM M-9004.

The dentary is the large tooth-bearing bone located in the anterior portion of the lower jaw (Figs 2 and 5A). The dentary is robustly built in *Elgaria panamintina*, and has 22–25 tooth positions above an open meckelian fossa, which faces ventrally (Fig 5B and 5C). The splenial obscures the meckelian fossa when the lower jaw is articulated (Fig 6A). The dentary curves inward anteriorly to the symphysial surface, which is relatively small. A free intramandibular septum divides the inferior alveolar canal from the meckelian fossa (Fig 5B) [43]. A medial subdental shelf is present, but no subdental gutter. There are separate angular and surangular processes (dorsal process of the dentary of Evans [43]), but no distinct coronoid processes (Fig 6B). The angular process contacts the angular ventrally, and the surangular process articulates with the surangular medially and the coronoid dorsally. There are four to seven nutrient foramina (mental foramina of Criley [24]) on the anterolateral surface of the dentary.

The left dentaries of both examined specimens of *Elgaria panamintina* were subject to unrelated but significant deformities or pathologies. The right dentaries of both specimens are intact and undamaged, and so can still serve as exemplars of the species and genus. In addition, the dentaries of examined specimens of *Elgaria multicarinata* did not exhibit any bilateral asymmetry with the exception of occasional minor differences in the number of tooth positions. The left dentary of MVZ 75918 appears to have been broken during the lifetime of the animal, but did heal (Fig 2). The anterior end of the bone is enlarged, especially ventrally, and is also crooked as if the bone did not set completely straight during the healing process. The anteriormost end of the left splenial is also slightly enlarged and blunted relative to the other splenial on the same specimen and to both splenials of MVZ 191076.

The other specimen (MVZ 191076) also appears to have a pathology on the left dentary (Fig 6B), but it is less clear whether this abnormality occurred before or after its preparation. Part of the medial posterior part of the bone is significantly eroded. This deformation may be the result of a break during the animal’s life, but also may have resulted from its capture or from erosion by the formalin preservative, which we recognized in other gerrhonotine specimens.

### Coronoid

**Fig 7:**
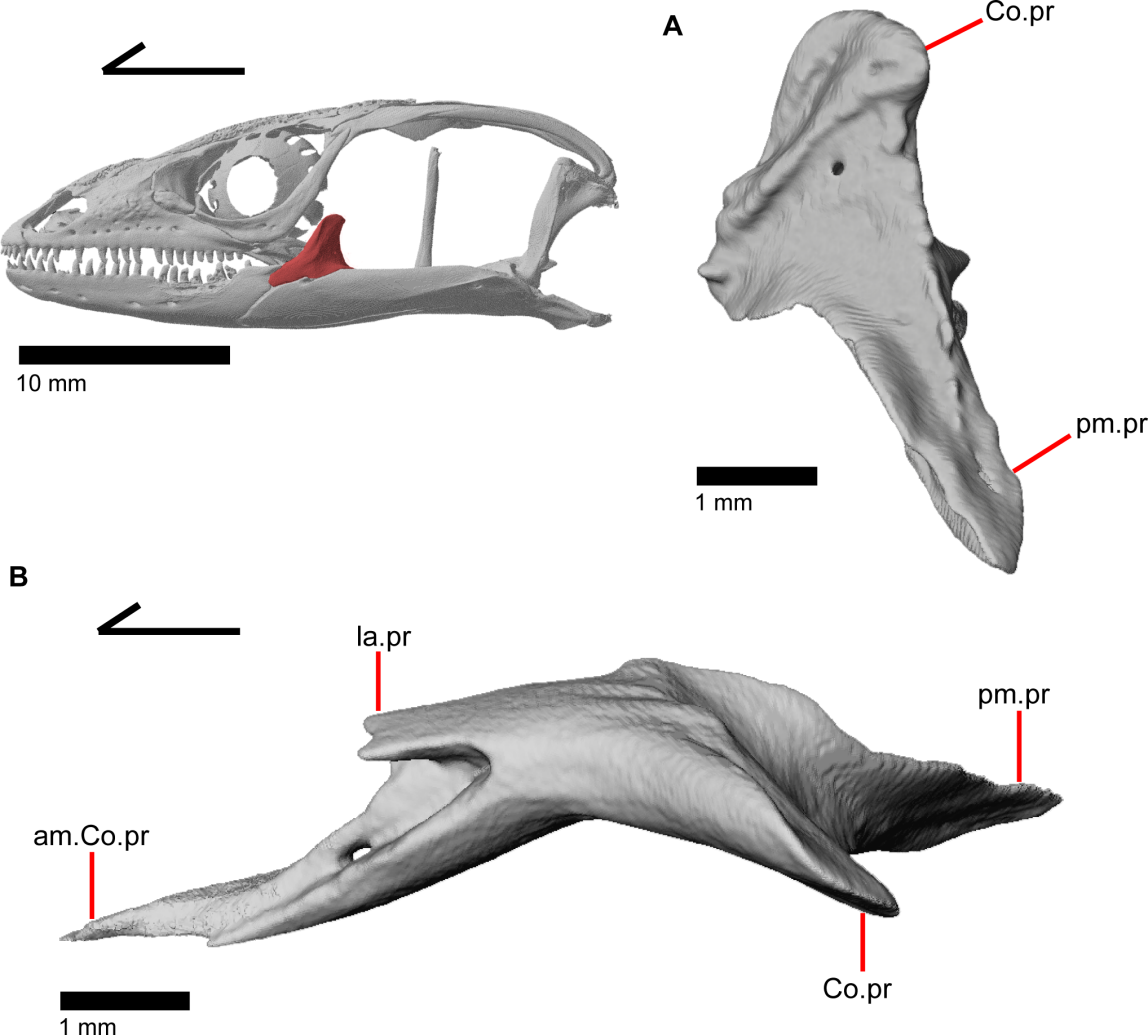
Left coronoid of *Elgaria panamintina*. (A) Left coronoid of *Elgaria panamintina* MVZ 75918 in posterior view. (B) Right coronoid of *Elgaria panamintina* MVZ 191076 in dorsal view.

The coronoid is dorsally located near the midpoint of the mandible. In medial view, the coronoid is a triradiate bone with a dorsal, fin-like coronoid process that is slightly angled posteriorly (Fig 6C). The posterior surface of this fin is slightly excavated for attachments for the adductor muscles [45] and has one to two foramina (Fig 7A). A crest runs down the posterior edge of the coronoid process and onto the anterior end of the posteromedial process (posteroventral process of Good [3]). The anterior portion of the coronoid is bifurcated with a longer anteromedial process and a shorter lateral process (Fig 7B; labial process of Evans [43]). The longer anteromedial process extends down into the posterior meckelian fossa and articulates with the dentary, articular, surangular, and splenial. The process extends anteriorly past the last tooth position on the dentary as previously described in *Elgaria* and *Gerrhonotus* [3], extending to the third or fourth tooth position (Fig 6C). There is a small, distinct facet on the lateral surface of the coronoid for articulation with the splenial. The lateral process of the coronoid is a tongue-like projection that is either rounded, rounded and bifurcated, or pointed. It articulates with a small facet on the lateral side of the dentary (Fig 5A). Together, the anterior lateral and medial processes of the coronoid form a slot into which the posterior end of the dentary fits. Within this slot for the dentary, on the anteromedial process, there is a small foramen that pierces the bone vertically (Fig 7B). The posterior process of the coronoid is draped obliquely onto the lingual surface of the fused prearticular and surangular, as previously described in all gerrhonotines except *Gerrhonotus*, in which the posterior process does not point obliquely [3]. The process extends just posteroventral to the beginning of the adductor fossa (Meckelian foramen of Criley [24]). On MVZ 191076, the lateral surface of the coronoid has a distinct notch so that the coronoid contributes a small amount to the border of the anterior surangular foramen (Fig 5A).

### Articular and surangular

**Fig 8:**
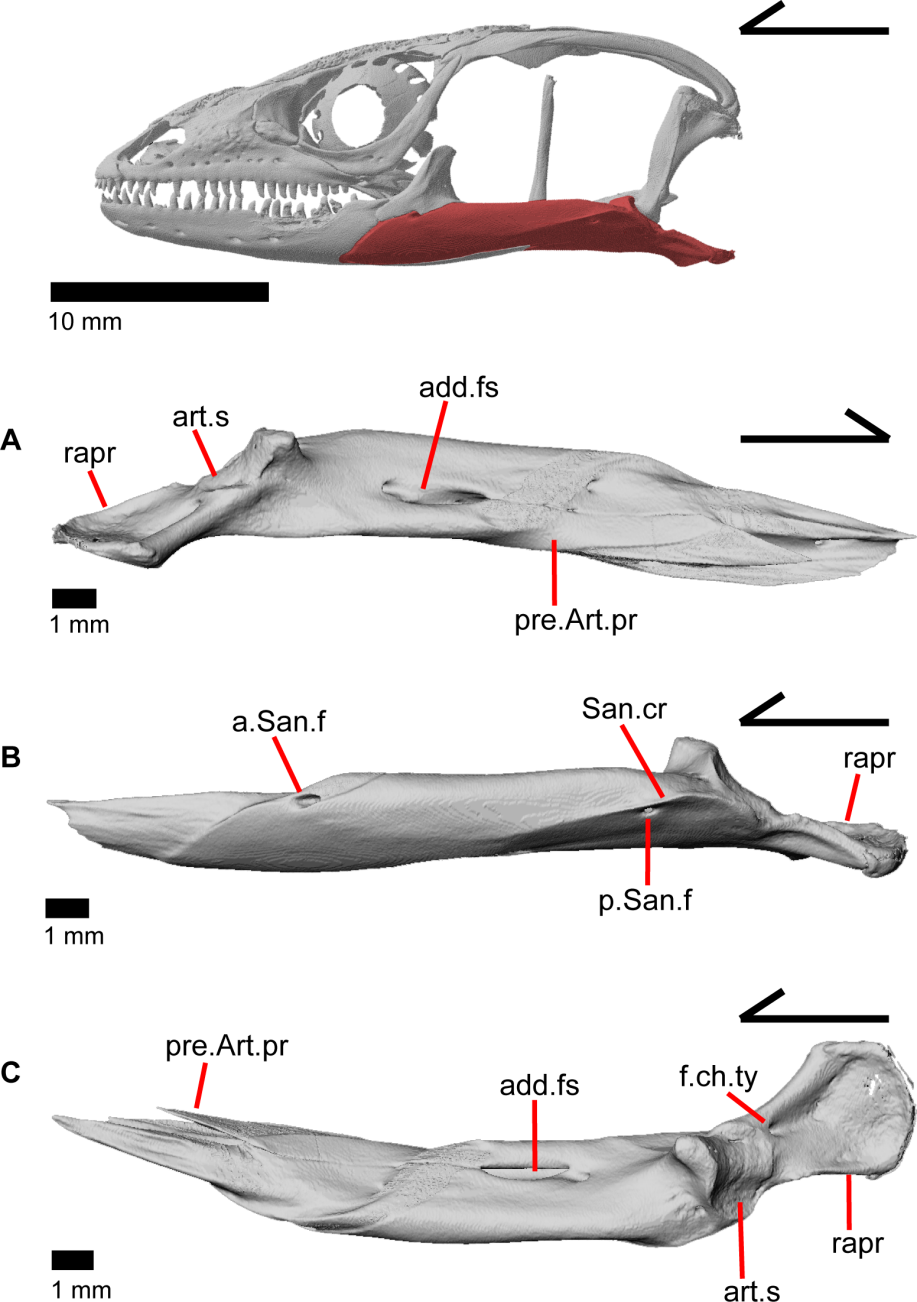
Left surangular+articular of *Elgaria panamintina* MVZ 191076. (A) Medial view. (B) Lateral view. (C) Dorsal view.

The articular and surangular are large and long bones of the mandible that are fused into a single element. However, the anterior projection of the surangular as well as the posterior portion of the articular are distinguishable as separate elements. The suture with the prearticular is visible for a variable portion of the surangular in medial view (Fig 8A). The degree of fusion is most likely modified through ontogeny [43], and we note that most gerrhonotine specimens we examined with CT software appear to have largely or completely fused articular and surangular bones. The fused surangular-articular contacts the dentary anteriorly, the angular ventrally, the splenial anteromedially, the coronoid anterodorsally, and the quadrate posterodorsally. The surangular portion of the fused bone is robust (Fig 8B). There is a large anterior surangular foramen that is contained fully within the surangular in MVZ 75918. In MVZ 191076, the posterior upper margin of the anterior surangular foramen is bounded by the coronoid on both mandibles, and the coronoid has a corresponding notch just above the foramen (Fig 5A). Posterior to the posteromedial coronoid process, an adductor fossa is bounded by the articular and surangular (Fig 8A). The adductor fossa is reduced in anguids relative to both closely related groups such as the Xenosauridae and Shinisauridae and relative to more distantly related taxa like the Teiidae, Xantusiidae, and Iguania [43]. A large posterior surangular foramen (posterior suprangular foramen of Oelrich [46]) is present on the surangular just underneath the surangular crest (Fig 8B). The retroarticular process is long and widens posteriorly, with a depression occupying most of the dorsal surface (Fig 8C). Anterior to that depression is the articular surface for the quadrate. The retroarticular process is similar to other anguids in that it is large, curves inwards, and is broadened [43]. There is a foramen for the chorda tympani branch of the facial nerve on the dorsal surface of the retroarticular process (Fig 8C). Some authors [3] noted that the term “articular” is often incorrectly used in the literature to refer to the fused articular and prearticular. The articular is a small element near the condylar surface for the skull, and the larger element containing the retroarticular process is actually the prearticular [3].

### Angular

The angular is a long, slender bone that ossifies along the ventral portion of the lower jaw, lying under and medial to the main body of the articular and surangular. The angular articulates with the dentary ventral to the intramandibular septum and dorsal to the angular process of the dentary (Fig 6C). The anterior end articulates laterally with the splenial. Posteriorly, the angular articulates posteroventrally with the splenial, becoming visible under the splenial in medial view. Viewed dorsally, it flattens and curves upwards at the anterior and posterior points of the bone. A neurovascular foramen with distinct medial and lateral openings penetrates the bone (Fig 6C). This foramen was previously reported to be absent in gerrhonotine lizards [24].

### Splenial

The splenial is located medially along the mandible and is a slender triradiate bone (Fig 6A). The anterior process of the splenial fills the posterior portion of the meckelian fossa, and terminates below and slightly anterior to the midpoint of the dentary, below tooth positions 13–15. Midway along the length of the bone, a dorsal, well-developed and anteriorly pointing projection fits under the subdental shelf and results in the splenial contributing to much of the dorsal and ventral bordering of the oval-shaped anterior inferior alveolar foramen. Slightly ventral and posterior to that foramen, the splenial is pierced by a smaller and more rounded anterior mylohyoid foramen. Posteriorly, the splenial widens and splits into two processes. The posteroventral process extends farther posteriorly relative to the posterodorsal process. A laterally projecting shelf on the splenial articulates dorsally with the angular. The prearticular process of the fused articular and surangular articulates with the posterior portion of the splenial just dorsal to the shelf. Below the lateral shelf, the angular runs 60% the length of the splenial. The posterodorsal process of the splenial contacts the medial surface of the anterior lingual process of the coronoid and the surangular. The posterodorsal and posteroventral processes create a notch that is rounded and slightly pointed.

### Dentition

**Fig 9:**
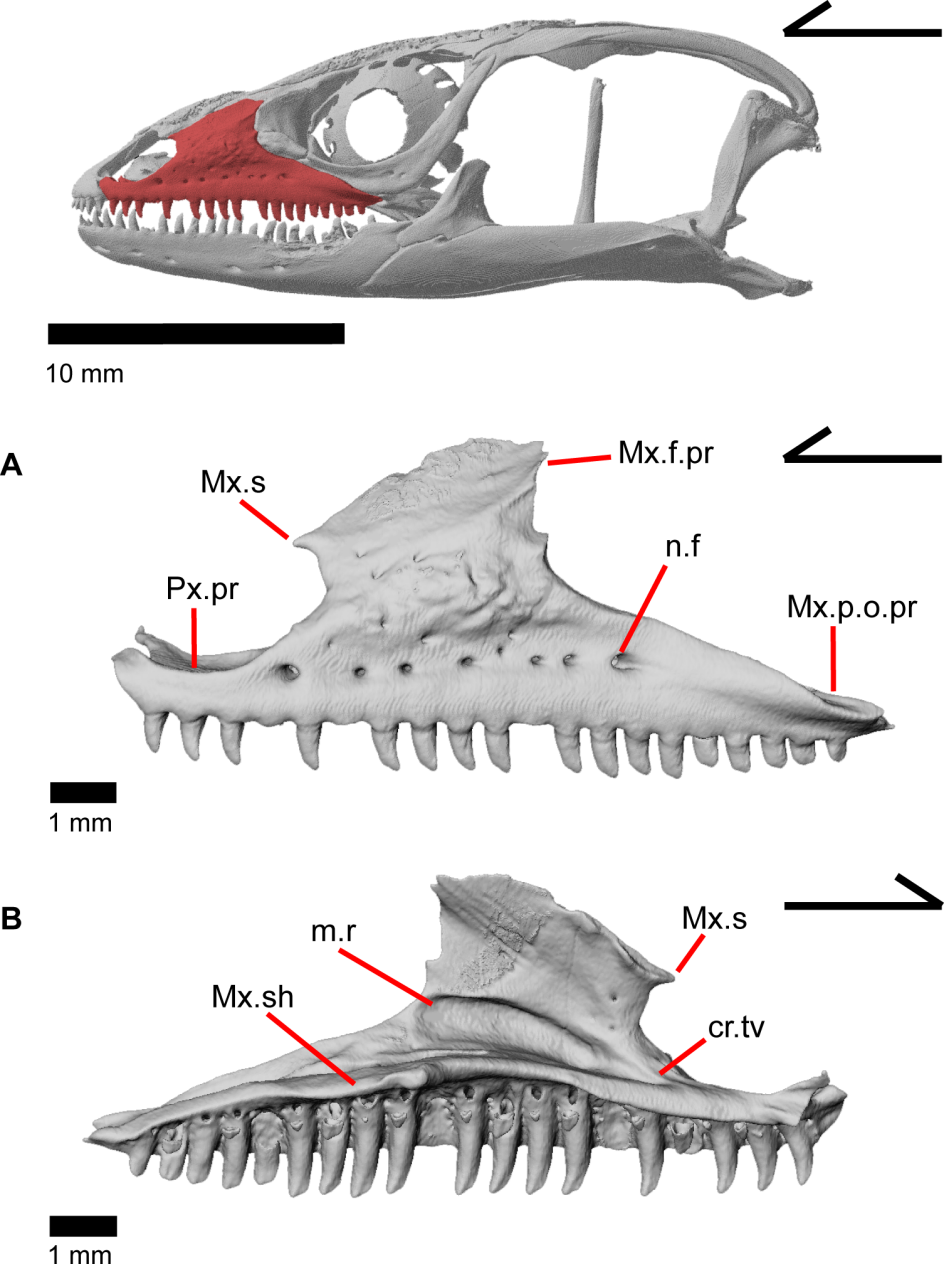
Left maxilla of *Elgaria panamintina* MVZ 191076. (A) Lateral view. (B) Medial view.

**Fig 10:**
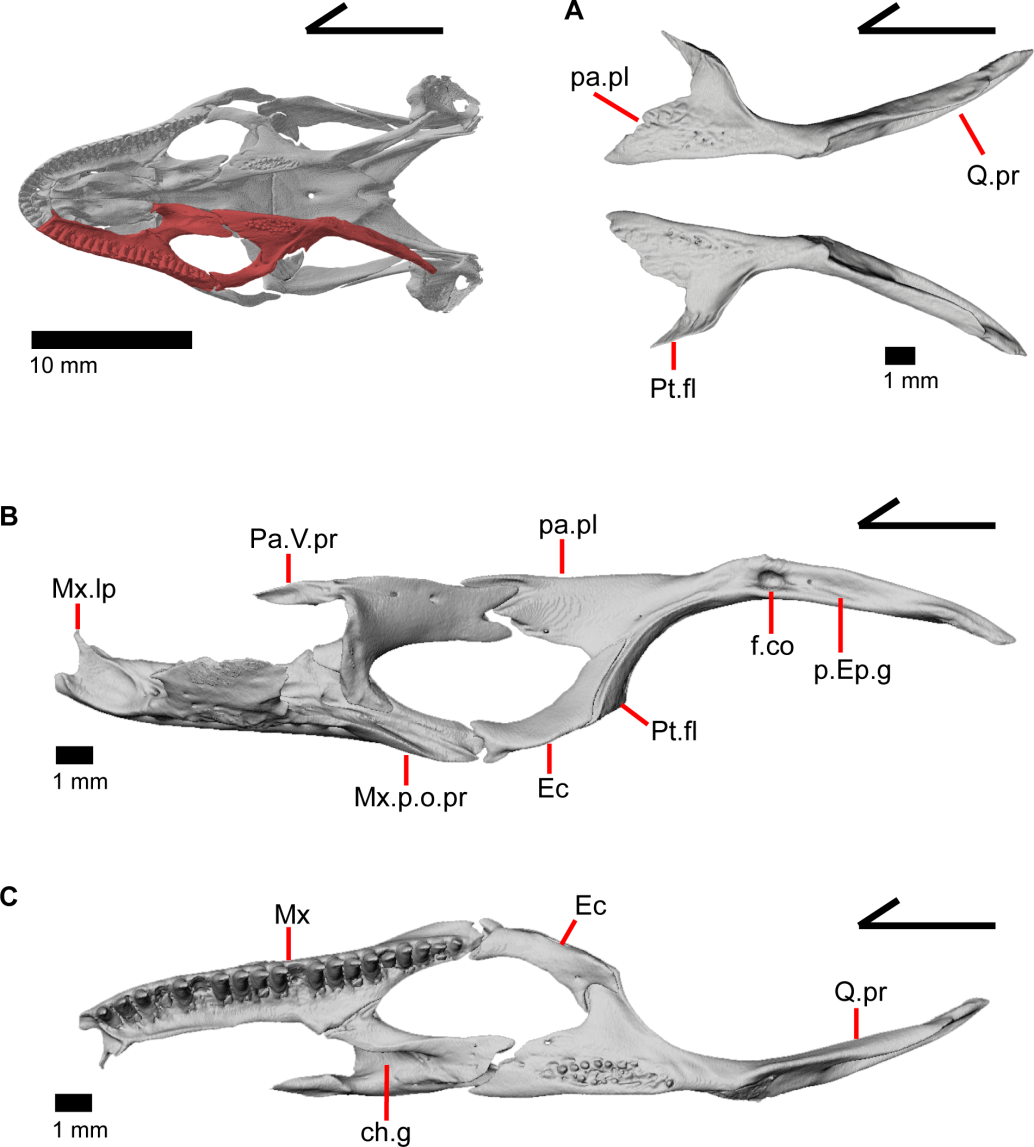
Some palatal bones of *Elgaria panamintina*. (A) Pterygoids of *Elgaria panamintina* MVZ 75918 in ventral view. (B) Some palatal bones of *Elgaria panamintina* MVZ 191076 in dorsal view. (C) Some palatal bones of *Elgaria panamintina* MVZ 191076 in ventral view.

*Elgaria panamintina*, like all anguids, exhibits pleurodont tooth insertion with lingual replacement. The teeth are slightly to moderately recurved (Figs 5B, 9A and 9B). Some teeth, especially distal teeth, are relatively straight and blunt. Teeth are typically unicuspid, although distal teeth on the dentary and the maxilla resemble the bicuspid condition that was previously described in gerrhonotines [26]. Pterygoid teeth are absent in MVZ 75918 (Fig 10A), contrary to previous studies in which pterygoid teeth were present in all examined specimens of *Elgaria* [1–3]. Teeth do not appear to have been removed or broken off, but small pits and grooves are present in the location where teeth would normally be located.

### Premaxilla

**Fig 11:**
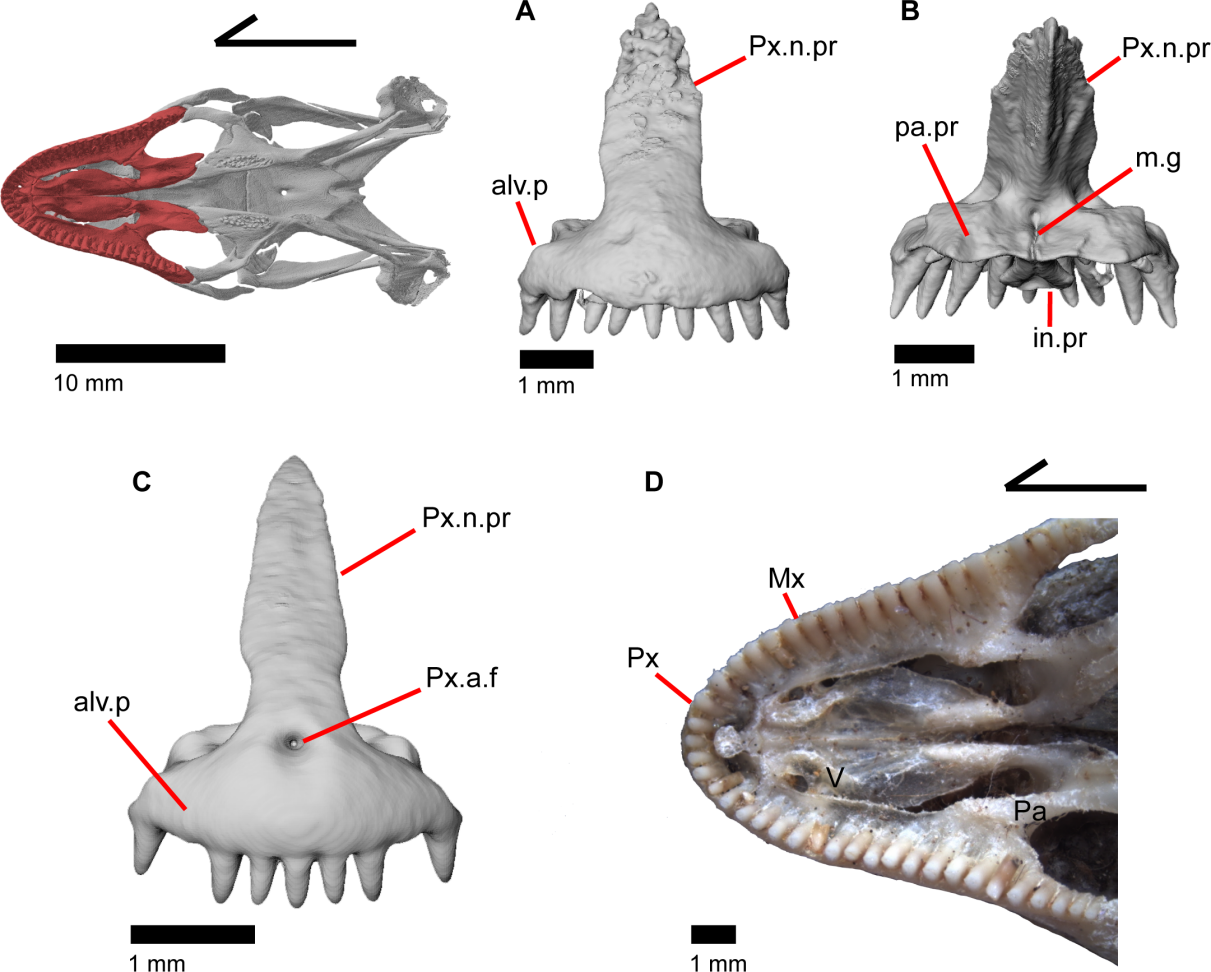
Some anterior palatal bones of *Elgaria*. (A) Premaxilla of *Elgaria panamintina* MVZ 191076 in anterior view. (B) Premaxilla of *Elgaria panamintina* MVZ 191076 in posterior view. (C) Premaxilla of *Elgaria multicarinata* TNHC 4478 in anterior view. (D) Anterior palatal elements of *Elgaria multicarinata* TMM M-9004 in ventral view.

**Fig 12:**
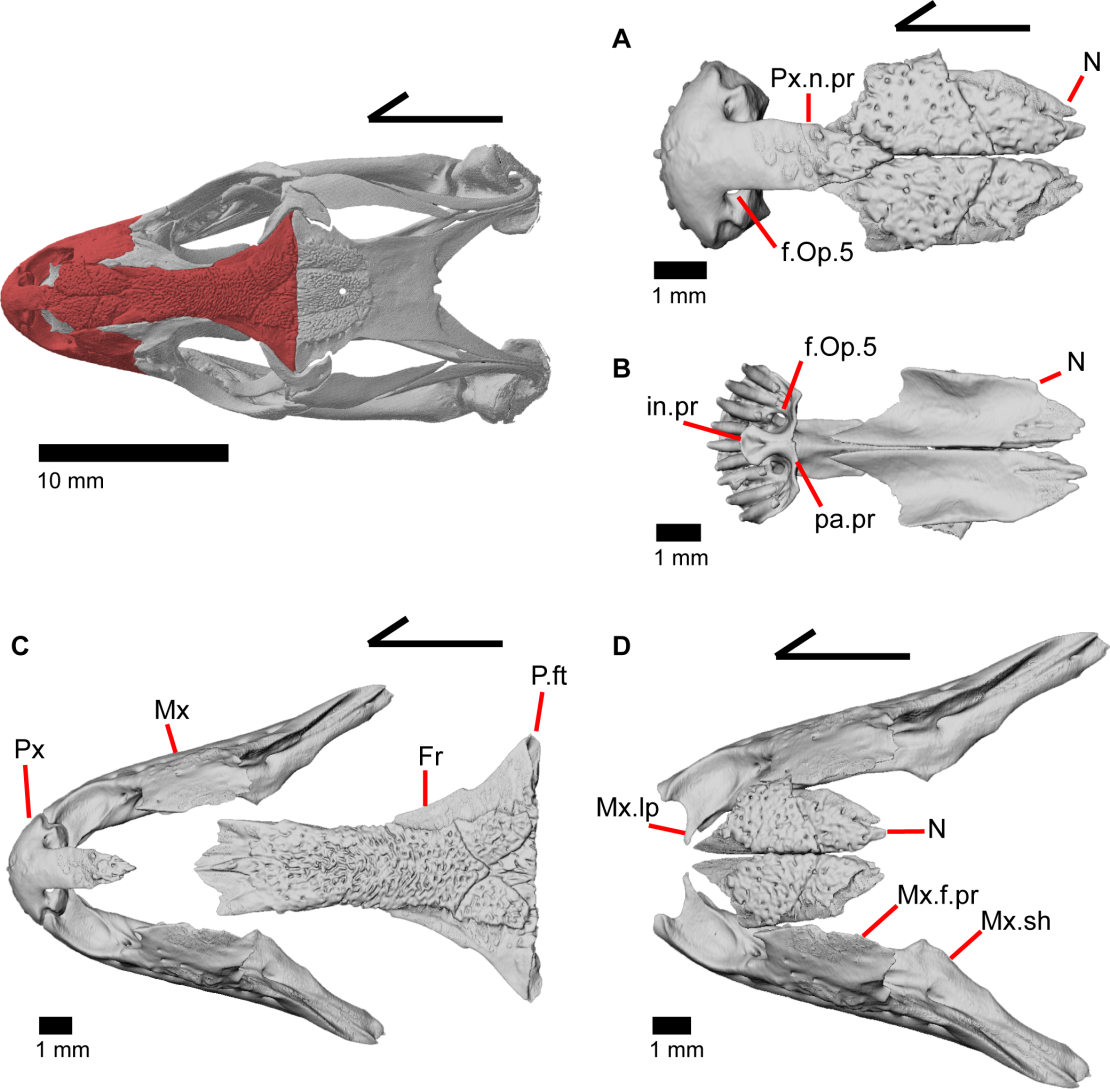
Some tooth bearing and anterior roofing bones of *Elgaria panamintina* MVZ 191076. (A) Premaxilla and nasals of *Elgaria panamintina* MVZ 191076 in dorsal view. (B) Premaxilla and nasals of *Elgaria panamintina* MVZ 191076 in ventral view. (C) Premaxilla, maxillae, and frontal of *Elgaria panamintina* MVZ 191076 in dorsal view. (D) Maxillae and nasals of *Elgaria panamintina* MVZ 191076 in dorsal view.

**Fig 13:**
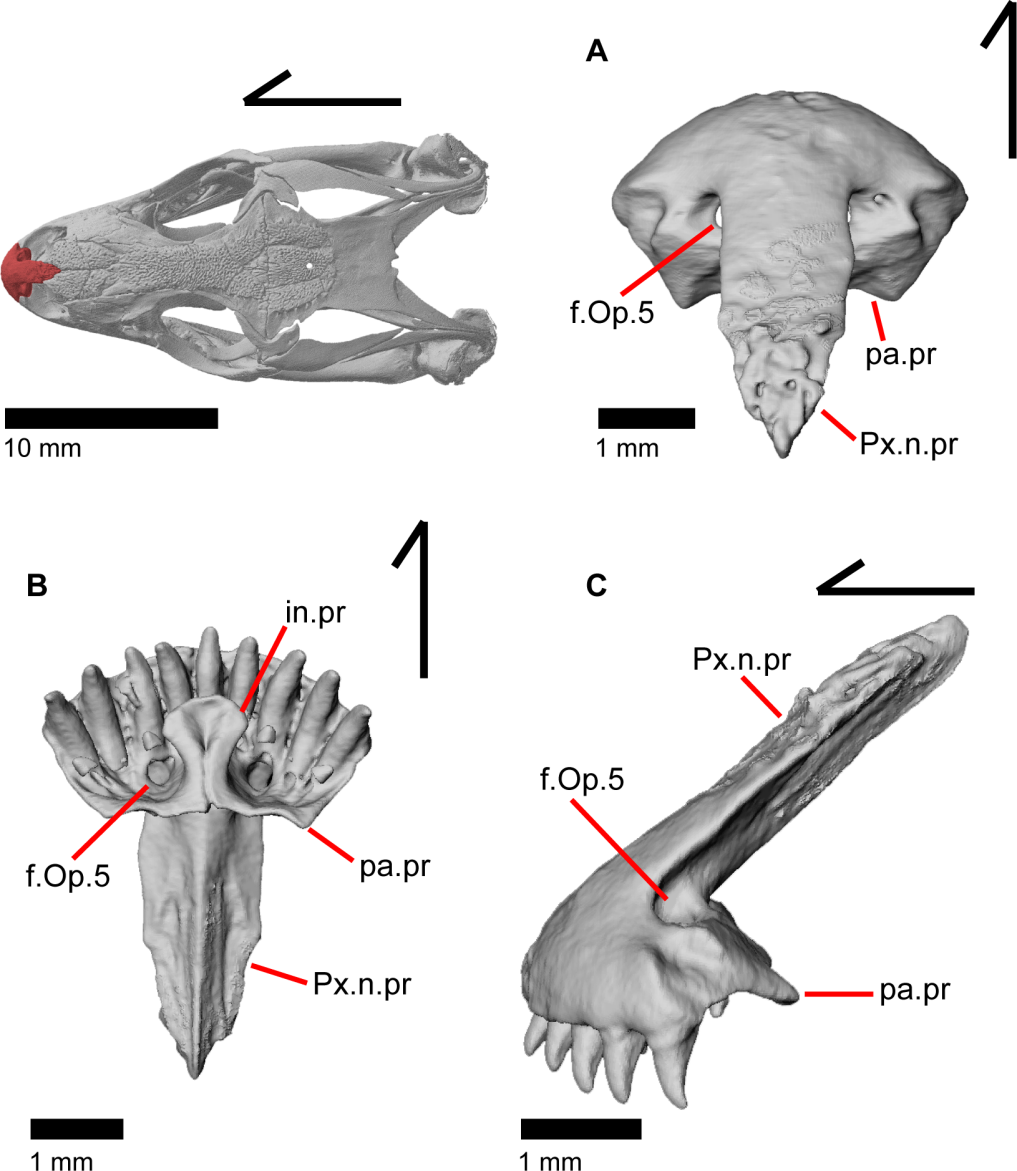
Premaxilla of *Elgaria panamintina* MVZ 191076. (A) Dorsal view. (B) Ventral view. (C) Lateral view.

The premaxilla is the anteriormost tooth-bearing bone of the upper jaw. It is a single midline element; the paired premaxillae fuse prior to hatching [47]. The tooth-bearing alveolar plate (Fig 11A) possesses nine tooth positions in MVZ 191076, but only eight tooth positions in MVZ 79518. A midline nasal process projects posterodorsally to articulate dorsally with the nasals (Fig 12A and 12B). A midline ridge occurs on the ventral surface of the nasal process extending from the base of the nasal process to its posterior tip (Fig 11B). Osteoderms are often fused to the dorsal surface of the nasal process, especially on the posterior end. The nasal process has a relatively uniform width for much of its length but the posterior portion tapers rapidly to terminate in a slightly pointed tip. The posterior tip of the nasal process does not contact the frontal (Fig 12C) [3], and there is no constriction at the base of the nasal process. Just lateral to the connection between the nasal process and the main body of premaxilla are two large medial foramina that pierce straight down through the bone (Fig 12A and 12B). In other lizards, these foramina transmit the ophthalmic branch of CN5 [46]. On the right side of the premaxilla of MVZ 191076, there is an additional smaller foramen located just lateral to the foramen for ophthalmic branch of CN5 (Fig 13A).

Posteriorly, the main body of the premaxilla has a short and inclined shelf-like palatal process on which a medial groove demarcates the suture between the fused palatal processes (Fig 11B) [47]. The vomers articulate with the posterior edge of this shelf. A medial maxillary lappet hugs the palatal process dorsal to the articulation with the vomers. The edge of the palatal process is slightly wavy in ventral view (Fig 13B) and the two lateral corners of the shelf fit in a facet on the ventral surface of the maxilla. The dorsolateral surface of the alveolar plate bears a distinct depression that serves for articulation with the premaxillary process of the maxilla (Fig 13C). The premaxilla has an anteriorly projecting lobe-like incisive process, originating from the ventral surface of the palatal process and protruding anteroventrally (Fig 13B). In MVZ 191076, the ventral surface of the incisive process has a medial groove that is a continuation of the groove on the palatal process (Fig 11B). The groove widens in MVZ 191076 into a concavity [3], which is found on both specimens on the ventral surface of the incisive process. The premaxilla lacks a foramen on the anterior surface of the alveolar plate.

### Maxilla

**Fig 14:**
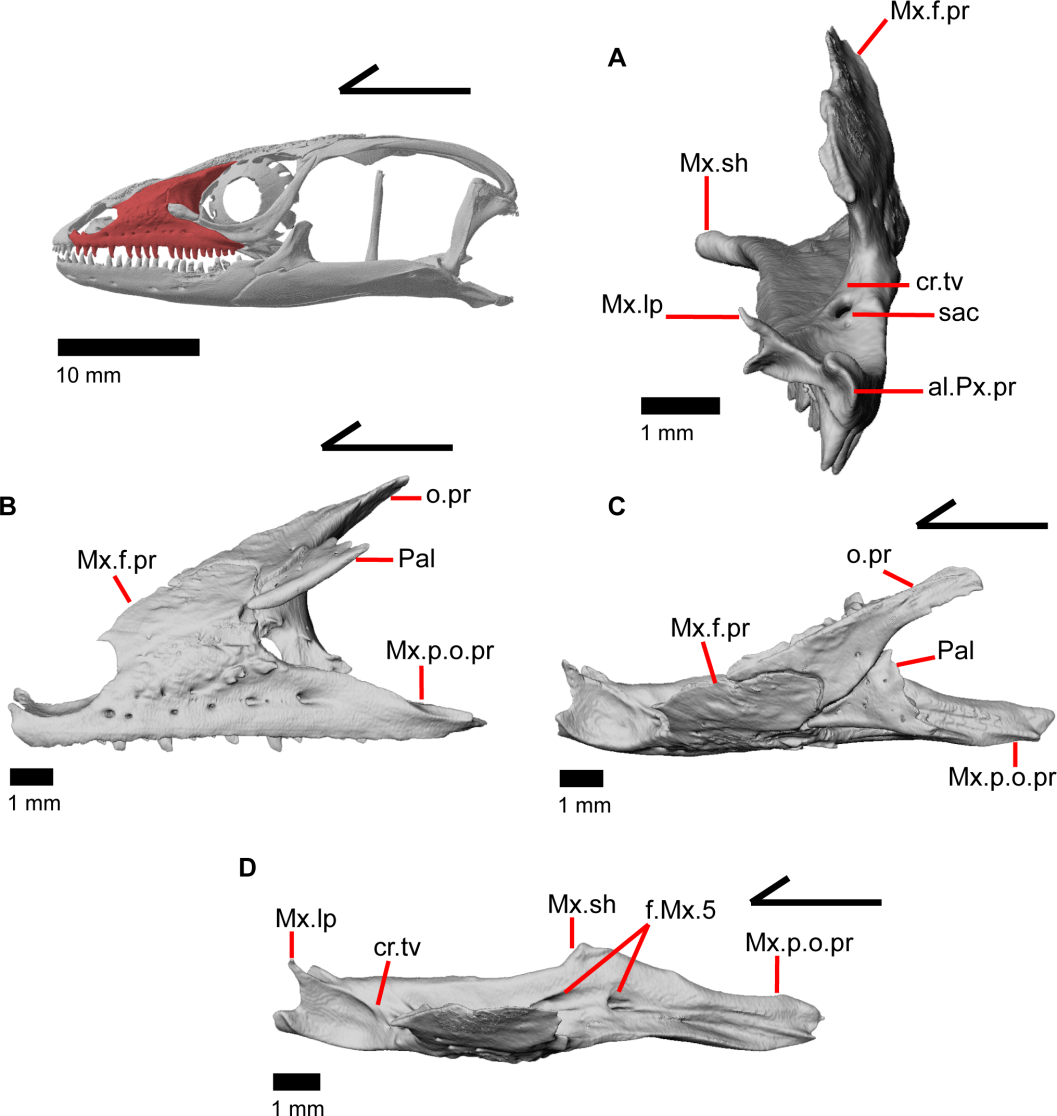
Some anterolateral bones of *Elaria panamintina*. (A) Left maxilla of *Elgaria panamintina* MVZ 191076 in anterior view. (B) Left maxilla, prefrontal, and palpebral of *Elgaria panamintina* MVZ 75918 in lateral view. (C) Left maxilla, prefrontal, and palpebral of *Elgaria panamintina* MVZ 75918 in lateral view. (D) Left maxilla of *Elgaria panamintina* MVZ 191076 in dorsal view.

The maxilla is a triradiate tooth-bearing bone of the upper jaw that has orbital, facial, and premaxillary processes (Fig 9A). There are 19–21 maxillary tooth positions (Fig 9B). The premaxillary process is bifurcated (Fig 14A) with an anteromedial maxillary lappet that rests on the shelf of the premaxilla, almost contacting the maxillary lappet of the other maxilla near the midline (Fig 12D). Just posteroventral to the maxillary lappet, the maxilla articulates with a facet on the anterolateral surface of the vomer. The short anterolateral lappet of the premaxillary process as well as the base of the maxillary lappet bear a ventral facet for articulation with the lateral portion of the palatal process of the premaxilla.

The facial process (nasal process of Criley [24]) is a dorsal, medially curving process that is oriented along much (about a third) of the total length of the maxilla. A purportedly unambiguous synapomorphy of *Elgaria* was previously described on the facial process of the maxilla, “wherein the midpoint of the apex lies posterior to the midpoint of the maxilla itself” [2]. We did not find this condition in either specimen of *Elgaria panamintina*. In both specimens, the midpoint of the apex was anterior to the midpoint of the maxilla. The posterior portion of the facial process is taller than the anterior part and has either a rounded “u” or a “v” shaped contact with the prefrontal (Fig 14B and 14C), as previously documented in all gerrhonotines besides *Gerrhonotus* and *Abronia* [3]. The maxilla does not contact the frontal but does contact the nasal along the anterior half of the facial process (Fig 12D), and the prefrontal for the posterior half of the process. At the anteriormost contact with the nasal, there is a small, triangle-shaped spur extending anteriorly from the facial process (Fig 9A). A weak inclination of the anterior margin of the facial process is an allegedly unambiguous synapomorphy of *Elgaria* [2], but we found that the facial process is moderately to steeply inclined in *Elgaria panamintina*. The crista transversalis [36] is a relatively low ridge located medially on the premaxillary process. Posteriorly, the crista transversalis merges with the facial process. The medial recess for the nasal sac (Fig 9B) (posterior depression on medial surface of facial process of Evans [43]) is found just posterior to the crista transversalis, and extends most of the rest of the length of the facial process.

**Fig 15:**
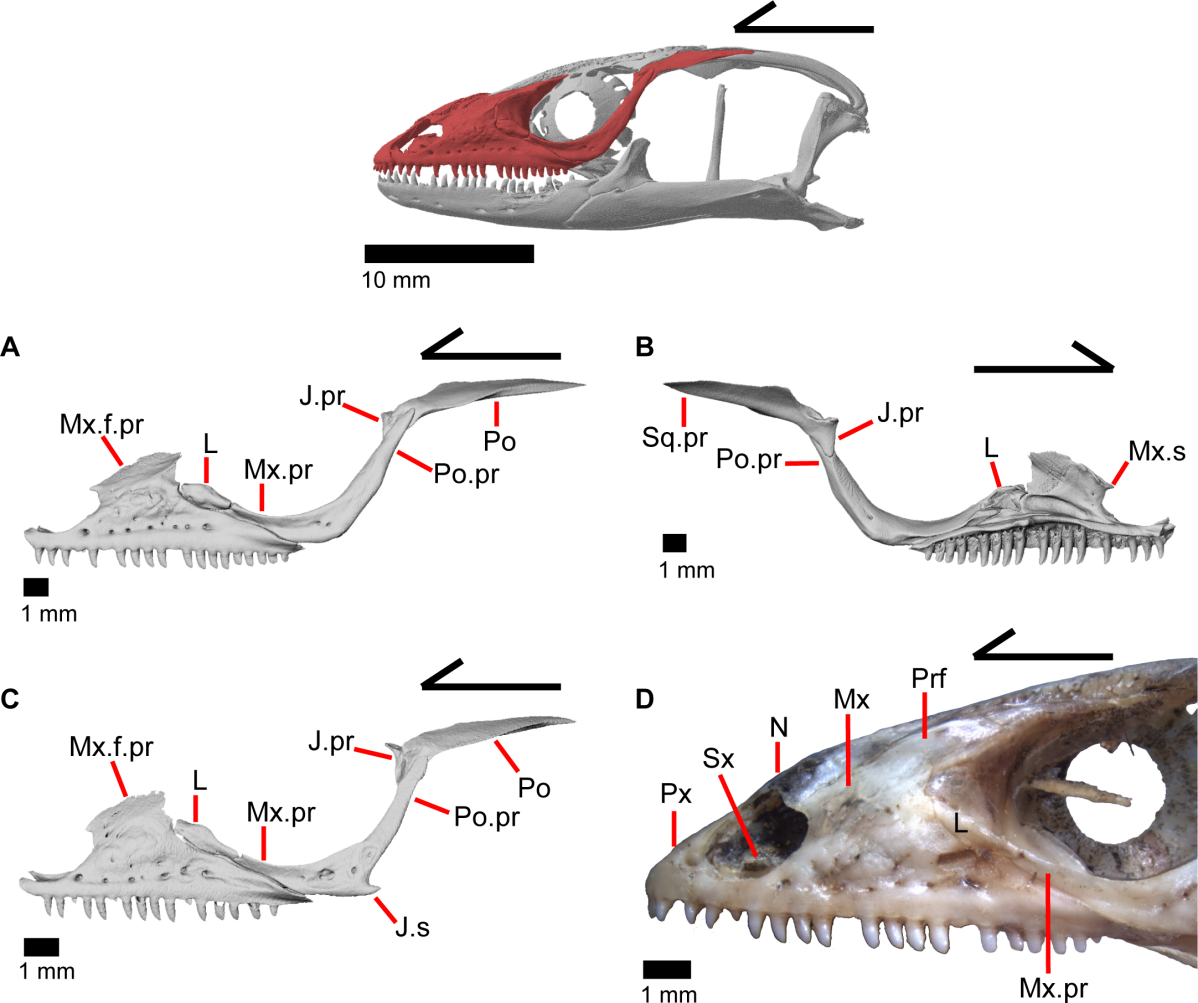
Some anterolateral and temporal bones of *Elgaria*. (A) Left maxilla, lacrimal, jugal, and postorbital of *Elgaria panamintina* MVZ 191076 in lateral view. (B) Left maxilla, lacrimal, jugal, and postorbital of *Elgaria panamintina* MVZ 191076 in lateral view. (C) Left maxilla, lacrimal, jugal, and postorbital of *Elgaria multicarinata* TNHC 4478 in lateral view. (D) Snout of *Elgaria multicarinata* TMM M-9005 in lateral view.

The orbital process of the maxilla extends the posterior half of the maxilla beneath the orbit, and gradually tapers posteriorly. The dorsal surface of the orbital processes has a facet bordered by a lateral ridge for contact with the jugal (Figs 14D, 15A and 15B). In lateral view, the maxilla is excluded from contributing to the border of the orbit by the jugal and the lacrimal. The posteriormost tip of the orbital process of the maxilla contacts the anterior facet of the ectopterygoid (Fig 10B).

The lateral alveolar margin has six or seven perforate nutrient foramina (supralabial foramina in Criley [24] and Good [3]) in a single line, and a few additional foramina are positioned more dorsally on the facial process (Fig 9A). The maxillary shelf (palatal shelf of maxilla in Evans [43]) above the tooth row is roughly the same width until it broadens to articulate with the palatine, and posteriorly narrows (Fig 10C). The vertical thickness of the shelf is variable, but for the most part is relatively thick posteriorly and relatively thin anteriorly. Dorsally, two large foramina (maxillary trigeminal foramina) are visible just anterior to the contact with the palatine (Fig 14D). Just anterior to the anterior edge of the facial process is the opening for the superior alveolar canal (Fig 14A). The maxilla possesses a moderate degree of dermal sculpturing on the lateral surface of the facial process.

### Nasal

The nasal is a thin, paired bone with much of the dorsal surface covered by fused osteoderms (Fig 12A and 12B). The nasal meets its contralateral element medially, the nasal process of the premaxilla anteromedially, the maxilla laterally, the prefrontal posterolaterally, and the frontal posteriorly. On the anterior end of each nasal is an anteroventrally oriented and pointed projection that lies under the nasal process of the premaxilla. Those projections on the nasals slightly diverge at their anterior ends where they are separated by the medial ridge on the premaxillary nasal process. The nasals contact one another medially for most of their length, but the anterior ends and posterior ends are separated by the premaxilla and frontal, respectively. The lateral edge of the nasal contacts the anterior portion of the facial process of the maxilla (Fig 12D) as well as the anterior portion of the prefrontal. The posterior end of each nasal sits on top of paired nasal facets on the frontal. The nasal is concave ventrally except for a raised lateral surface next to the contact with the maxilla, possibly reflecting the underlying nasal capsule shape (Fig 12B) [46].

### Frontal

**Fig 16:**
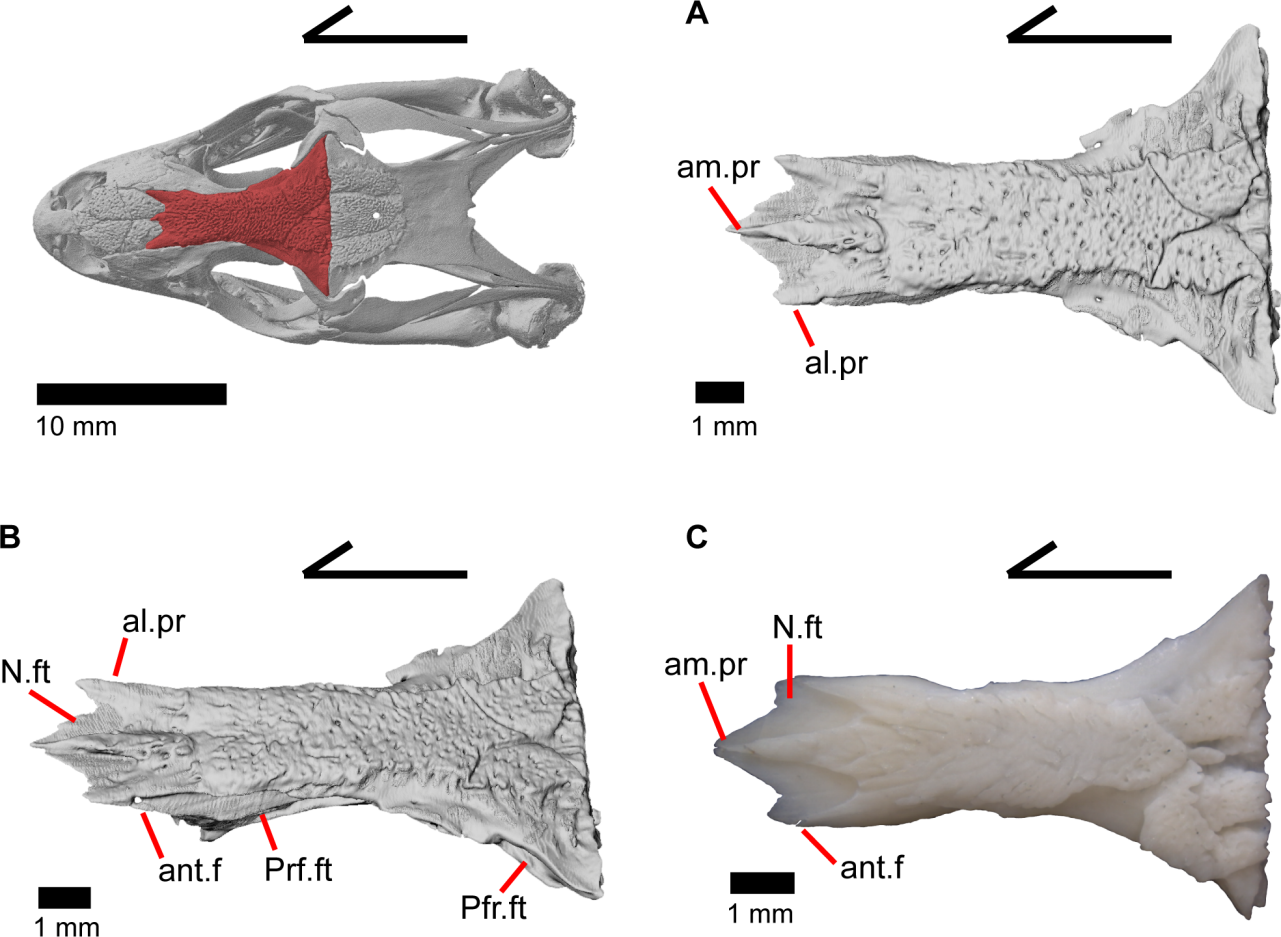
Frontal of *Elgaria*. (A) Frontal of *Elgaria panamintina* MVZ 75918 in dorsal view. (B) Frontal of *Elgaria panamintina* MVZ 75918 in dorsolateral view. (C) Frontal of *Elgaria multicarinata* TMM M-8988 in dorsal view.

**Fig 17:**
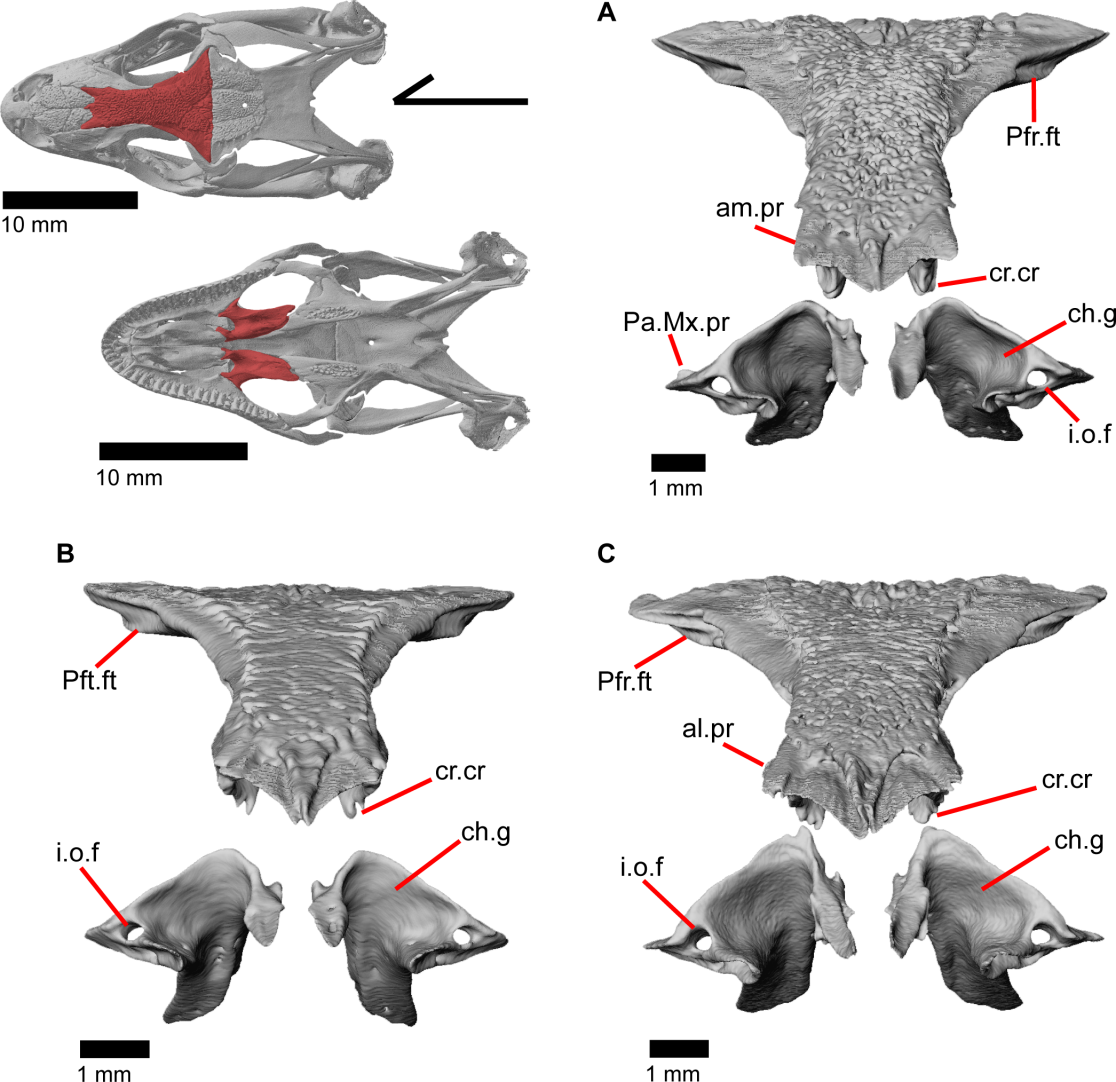
Frontal and palatines of *Elgaria*. The lower reference skull is in ventral view. (A) Frontal and palatines of *Elgaria panamintina* MVZ 191076 in anterior view. (B) Frontal and palatines of *Elgaria multicarinata* TMM 35666 in anterior view. (C) Frontal and palatines of *Elgaria multicarinata* TNHC 4478 in anterior view.

**Fig 18:**
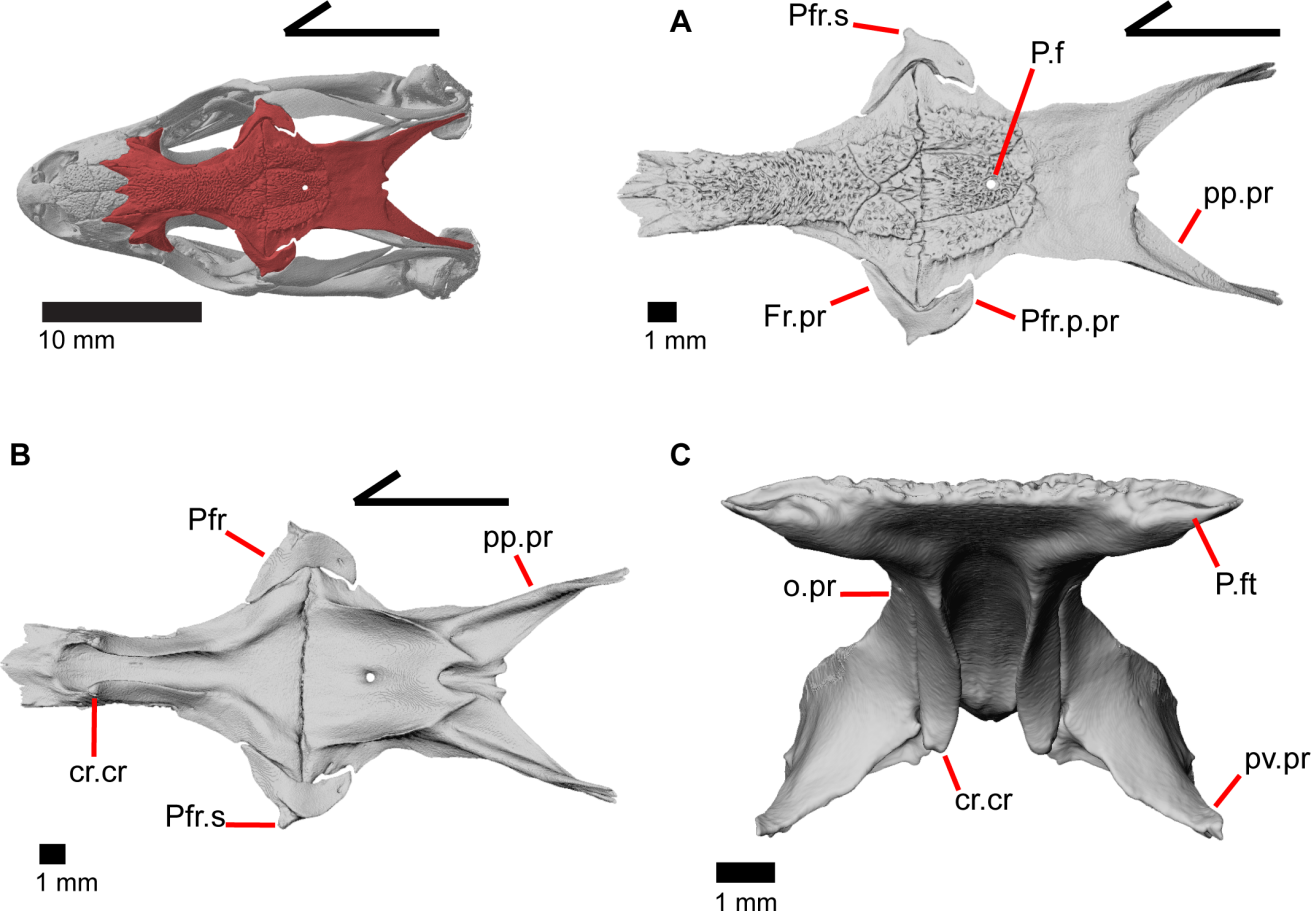
Some roofing bones of *Elgaria panamintina* MVZ 191076. (A) Frontal, postfrontal, and parietal of *Elgaria panamintina* MVZ 191076 in dorsal view. (B) Frontal, postfrontal, and parietal of *Elgaria panamintina* MVZ 191076 in ventral view. (C) Frontal and prefrontals of *Elgaria panamintina* MVZ 191076 in posterior view.

**Fig 19:**
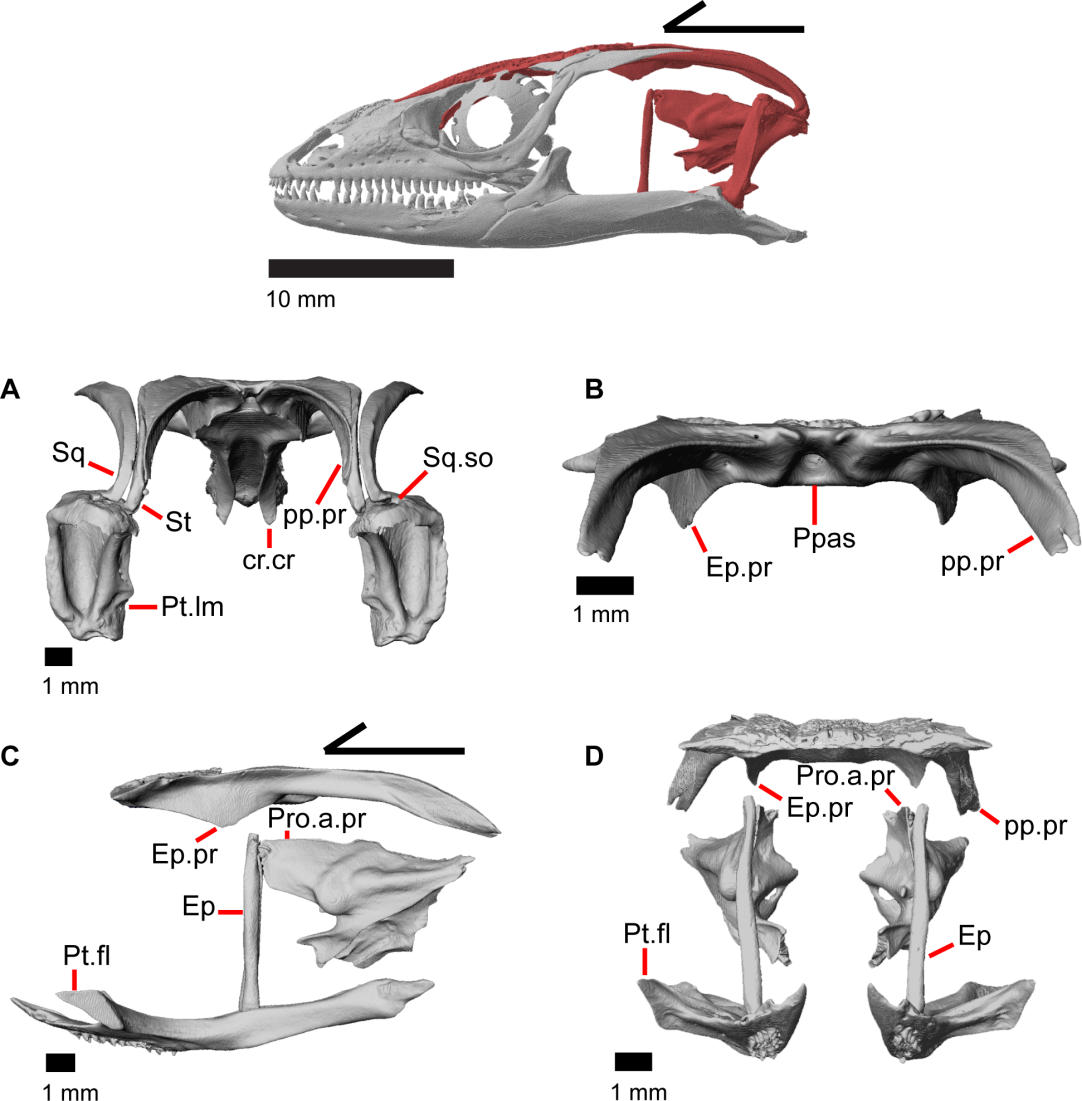
Roofing and posterior cranial elements of *Elgaria panamintina* MVZ 191076. (A) Quadrates and some roofing and temporal bones of *Elgaria panamintina* MVZ 191076 in posterior view. (B) Parietal of *Elgaria panamintina* MVZ 191076 in posterior view. (C) Parietal, epipterygoid, pterygoid, and prootic of *Elgaria panamintina* MVZ 191076 in lateral view. (D) Parietal, epipterygoid, pterygoid, and prootic of *Elgaria panamintina* MVZ 191076 in anterior view.

The frontal is a large bone that provides most of the anterior dorsal roofing of the skull. The paired frontals are fused into a single element, as in all gerrhonotine lizards [5]. However, the frontal does not have an hourglass shape as described by other authors, because constriction around the orbital region is relatively minimal (Fig 16A and 16B). Fusion of the overlying osteoderms to a large portion of the dorsal surface produces a rugose dorsal texture across much of the frontal. The posterior portion is greatly widened and constricted anteriorly so that the interorbital margins of the frontal are relatively straight with the anterior end showing a slight widening. The anterior end has paired dorsal shelves where the posterior portion of the nasals articulate (Figs 16B, 17A and 18A). These facets are separated from each other by the anteromedial process, and are bounded laterally by paired anterolateral processes. Together, the anteromedial process and anterolateral processes form a triradiate anterior end of the bone. The anterior end of the frontal is thickened below the anteromedial process and just anterior to the space for the olfactory tracts. Paired ventral projections, the cristae cranii, form an unclosed canal for the olfactory tracts (Figs 18B, 18C, and 19A) and do not contact the palatine bones below. However, the cristae cranii do extend equal and medial to the level of the apex of the palatines (Fig 17A). Posteriorly the cristae cranii become reduced to less pronounced ridges. Those ridges diverge laterally creating two sides of the boundary of a flat, smooth, triangular surface.

The anterolateral sides of the frontal bear large triangular facets for articulation of the prefrontal (Fig 16B). Those facets are present from the lateral surface of the anteromedial process, ventrally to the lateral surface of the cristae cranii, and posteriorly to where the frontal begins to expand laterally. On the posterolateral surface of the frontal, there is an articulation facet for the anterior half of the postfrontal. The frontoparietal contact is relatively straight, although the suture line does slightly undulate anteroposteriorly. Distinct and posteriorly concave facets for the parietal can be seen on either side of the posterior face of the frontal (Fig 18C). On MVZ 75918, small foramina (anterolateral foramen) are present on the anterolateral processes of the frontal and can be best seen from a dorsolateral view (Fig 16B). These foramina are absent on MVZ 191076.

### Parietal

The parietal is a large bone that forms most of the posterior portion of the skull roof. The main body of the bone is a flattened, trapezoidal table that articulates anteriorly with the frontal and anterolaterally with the postfrontal (Fig 18A and 18B). Variable sculpturing and fusion of osteoderms to the anterodorsal portion of the parietal produces a pitted, sculpted surface. The posterior surface of the table is smooth in both specimens of *Elgaria panamintina* examined. There is an open parietal (pineal) foramen. The dorsal surfaces of the postparietal processes (posterolateral process of Criley [24]) are concave dorsal surface and the ventral surfaces have large crests (Fig 19B). The processes are not parallel-sided with respect to the main body of the parietal, instead curving ventrally and laterally and articulating with the paroccipital processes (Fig 19A; paraoccipital process of Good [3]). In dry skeletal preparations, the paroccipital processes and postparietal processes usually contact, but this is not the case in the CT specimens examined ([43], pers. observation). The entire transverse, flat, anterior surface of the parietal contacts the frontal, while the anteriormost lateral and triangular-shaped edges of the parietal articulate with facets on the frontal. The anterolateral edges of the parietal articulate with the postfrontal (Fig 18A and 18B). Articulation with the postfrontal results in a small or large notch on the lateral edge of the parietal where the posterior tip of the postfrontal articulates. Viewed dorsally, the middle posterior edge of the main body of the parietal between the posterior parietal processes may possess a notch or an unbroken surface. Viewed posteriorly, the pit for the processus ascendens (medial ascendent process of Good [3]) of the supraoccipital is visible and has prominent ridges on both sides (Fig 19B). Longitudinally oriented ventral crests of the parietal form a large but shallow depression. These crests form epipterygoid processes that do not project below the level of the epipterygoid (Fig 19C and 19D).

### Prefrontal

**Fig 20:**
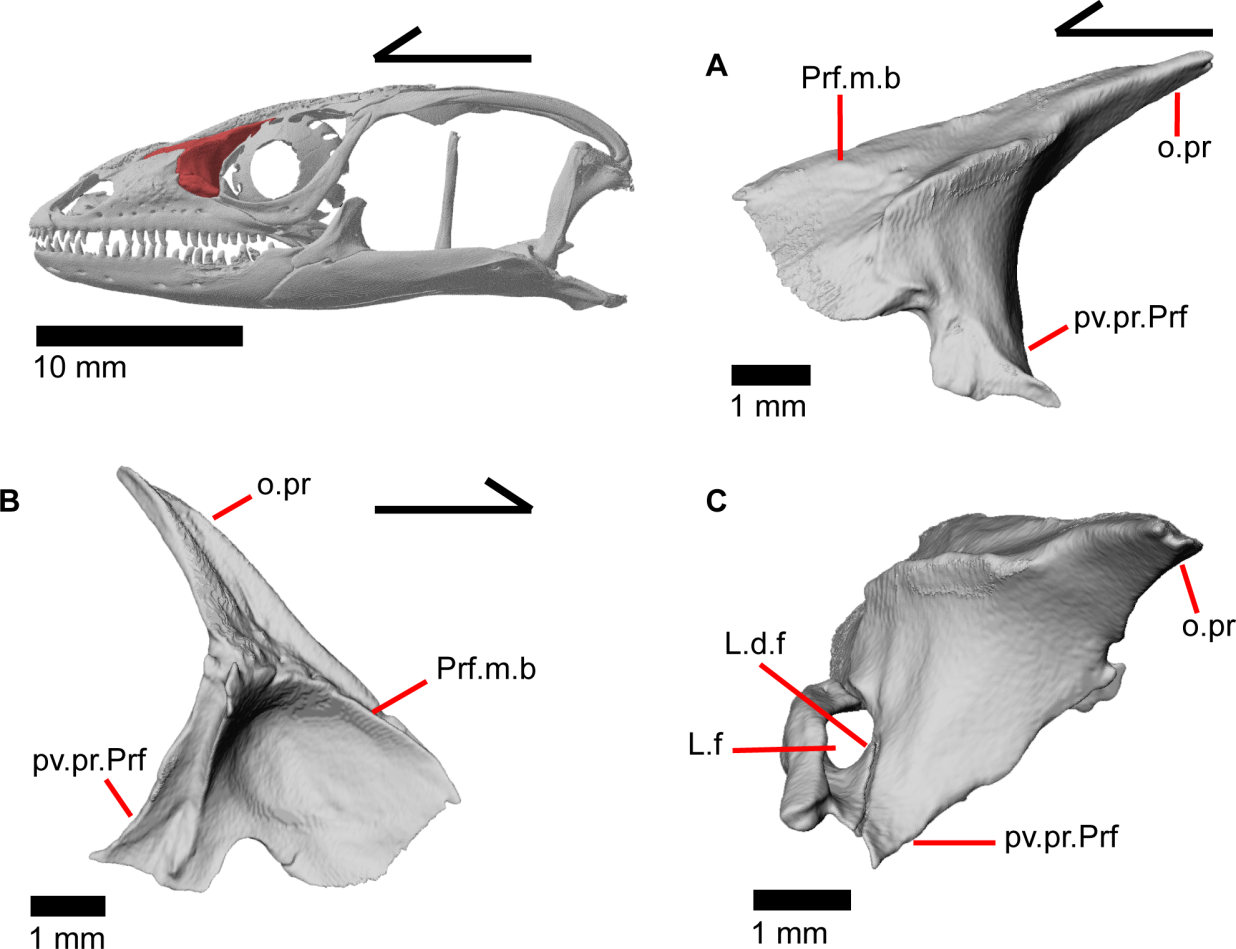
Left prefrontal and lacrimal of *Elgaria panamintina* MVZ 191076. (A) Left prefrontal of *Elgaria panamintina* MVZ 191076 in lateral view. (B) Left prefrontal of *Elgaria panamintina* MVZ 191076 in medial view. (C) Left prefrontal and lacrimal of *Elgaria panamintina* MVZ 191076 in posterior view.

**Fig 21:**
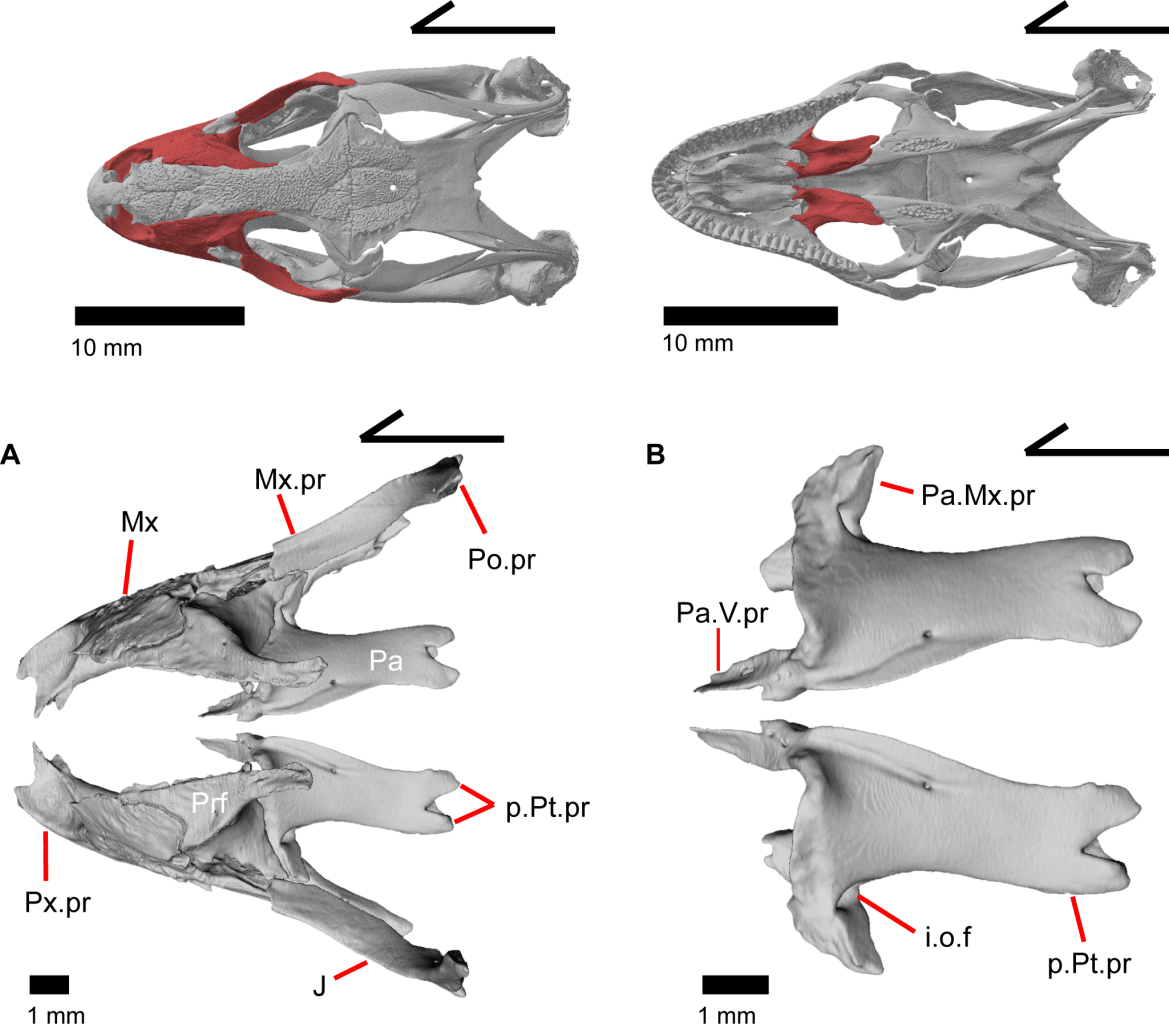
Maxillae, prefrontals, palatines, and jugals of *Elgaria panamintina* MVZ 75918 in dorsal view. (A) Maxillae, prefrontals, palatines, and jugals of *Elgaria panamintina* MVZ 75918. (B) Palatines of *Elgaria panamintina* MVZ 75918 in dorsal view. The reference skull is in ventral view.

The prefrontal (Fig 20A and 20B) contributes to the anterior bordering of the orbit and is a triradiate bone consisting of a main body (pars maxillaris of Criley [24] and anteromedial arm of Good [3]), an orbital process (pars frontalis of Criley [24]), and a posteroventral process (pars jugalis of Criley [24]). The prefrontal contacts the frontal dorsally and medially, the maxilla anteriorly, the jugal posteroventrally, and the palatine ventrally. The main body of the bone is broad and convex with a large anterior articulation facet for the posterior portion of the facial process of the maxilla. When viewed medially, the main body is cavernous (Fig 20B). The long and narrow orbital process articulates with a groove on the anterolateral portion of the frontal. Small medial flanges project medially and articulate with the anterior-most part of the crista cranii of the frontal. A small fenestra is created at this juncture between the prefrontal and the crista cranii (Fig 18C). The posteroventral process overlies the palatine and articulates with the anteromedial surface of the jugal (Fig 21A). There is a medial articulation surface for the palatine, and a posterolateral facet for the jugal. A distinct notch between the main body and the posteroventral process contributes to the margin of the lacrimal foramen (Fig 20C). Laterally, a ridge between the orbital and posteroventral processes provides a space under which the palpebral sits (Fig 14B and 14C).

### Lacrimal

**Fig 22:**
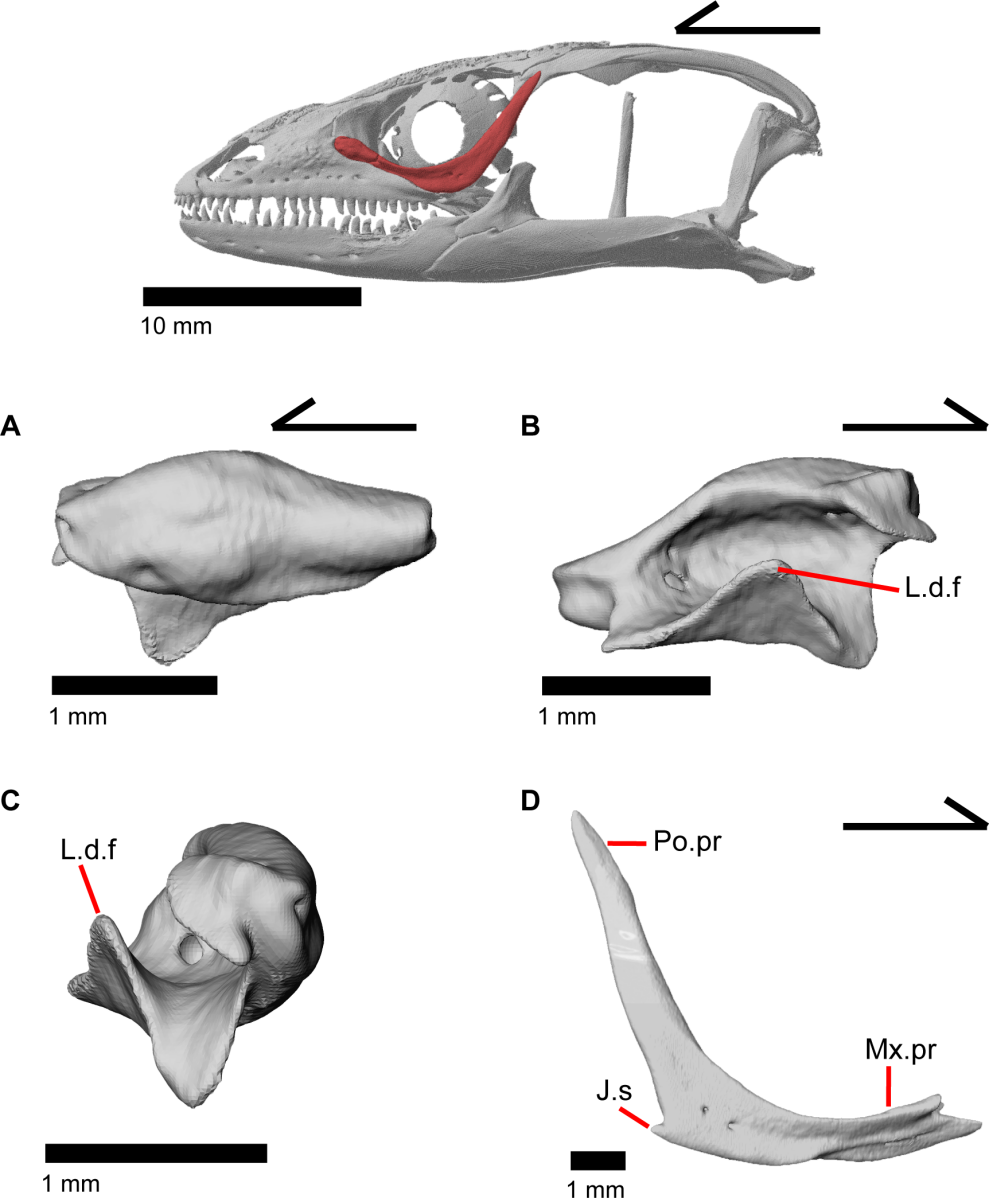
Lacrimal and jugal of *Elgaria panamintina* MVZ 191076. (A) Left lacrimal of *Elgaria panamintina* MVZ 191076 in lateral view. (B) Left lacrimal of *Elgaria panamintina* MVZ 191076 in medial view. (C) Left lacrimal of *Elgaria panamintina* MVZ 191076 in anterior view. (D) Right jugal of *Elgaria panamintina* MVZ 191076 in lateral view.

The lacrimal is a small, irregularly shaped bone. It is ovoid in lateral view but has a distinct posterior projection (Fig 22A and 22B). There is a medial expansion or shelf of the main body of the bone with a dorsally projecting flange (Fig 22C). An unclosed duct, the lacrimal foramen, is visible from anterior or posterior view, and is formed by the dorsal flange rising from the main body of the lacrimal. The lacrimal foramen is bordered by the prefrontal dorsomedially, but most of the border is formed by the lacrimal, contrary to previous reports that the prefrontal forms most of the border in gerrhonotines (Fig 20C) [24]. The lacrimal lies on top of the maxilla just posterior to the facial process of maxilla (Fig 15A and 15B), and the posterior end of the lacrimal fits into an articulation surface on the anterior tip of the jugal. There is a small foramen that opens on the medial surface of the lacrimal, however, there is no distinct path for this foramen and it merges with a complex hollow system within the lacrimal.

### Jugal

The jugal constitutes much of the ventral and posterior border of the orbit. Its medial inflection gives the bone a curved shape (Fig 22D) with a posterodorsally pointing postorbital process (temporal process of Criley [24]) and an anterior maxillary process that terminates at a tip surrounded by the lacrimal, maxilla, and prefrontal (Fig 15B). There is a distinct articulation surface on the anterior tip of the jugal for the lacrimal. The anterior process of the jugal possesses shallow facets that suggest that the jugal is connected to the palatine and prefrontal by a thin layer of connective tissue. The anterior process of the jugal sits on the posterior part of the maxilla and possesses longitudinal ridges and valleys on its ventral surface that correspond with the topography of the dorsal surface of the posterior process of the maxilla. Just posterior to this, the jugal has a medial concavity suggesting connective tissue between the jugal and ectopterygoid. Located near the inflection of the jugal, there is a posteriorly directed spur, present on the right jugal in MVZ 191076 (Figs 15A, 15B and 22D) and both jugals in MVZ 75918. There are one or two foramina near the inflection point on both the lateral and medial surfaces of the jugal. The posterodorsally oriented postorbital process of the jugal runs along a groove in the postorbital and is slightly flattened medially at its end. The postorbital process terminates as a rounded tip.

### Palpebral

The palpebral is a sub-triangular bone that sits in the anterior part of the orbit. It articulates under a small ridge on the lateral surface of the prefrontal and extends anteriorly so that the anterior tip of the palpebral fits into a small depression on the posterior portion of the facial process of the maxilla (Fig 14B and 14C). These bones are often lost in skeletal preparation [3] because they are ripped out in the skinning process or are dislodged and lost by dermestid beetles as they clean the skeleton.

### Postfrontal

The postfrontal is a triradiate bone that articulates with the posterolateral edge of the frontal (Fig 18B). A short lateral spur overlies and articulates with the postorbital and the frontal process of the postfrontal fits into a facet on the frontal. The posterior process is shorter but wider than the frontal process and slots into a triangular space on the parietal thus wrapping around the frontoparietal suture (Fig 18A). The posterior edge of the postfrontal contributes to the anterior border of the supratemporal fenestra. The postfrontal is closely associated with the postorbital, although the two are separate bones as opposed to a single postorbitofrontal bone as is found in some other anguids like *Ophiodes*, some *Anguis*, and *Diploglossus*, as well as anguimorphs like *Varanus* [43]. There is a large dorsal foramen on the posterior process of the postfrontal.

### Postorbital

**Fig 23:**
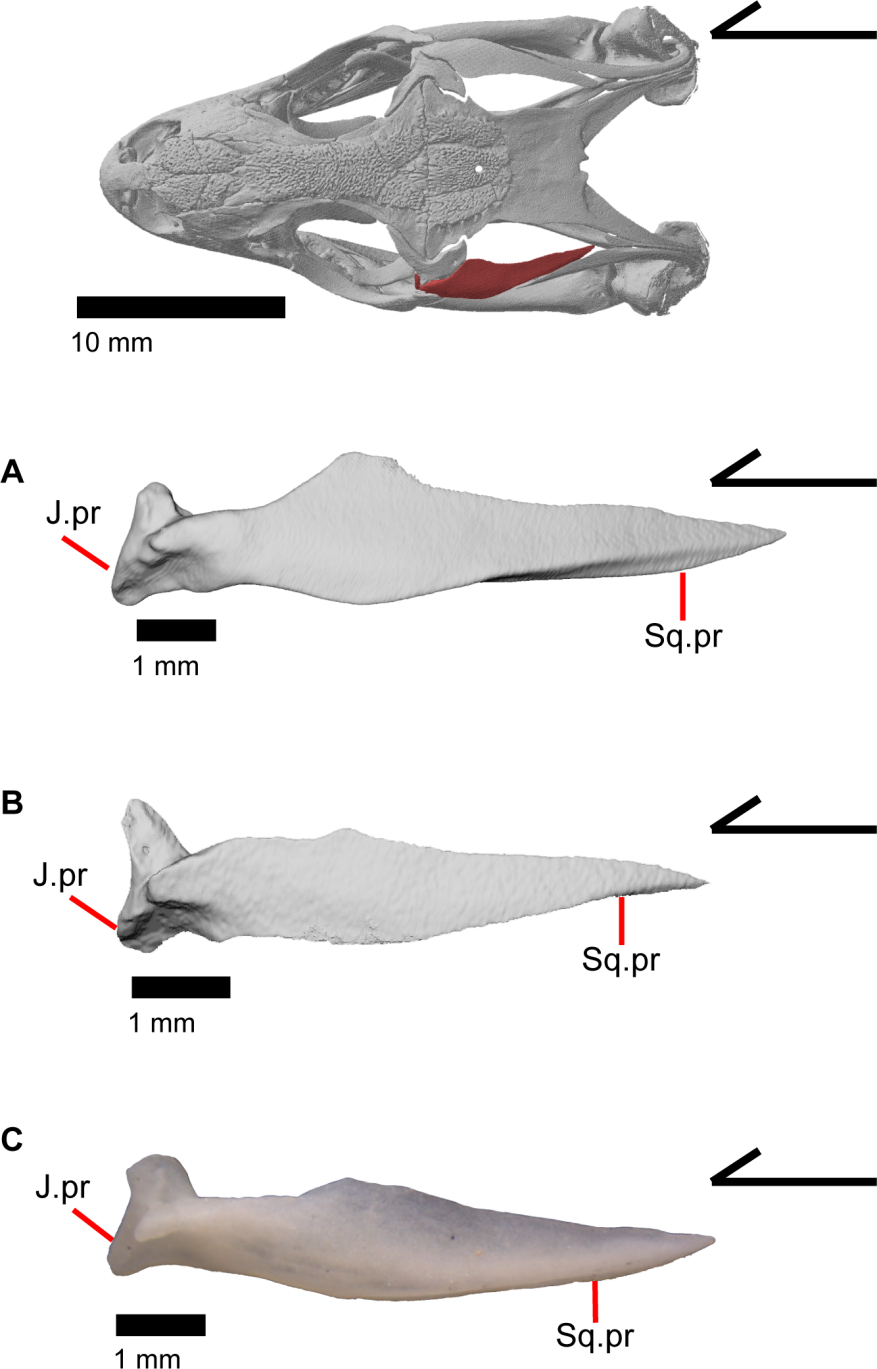
Left postorbitals of *Elgaria*. (A) Postorbital of *Elgaria panamintina* MVZ 191076 from a dorsal view. (B) Postorbital of *Elgaria multicarinata* TNHC 4478 from a dorsal view. (C) Postorbital of *Elgaria multicarinata* TMM M-8987 from a dorsal view.

The postorbital is an elongate, triradiate bone in the temporal region of the skull (Fig 1). Anteriorly, the jugal process of the postorbital points ventrally and the lateral surface articulates with the jugal (Figs 15A, 15B and 23A). Dorsomedial to that, the medially facing postfrontal process articulates ventral to the lateral spur of the postfrontal. These two processes form the anterior head of the bone, which is dorsally widened relative to the portion of bone just posterior. Posterior to that, the bone becomes dorsally widened by a medial shelf. There is a notch between the postfrontal process and the medial shelf where the postfrontal articulates. The posterior half of the bone narrows relative to the medial shelf, and tapers posteriorly. The postorbital forms most of the lateral border of the supratemporal fenestra. The posterior lateral surface of the bone articulates with the squamosal along a broad, shared articulation surface. The posterior squamosal process of the postorbital extends posteriorly more than three quarters of the length of the supratemporal fenestra [2], almost containing the entire lateral border of the fenestra.

### Squamosal

The squamosal is a long, curved, slender bone. The bone is located laterally in the temporal region of the skull and its anterior end runs lateral to the postorbital, gradually tapering to a tip anteriorly (Fig 1). The anterior portion of the squamosal possesses a concave medial articulation surface into which the postorbital articulates. The squamosal has a ventral curve posteriorly, giving the bone an overall bowed form (Fig 2). There is a medial expansion on the squamosal just posterior to the posterior termination of the postorbital. The expansion subsequently becomes much reduced, with the posterior end of the squamosal becoming more tube-shaped as it curves ventrally. The posteromedial portion of the squamosal contacts the supratemporal (Fig 19A). The degree of curvature is greatest at the posterior end so that the termination of the bone is a sub-rounded tip that points anteroventrally instead of posteroventrally (approximately 30 degrees down from horizontal). This posterior tip points into the quadrate and is just dorsal to the squamosal notch of the quadrate. The posterior process of the squamosal is widened relative to the portion of bone anterior and dorsal to the process in MVZ 75918.

### Supratemporal

The supratemporal is a laterally grooved, thin, elongate bone that narrows anteriorly. The posteroventral portion of the supratemporal expands laterally to meet the cephalic condyle of the quadrate (Fig 19A). The ventromedial surface of the posterior end articulates with the paroccipital process. The supratemporal is located lateral to the postparietal process, where it extends almost the entire length of the process. The anteriormost portion of the supratemporal curves medially with the postparietal process and wraps around it tightly in MVZ 75981, but in MVZ 191076 it extends forward without wrapping around the postparietal process. The supratemporal is medial to the squamosal and contacts the squamosal for most of its length. The anterior tip of the supratemporal lies just posterior to the posteriormost tip of the postorbital in MVZ 191076 but extends anterior to the tip of the postorbital in MVZ 75918. The anterior extent of the supratemporal is a function of ontogeny [43], suggesting that MVZ 191076 may represent a younger individual than MVZ 75918.

### Quadrate

**Fig 24:**
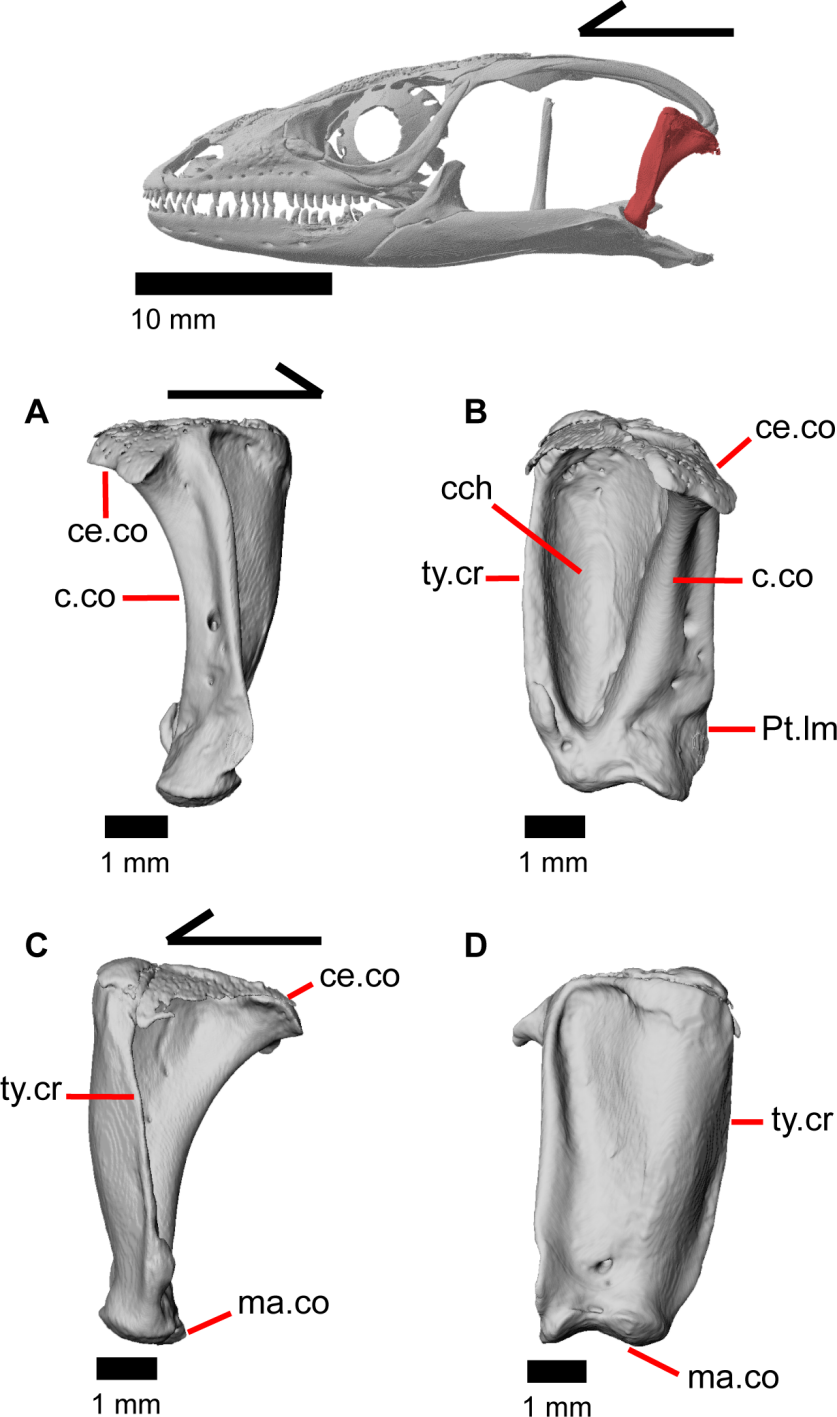
Left quadrate of *Elgaria panamintina* MVZ 75918. (A) Medial view. (B) Posterior view. (C) Lateral view. (D) Anterior view.

The quadrate is a large and robust bone that participates in the cranio-mandibular joint. It contacts the supratemporal dorsomedially just above the cephalic condyle, and the squamosal just lateral to that (Fig 19A). The quadrate slots ventrally into the articular surface of the articular (Fig 2). The mandibular condyle is convex (Fig 3A) and narrows ventrally (Fig 24A–24D). The quadrate possesses a lower medial pterygoid lamina that articulates with the quadrate process of the pterygoid. The cephalic condyle slants posteroventrally and meets the paroccipital process medially. The conch of the quadrate is deep. The dorsal face of the quadrate is cartilaginous to varying degrees. In both specimens, the conch deepens due to the ossification of cartilage around the cephalic condyle and the tympanic crest, creating an expanded lip. This ossification also results in the “squamosal notch” being enclosed as a large foramen formed by the cephalic condyle and posterior cartilage ossification. Thus, the articulation of the quadrate with the squamosal is a peg-and-socket (squamosal socket of the quadrate), and that discrepancy potentially varies with ontogeny [43]. The ventral portion of the tympanic crest is bulbous in MVZ 191076, but is relatively straight with a slight lower expansion in MVZ 75918. Lateral folding of the tympanic crest creates a wavy appearance. Two or three foramina penetrate laterally through the quadrate central column (Fig 24A; posterior crest of Criley [24]) and are in variable positions on the medial and lateral face of the column.

### Pterygoid

The pterygoid is an elongate triradiate bone located in the palatal region of the skull (Fig 3A and 3B). The anterior end is characterized by a tapering, flattened palatal plate (medial palatine process of Criley [24]) possessing two anterior facets for articulation with the posterior palatine projections (Fig 10B). The more medial facet is located on the dorsal surface and is defined by ridges on either side, while the other facet is a concavity on the anterolateral edge. MVZ 75918 possesses a defined facet for the overlying posterodorsal projection of the ectopterygoid, however, it is not distinct on MVZ 191076. The anterolaterally projecting pterygoid flange (Fig 10A; lateral transverse process of Criley [24] and transverse process of Good [3]) has a flat, triangular lateral surface that bends to fit into the lateral facet on the ectopterygoid. There is a ridge on the dorsolateral surface of the pterygoid that runs from the pterygoid flange to just anterior of the fossa columella (fossa pterygoidei of Criley [24]). The fossa columella is a fairly deep rounded socket on the dorsal surface approximately midway along the length of the pterygoid. The base of the epipterygoid articulates inside the fossa columella (Fig 19C and 19D). The quadrate process of the pterygoid extends posteriorly behind the fossa columella and articulates with the quadrate (Fig 3A and 3B). Just posterior to the fossa columella on the quadrate process, there is a groove (postepipterygoid groove) that runs a short while before shallowing and disappearing. A lateral ridge is present just posterior to the end of the channel where the lateral surface of the pterygoid articulates with the quadrate. The quadrate process of the pterygoid terminates as a rounded tip and the posteromedial surface of the process is concave. The anteromedial surface of the quadrate process of the pterygoid also possesses a shallower medial concavity where the basipterygoid process of the sphenoid articulates ventromedially (Fig 10C). The broad palatal plate of the pterygoid possesses a foramen just medial to the edge of the dorsolateral ridge, but may also possess small and variably placed accessory foramina. Those foramina, along with a foramen in the anterior end of the posteromedial concavity of the quadrate process and a foramen just posterior to the fossa columella are all connected through a longitudinal canal running through the bone.

There is variation in the number of pterygoid teeth on the ventral surface. MVZ 75918 has some sculpturing and no teeth (Fig 10A), while MVZ 191076 has 3 rows of 15–22 pterygoid teeth similar to other *Elgaria* [3]. The mesial teeth are slightly recurved (Fig 19C) and the distal teeth are not recurved.

### Epipterygoid

**Fig 25:**
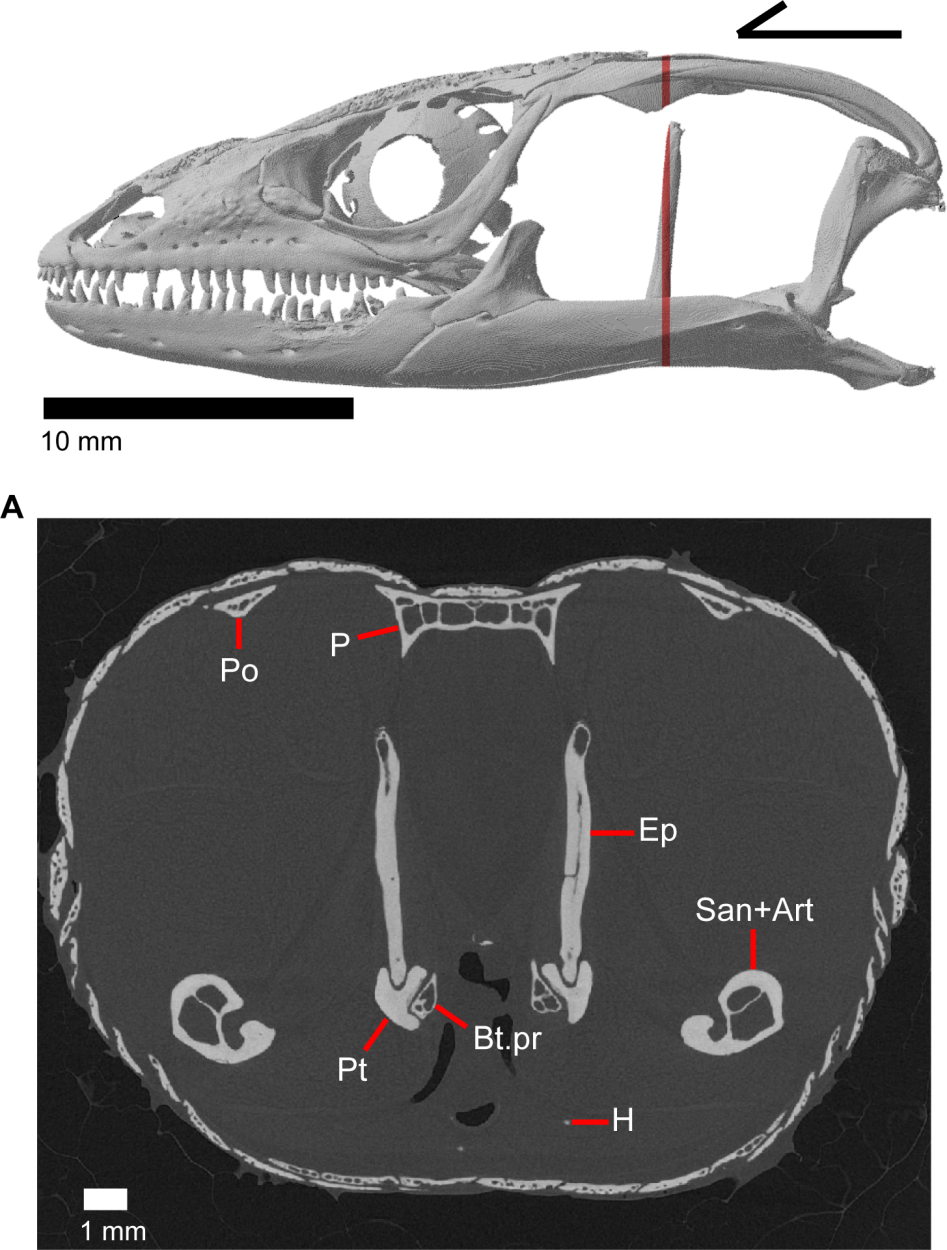
CT coronal slice of *Elgaria panamintina* MVZ 191076.

The epipterygoid is a long, thin, columnar bone that is relatively straight and maintains an approximately constant diameter. The bone stands straight with the ventral end resting in the fossa columella of the pterygoid (Fig 19C and 19D). The dorsal portion of the epipterygoid rises to articulate anterolaterally with the alar process of the prootic. The epipterygoid does not extend far enough dorsally to contact the descending epipterygoid processes of the parietal, and there is substantial space between the epipterygoid and parietal. When CT slices are viewed in cross-section, the epipterygoid can be seen to be hollow with a long tube that runs the length of the bone (Fig 25A). This tube opens on the dorsal and ventral ends of the bone and sometimes along the bone shaft, resulting in irregularly placed foramina.

### Ectopterygoid

**Fig 26:**
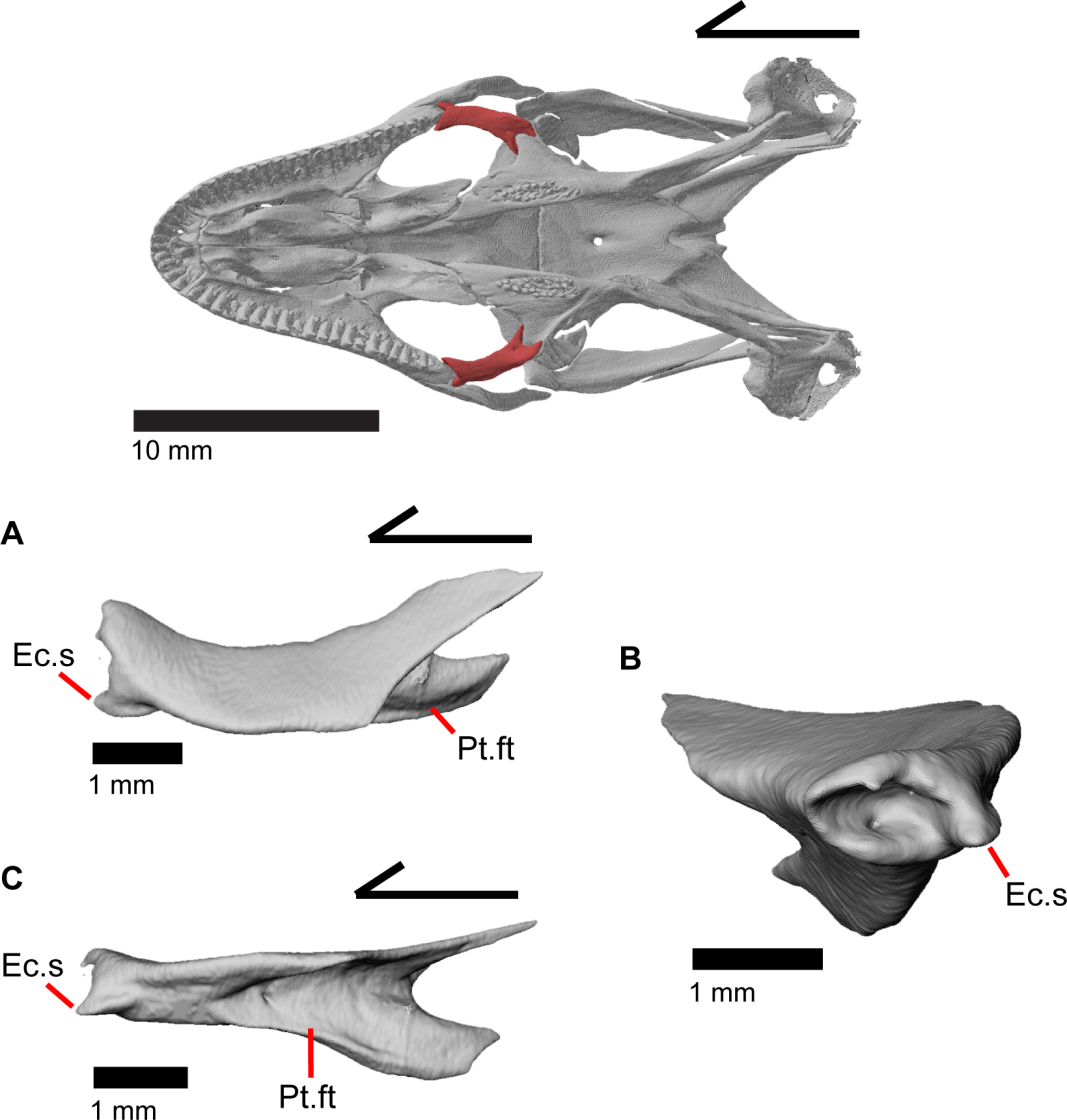
Left ectopterygoid of *Elgaria panamintina* MVZ 191076. (A) Dorsal view. (B) Anterior view. (C) Lateral view.

The ectopterygoid is curved medially at both ends and has a relatively flat dorsal surface that slants ventromedially (Fig 26A–26C). The anterior end of the ectopterygoid has a deep socket where the posterior process of the maxilla articulates. On the lateral side of the socket, there is a distinct spur which articulates ventromedially with the jugal. This spur is defined from a ventral view by a small notch (Fig 10C), but is notably less visible dorsally [3]. The posterolateral surface of the ectopterygoid is highly excavated forming a facet for articulation with the pterygoid flange. The posterior end of the ectopterygoid is bifurcated with two projections that overlap the pterygoid flange. One projection articulates dorsal to the pterygoid flange and the other articulates ventrally, thus forming a secure slot-like articulation between the ectopterygoid and pterygoid. Viewed ventrally, the posterior end of the ectopterygoid has a ‘u’ like appearance in articulation with the pterygoid [3]. Within the facet for articulation for the pterygoid, there are one to three foramina. Those foramina pierce ventrally through the bone and have a single opening on the posteroventral surface of the ectopterygoid (Fig 10C). There may also be a small foramen inside the socket where the maxilla articulates as observed on the left ectopterygoid of MVZ 191076 (Fig 26B).

### Septomaxilla

**Fig 27:**
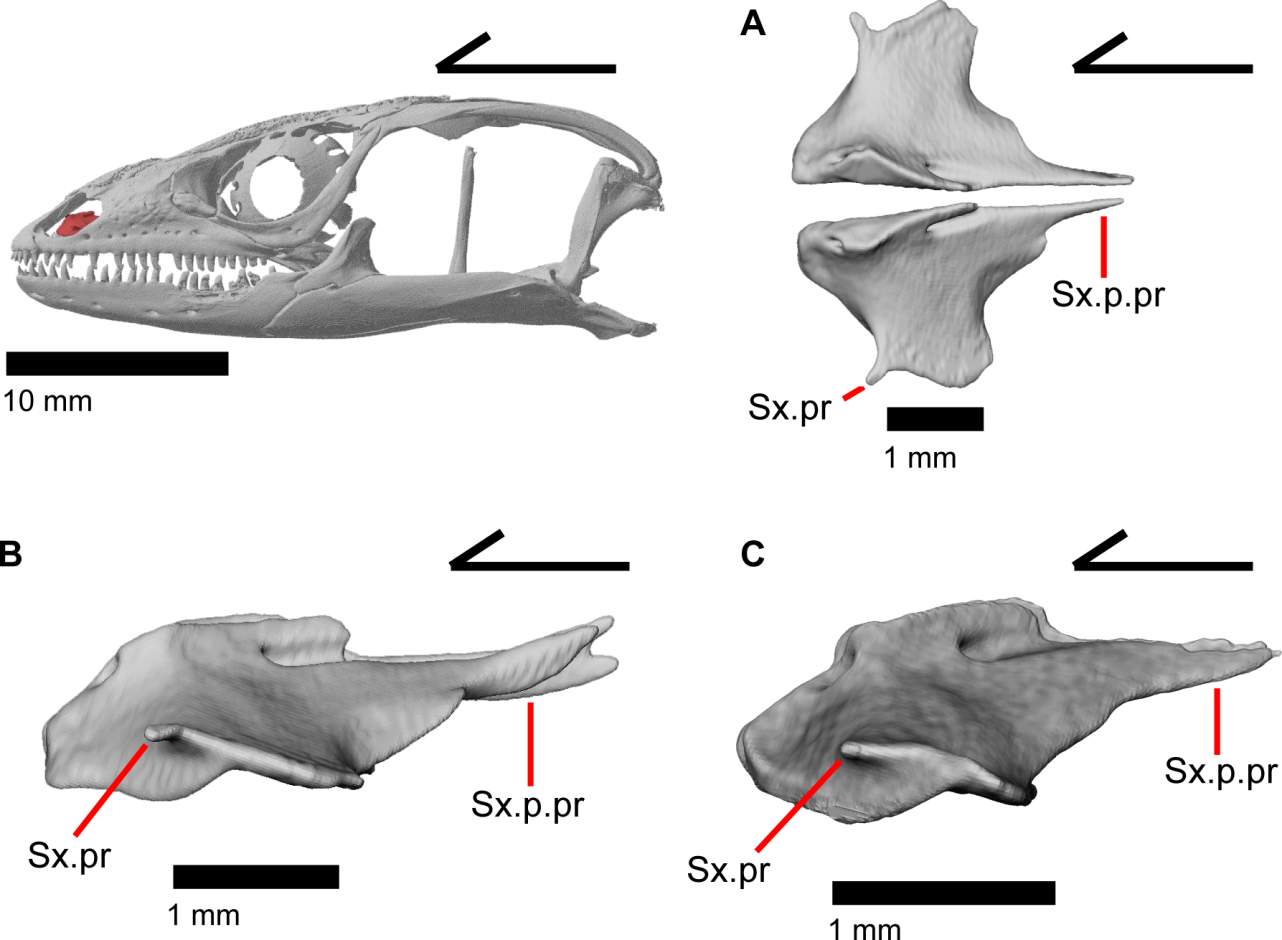
Septomaxillae of *Elgaria*. (A) Septomaxillae of *Elgaria panamintina* MVZ 191076 from a dorsal view. (B) Septomaxillae of *Elgaria panamintina* MVZ 191076 from a lateral view. (C) Septomaxillae of *Elgaria multicarinata* TNHC 4478 in lateral view.

The septomaxilla is a small paired bone located just dorsal to the vomeronasal region of the vomer. It has a concave ventral surface. The two septomaxillae are separated by a small amount of space that slightly increases anteriorly (Fig 27A). The anterior end of the septomaxilla is located just posterior to the maxillary lappet; the posterior end terminates as a long, thin process which is bifurcated on the left septomaxilla in MVZ 191076 (Fig 27B). The posterior process (septal process of Criley [24]) is slightly curved dorsally at the posterior end [3]. The dorsal surface of the septomaxilla is defined by a tall medial ridge with a foramen (canal for the medial branch of the medial ethmoid nerve in Criley [24]) that penetrates the element anteroposteriorly. This ridge decreases in height laterally to form a flattened surface. This flat surface has a lobed appearance except for a small anterolateral projection, which may be homologous to a structure seen in other anguids [3]. The anterolateral projection as well as the lateral portion of the flattened surface lie above the maxilla, just inside the naris.

### Vomer

**Fig 28:**
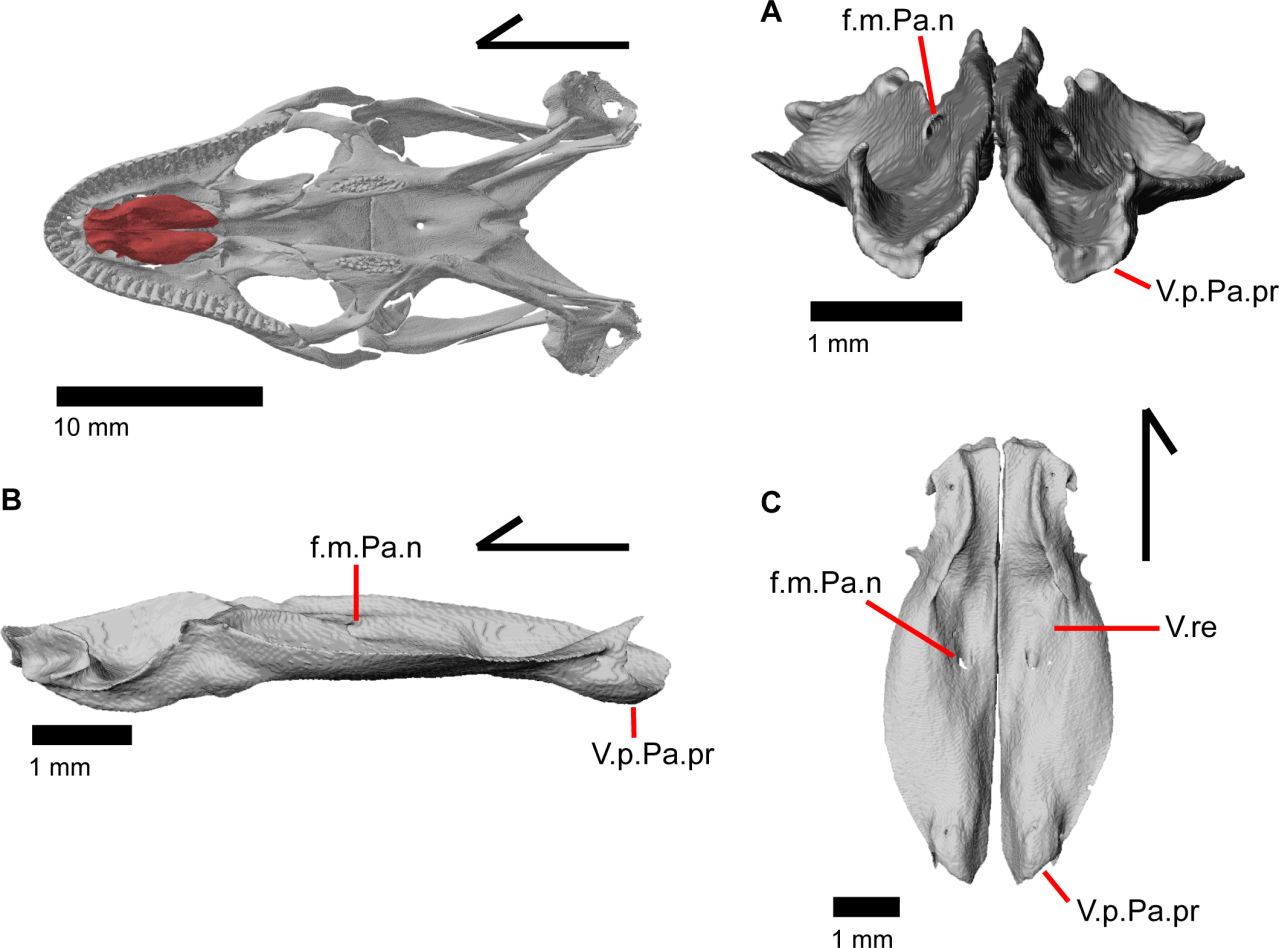
Vomers of *Elgaria panamintina*. (A) Vomers of *Elgaria panamintina* MVZ 75918 in posterior view. (B) Vomers of *Elgaria panamintina* MVZ 191076 in lateral view. (C) Vomers of *Elgaria panamintina* MVZ 191076 in ventral view.

The vomer (prevomer of Criley 1968 [24]) is a paired midline bone within the anterior palatal region of the skull (Fig 3A and 3B). It meets the contralateral element in a flattened medial wall which is steeply sloped laterally (Fig 28A). The vomer diverges from its contralateral element at its posterior end. The anterior surface of the vomer has cupped concave facets that clasp the palatal process of the premaxilla along with a deeper, slightly lateral facet for articulation with the maxillary shelf (Fig 28B). A part of the anterior portion of the vomer is constricted in width when viewed in dorsal and ventral view (Fig 28C). The complex dorsal surface of the vomer has two main concavities, one posteriorly in the nasal region and one anteriorly in the vomeronasal region. These concavities are separated from one another by an anterolateral ridge that runs posteriorly to meet the tall median wall of the vomer and anteriorly to become a small flange which extends into the choana (and is slightly bifurcated on the left vomer in MVZ 191076; Fig 3B). These flanges barely extend far enough laterally to overlap the maxilla and separate the vomeronasal opening from the choana in palatal view. On the left vomer of MVZ 191076, the lateral flange is not well developed and fails to overlap the maxilla and separate the vomeronasal opening from the choana (Fig 3A and 3B).

The dorsal surface of the posterior palatine process of the vomer has a concave facet that accommodates the anterior vomerine process of the palatine. The facet for the vomerine process of the palatine is bordered laterally by a ridge that extends posterodorsally. This ridge becomes a projection or flange that articulates with the anterior vomerine process of the palatine (Fig 28B). The ventral surface of the vomer has a curved lateral ridge which extends posteriorly less than half the length of the vomer and which borders a medial concavity (Fig 28C). The medial concavity of the vomer along with the concavity of its contralateral element form a large central recess for the median palatal sinus [43]. On the posteroventral surface of the vomer, there is a slight bulging (Fig 28B; descending tubercle or ridge of Gauthier et al. [11]) ventral to the articulation with the palatine. Approximately midway along the length of the vomer, there is an inclined piercing foramen for the medial palatine nerve [3]. This foramen empties ventrally into the central recess. Additionally, there is a foramen on the right vomer of MVZ 75918 and MVZ 191076 that opens dorsally within the vomeronasal concavity and empties ventrally at the anterolateral margin of the central recess.

### Palatine

The palatine is a paired element located in the palatal region of the skull just posterior to the vomer. The palatines do not meet one another (Fig 21B), although they are reported to meet in other species of *Elgaria* [43]. The palatine possesses an anterior vomerine process (prevomerine process of Criley [24]) that articulates with a facet on the posterior end of the vomer. The vomerine process of the palatine has small accessory lateral and medial projections that vary in size and distinctiveness. The vomerine process gradually inclines posteriorly and connects with the main body of the bone. The palatine has a deep rounded choanal groove that gives the bone a cave-like appearance from an anterior view (Fig 17A). Part of the lateral wall of the choanal groove is curved medially, below which is a facet for articulation with the widest portion of the shelf of the maxilla. The facet becomes less distinct laterally and ventral to the lateral maxillary process of the palatine which lies on top of the maxillary shelf (Fig 10B). The end of the maxillary process terminates in a posteriorly facing tip and articulates with a small depression on the medial surface of the jugal. A large infraorbital foramen is located on the anterior floor of the orbit and runs anteroposteriorly through the maxillary process of the palatine. The anterior opening of this foramen empties onto the maxillary shelf. The apex of the palatine is the anterior roof of the choanal groove. However, it does not contact the crista cranii of the frontal. The dorsal surface of the palatine slants down posteriorly and laterally from the apex. Where the palatine slopes downwards laterally, the prefrontal articulates dorsally in a slight depression on the dorsal surface of the palatine. The dorsal surface of the palatine also possesses an upturned medial edge. The posterior process of the palatine (pterygoid process of Good [3]) is bifurcated, with a medial projection that articulates with a facet on the anterior palatal plate of the pterygoid and a lateral projection that articulates with a small notch on the pterygoid. These two projections are nearly equal in their posterior extension, but vary among individuals in shape by being either blunt or pointed. These posterior projections create the tongue-in-groove articulation for the transverse palatine/pterygoid joint [43]. There are no teeth on the palatine.

The anterior vomerine process of the palatine has multiple foramina that open along its length (Fig 17A). These foramina are connected by a larger canal and include, a foramen that opens medially, one or two foramina that open dorsally, and a foramen that opens on the medial surface of the choanal groove. Along the upturned medial ridge of the palatine, there are openings for one or two foramina (Fig 21B) which are connected to two foramina on the ventromedial surface of the palatine and up to five foramina which open on the ventral surface of the palatine.

### Scleral ossicles

Scleral ossicles are small bones within the sclera of the eye that develop to form a ring (Figs 1, 2 and 4). The number of bony plates forming the ring and the pattern of overlap between the plates was hypothesized to be phylogenetically informative in other taxa [48]. We counted the overall number and pattern of overlap of the scleral ossicles of specimens of *Elgaria panamintina* using the methodology of Gugg and Underwood [49, 50]. Three observers (SS, DTL, CJB) independently counted the number and pattern of the scleral ossicles in both specimens of *Elgaria panamintina*. We all recovered a scleral ossicle formula of 1+, 4-, 6+, 7-, 8+, 10-, with 14 total scleral ossicles in the eye. This formula matched previous accounts for Gerrhonotinae, although this pattern occurs in other clades of lizards including some members of Iguanidae, Teiidae, Xenosauridae, and Scincidae [50]. An increased sample size may lead to an increase in the amount of variation in the formula, as observed in other lizard clades [51].

### Orbitosphenoid

**Fig 29:**
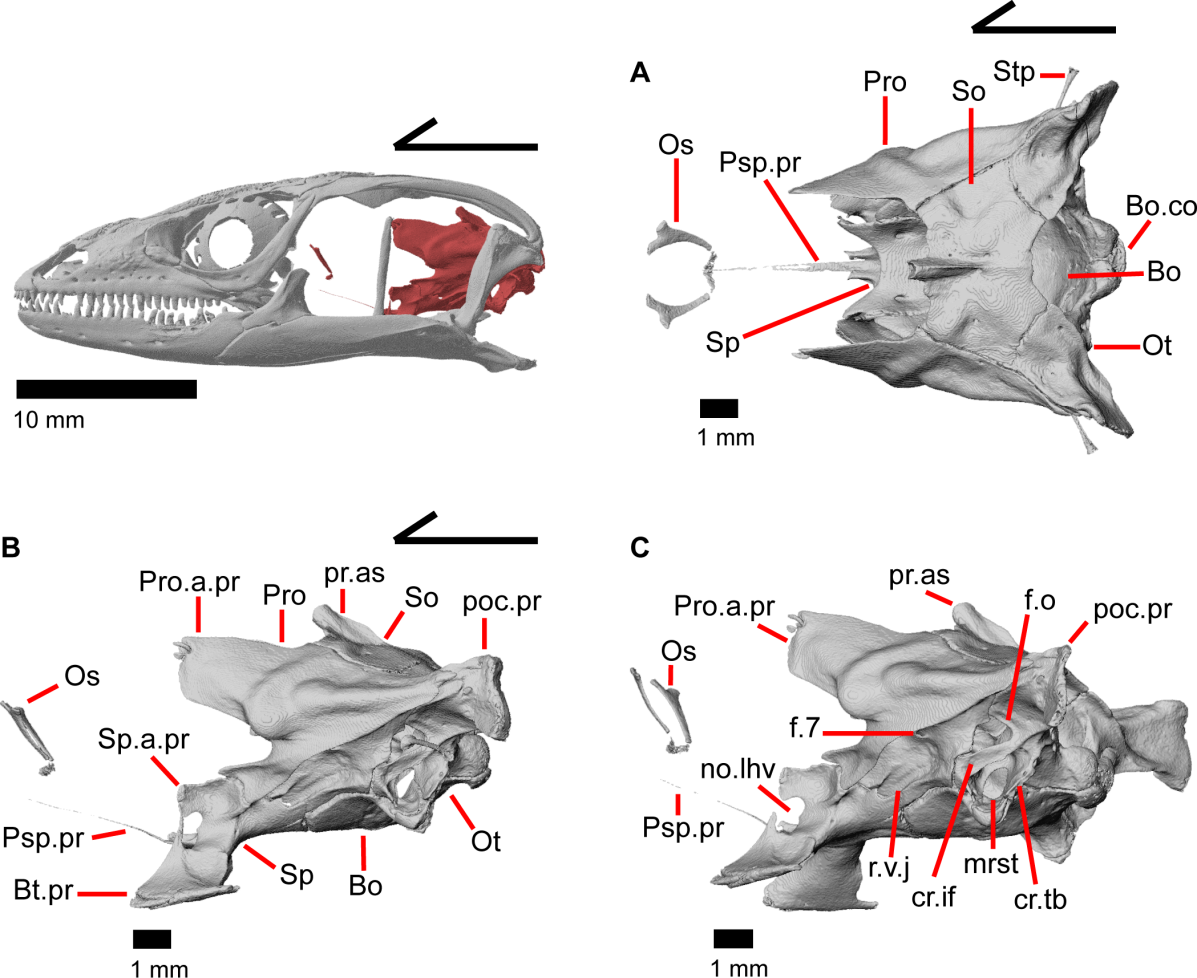
Osseous braincase elements of *Elgaria panamintina* MVZ 191076. (A) Dorsal view. (B) Lateral view. (C) Posterolateral view.

**Fig 30:**
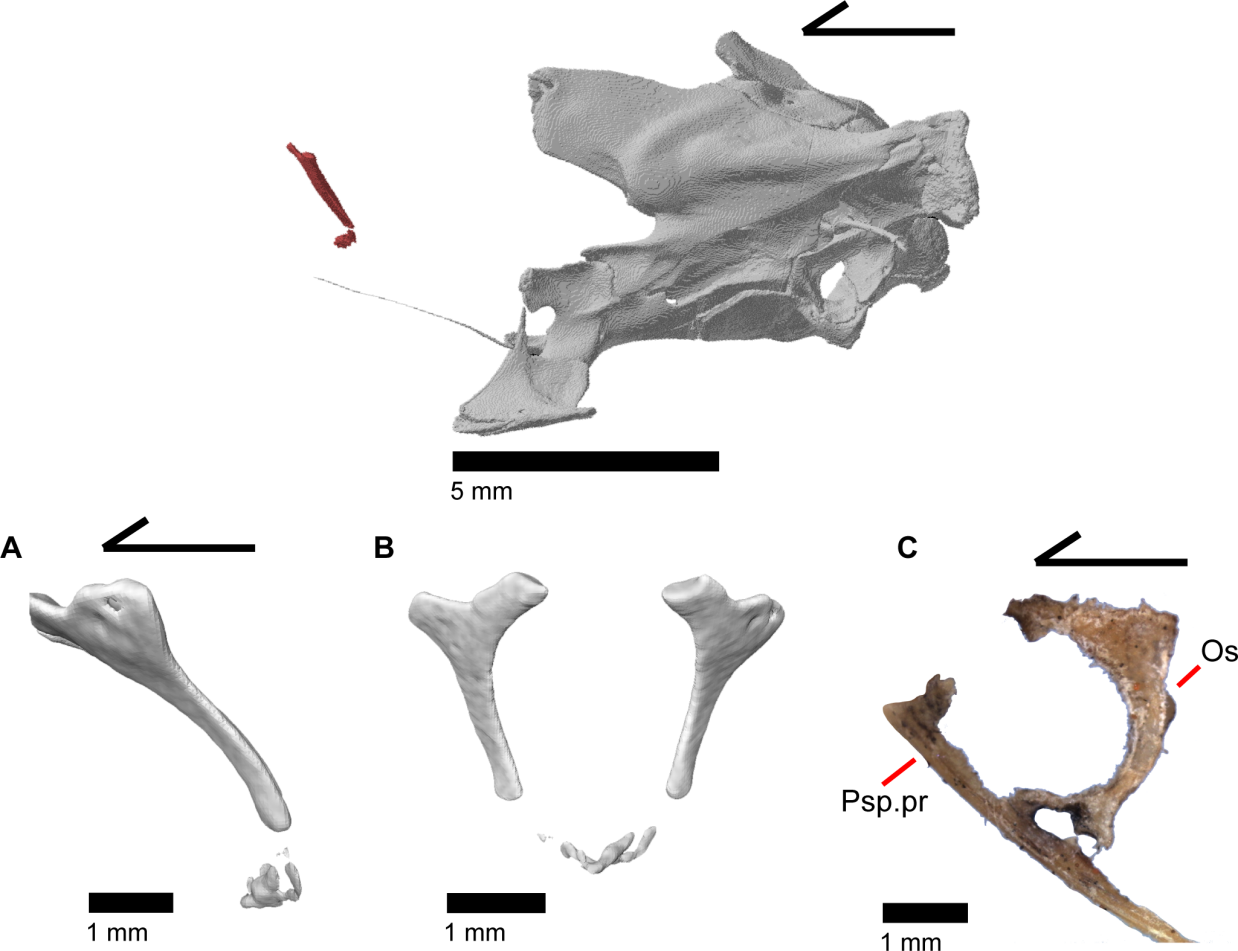
Orbitosphenoids of *Elgaria*. (A) Orbitosphenoids of *Elgaria panamintina* MVZ 75918 in lateral view. (B) Orbitosphenoids of *Elgaria panamintina* MVZ 75918 in anterior view. (C) Orbitosphenoid and parasphenoid process of *Elgaria multicarinata* TMM M-8975 in lateral view.

The orbitosphenoid is a small paired bone that lacks contact with other ossified cranial elements. The orbitosphenoid ossifies from the pila metoptica and forms the orbitotemporal portion of the osseous braincase (Fig 29A–29C) [52]. The orbitosphenoid ossifies prior to hatching in other *Elgaria*, such as *Elgaria coerulea* [47]. In lateral view, the orbitosphenoid is located nearly equidistant from the palatine as from the pterygoid and lies medial to the posterior margin of the orbit (Fig 2). The orbitosphenoid is dorsal to the parasphenoid process (parasphenoid rostrum of Evans [43]) of the sphenoid and lies anterior to the epipterygoids. The orbitosphenoid is inclined anteriorly and is connected to the contralateral element by calcified cartilage. The orbitosphenoid ossifies as a costiform bone that is dorsoventrally flattened, anteroposteriorly elongate, and with ends that curve medially (Fig 30A and 30B). Increased ossification of the orbitosphenoid resulting in changes in shape through ontogeny has been recorded in iguanine lizards [53]. However, in one growth series of *Elgaria coerulea* the orbitosphenoid fully developed and reached “full proportions” before birth [47]. The orbitosphenoid is often lost in preparation of skeletal specimens due to the lack of bony contacts with other elements [43].

### General features of the braincase

The braincase of *Elgaria panamintina* is a complex structure that includes the orbitotemporal portion of the osseous braincase and the posterior portion of the skull including the midline and anteroventral sphenoid (Fig 29A–29C), which represents the fusion between the dermatocranial parasphenoid and the chondrocranial basisphenoid [52]. These elements fuse to form the sphenoid prenatally in other *Elgaria* [47]. Additional midline bones on the braincase include the basioccipital and the supraoccipital. Paired elements on the braincase are the anterolaterally located prootic and the posterior/posterolaterally located otooccipital. The otooccipital represents a fusion between the exoccipital and the opisthotic elements that fuse prenatally in most other squamates [52]. The ossified orbitotemporal region of the braincase includes the paired orbitosphenoid which reaches full proportions prenatally in other *Elgaria* [47]. Postnatally, fusion continues between braincase elements, including the closing of the basicranial fenestra, an opening between the sphenoid and basioccipital [47, 54]. In the closely related species *Elgaria multicarinata*, significant shape change was documented as a function of ontogeny in the prootic and the otooccipital [55].

We note that although we use the terms ‘articulation’ and ‘contact,’ in the CT data there is often a small space between the bones that is suggestive of a soft tissue connection between the bones. The braincase of *Elgaria panamintina* articulates with several cranial elements. Anteriorly the braincase contacts the pterygoids via the basipterygoid processes of the sphenoid and the epipterygoid through the alar process of the prootic. The braincase also contacts the quadrate, supratemporal, and parietal through the paroccipital process of the otooccipital. Although the braincase is known to be connected to the parietal through the ascending process of the tectum synoticum of the supraoccipital [56], a lack of ossification of this structure prevents a visible contact in the CT data.

### Supraoccipital

**Fig 31:**
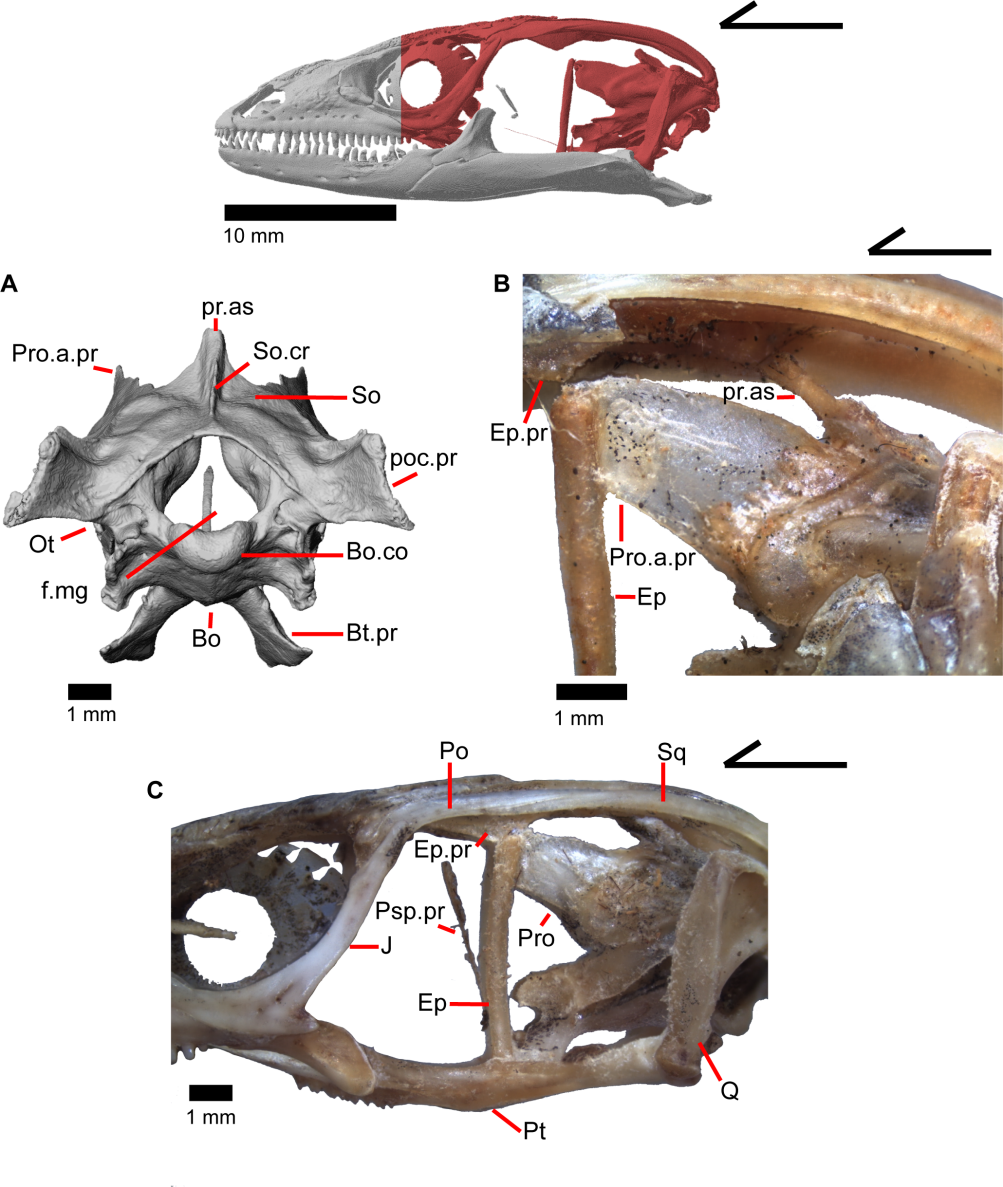
Osseous braincase and posterior cranial elements of *Elgaria*. (A) Osseous braincase elements of *Elgaria panamintina* MVZ 75918 in posterior view. (B) Epipterygoid and some roofing and braincase bones of *Elgaria multicarinata* TMM M-8975 from a lateral view. (C) Posterior skull region of *Elgaria multicarinata* TMM M-9005 in lateral view.

**Fig 32:**
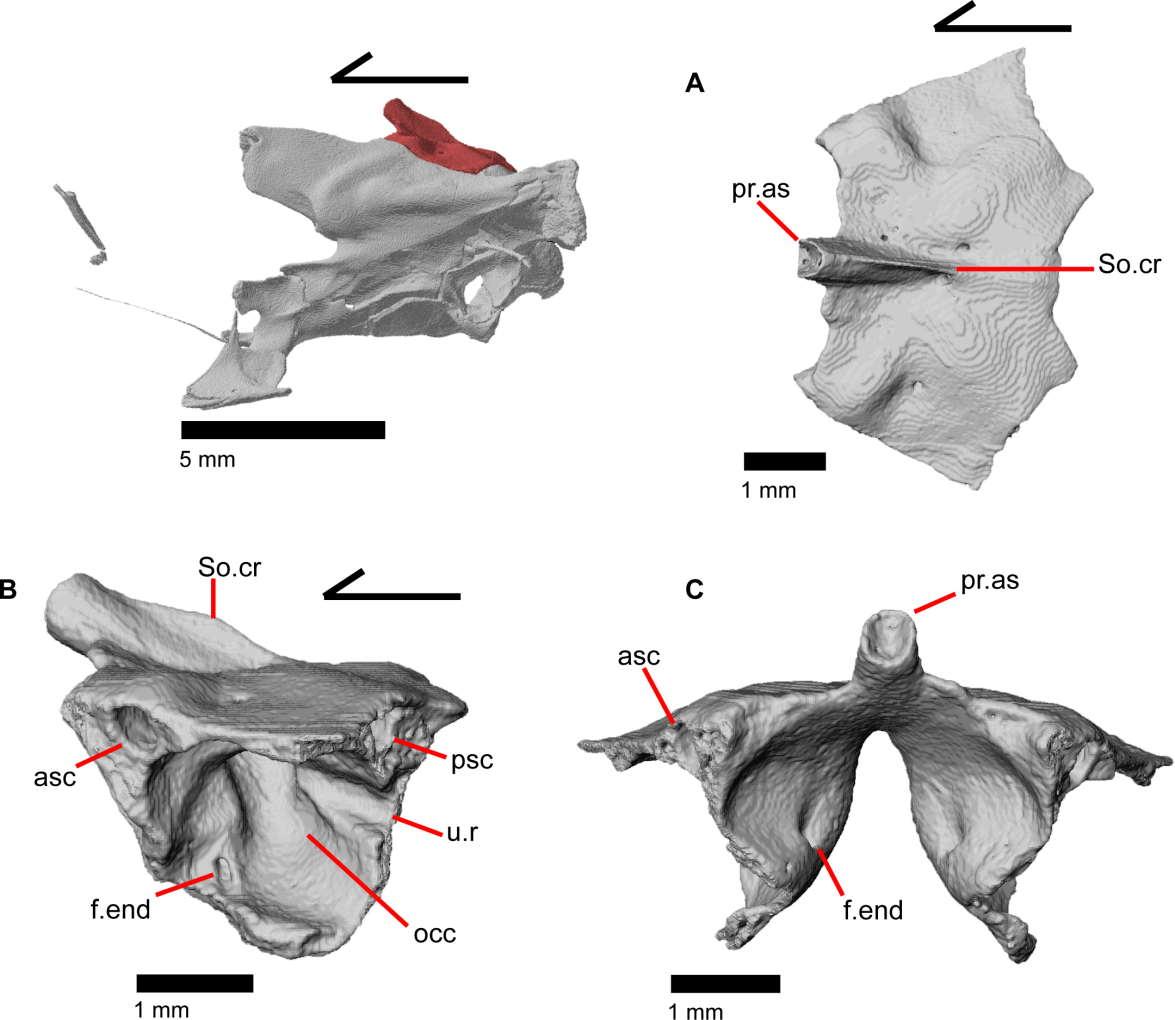
Supraoccipital of *Elgaria panamintina* MVZ 191076. (A) Dorsal view. (B) Lateral view. (C) Anterior view.

The supraoccipital is the dorsal roofing bone of the braincase, and forms the dorsal margin of the foramen magnum (Fig 31A). The supraoccipital contacts the otooccipital posteriorly and ventrally. The dorsal portion of that contact extends anterolaterally to the point where the supraoccipital, otooccipital, and prootic meet. Where they meet, a triangular flange of the supraoccipital overlies the otooccipital. The supraoccipital contacts the prootic anterolaterally and ventrally. When viewed dorsally, the supraoccipital is wider than it is long (Fig 32A). On the dorsal surface, there are depressions on the lateral margins of the element.

The ascending process (processus ascendens) does not contact the parietal (even weakly). Observations of dry skeletal specimens of *Elgaria multicarinata* show contact between the paretial and the ascending process [43]. When viewed laterally, the base of the processus ascendens does project anteriorly from the main body of the element (Fig 32B). The ascending process of the tectum synoticum appears cartilaginous, and is fragmentary and discontinuous with the base of the processus ascendens. The distal portions of the processus are variably calcified cartilage, which in dry skeletal specimens of *Elgaria multicarinata* yield a continuous structure extending anterodorsally to contact the parietal along an elongated recess on the ventral surface of the latter element (Fig 31B). The process makes a 30–45 degree angle with the main body of the element. There is a thin midline crest (sagittal crest of McDowell and Bogert [57]) running down the posterior half of the process.

The concave capsular surface on either side of the supraoccipital forms the dorsomedial vestibular portion (vestibular recess) of the cavum capsularis (the central hollow cavity of the osseous labyrinth) and extends ventrally to contact the prootic anteriorly and the otooccipital posteriorly. The interior surface of the supraoccipital portion of the cavum capsularis is largely smooth but contains a distinct grooved surface (utricular recess) posterodorsal to the osseous common crus which connects with the paths for the anterior and posterior semicircular canals. On the ventral surface of the supraoccipital, the space between the supraoccipital portions of the cavum capsularis forms a distinct hourglass shape. The path for the anterior semicircular canal is visible on the lateral articulation surface with the prootic and the path for the posterior semicircular canal on the articulation surface with the otooccipital. The path for the anterior semicircular canal is most easily visible from an anterolateral view and the path for the posterior semicircular canal from a posterolateral view.

The endolymphatic foramen is visible on the medial (external) surface of the supraoccipital portion of the cavum capsularis (Fig 32C) and pierces downwards through the bone into the vestibular recess. On the lateral (internal) surface of the vestibular recess, the endolymphatic foramen opening is visible anterior to the osseous common crus. In MVZ 75918 the supraoccipital is almost entirely fused to the underlying otooccipital and prootic elements.

### Basioccipital

**Fig 33:**
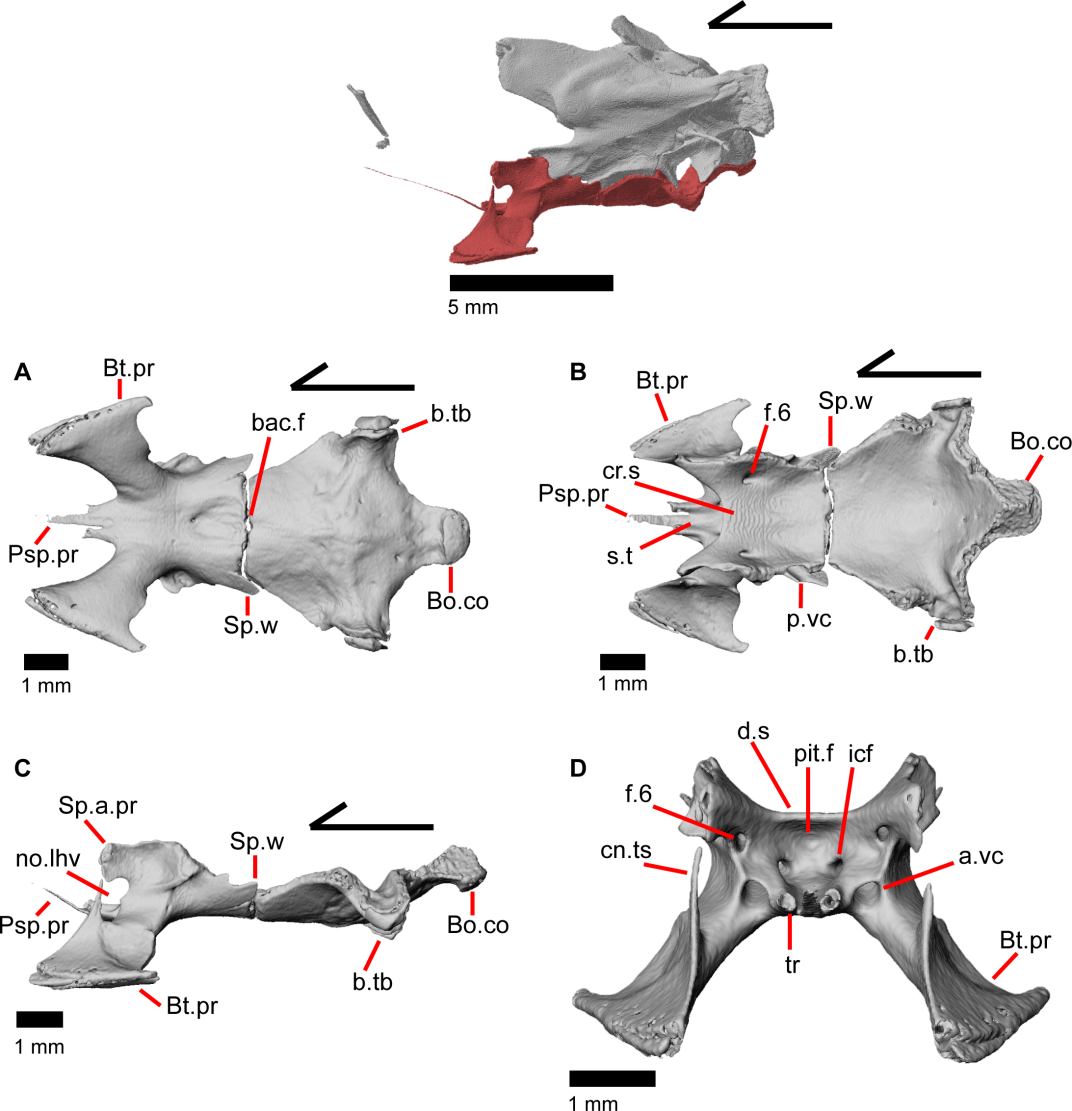
Sphenoid and basioccipital of *Elgaria panamintina* MVZ 191076. (A) Sphenoid and basioccipital from a ventral view. (B) Sphenoid and basioccipital from a dorsal view. (C) Sphenoid and basioccipital in lateral view. (D) Sphenoid in anterior view.

The basioccipital forms the posteroventral portion of the braincase. The element contacts the sphenoid anteriorly and laterally (Fig 33A–33C), the otooccipital laterally and posteriorly, and the prootic anterolaterally. The basioccipital is widest midway along its length and narrows anteriorly closer to the contact with the sphenoid. The basioccipital tapers posteriorly towards the occipital condyle which contacts the atlas vertebra. The basioccipital contributes the medial, lower third of the occipital condyle. The bone is excluded from contributing to the lower margin of the foramen magnum by the otooccipital portion of occipital condyle. The basioccipital portion of the occipital condyle is rounded ventrally and triangular shaped.

The dorsal surface of the basioccipital contributes to the posterior floor of the cranial cavity, and is relatively smooth and dorsally concave. The ventral surface is convex with small ridges that extend laterally towards the basal tubercles. Basal tubercles point ventrolateral, extending ventrally below the main body of the element and make up the ventral border of the lateral aperture for the recessus scali tympani (Fig 33B and 33C). The basal tubercles also form the ventral extension of the crista tuberalis. There is a variable amount of calcified cartilage just lateral to the basal tubercles, which may represent sesamoid bones [58]. In MVZ 75918, the basioccipital is largely fused to the elements of the braincase that it contacts.

### Sphenoid

The sphenoid is an irregularly shaped element that constitutes the anteroventral portion of the braincase. The sphenoid contacts the basioccipital posteriorly (Fig 33C). The two bones have a transversely straight contact medially and two sphenoid wings of the sphenoid extend posterolaterally to contact the basioccipital. The sphenoid wings extend posteriorly for a short while along the lateral edges of the basioccipital-prootic suture. The sphenoid contacts the prootic dorsolaterally. The facet for prootic articulation is a relatively flat surface, which curves upwards anteriorly. On the lateral side of the sphenoid, there is a triangular facet where the prootic articulates. There is a shallow depression on the ventral surface of the sphenoid just anterior to basicranial fenestra (Fig 33A). The basispterygoid process of the sphenoid is a paired anteroventral process that curves laterally to meet the pterygoid, forming the synovial palatobasal articulation. The parasphenoid process extends anterodorsally from the anterior face of the bone. There are two trabeculae, one on either side of parasphenoid process, that form cylindrical canals and contain foramina which appear to open somewhere near the pituitary fossa (Fig 33D). Medially, the dorsum sella is a pronounced ridge which also forms the back wall of the pituitary fossa and connects with the crista sellaris which forms the anteriormost surface of the braincase floor. The sella turcica constitutes the space between the trabeculae, extending to the back of the pituitary fossa [24]. There is a carotid foramen on either side of the pituitary fossa. These foramina empty into and merge with the vidian canals. The opening of the carotid canal is best viewed from an anterolateral angle.

The anterior opening of the vidian canal is large and is located lateral and posterior to the trabecula. Posterior sutural processes form the ventral margin of the posterior exit of the vidian canal; the prootic forms the medial and posteriormost margin of the posterior opening of the vidian canal. The abducens foramen is medial to the ventral margin of the alar process of the sphenoid, and opens posteriorly on the dorsal surface of the sphenoid.

There is a robust connection between the basipteryogid process and the alar process of the sphenoid in MVZ 75918 with ossified connective tissue forming the vidian bridge. This results in a large foramen that encloses the lateral head vein (internal jugular vein of Criley [24]). However, there is incomplete connection on the left side of MVZ 191076, thus forming only a notch for the lateral head vein. A small basicranial fenestra is present in both specimens.

### Prootic

**Fig 34:**
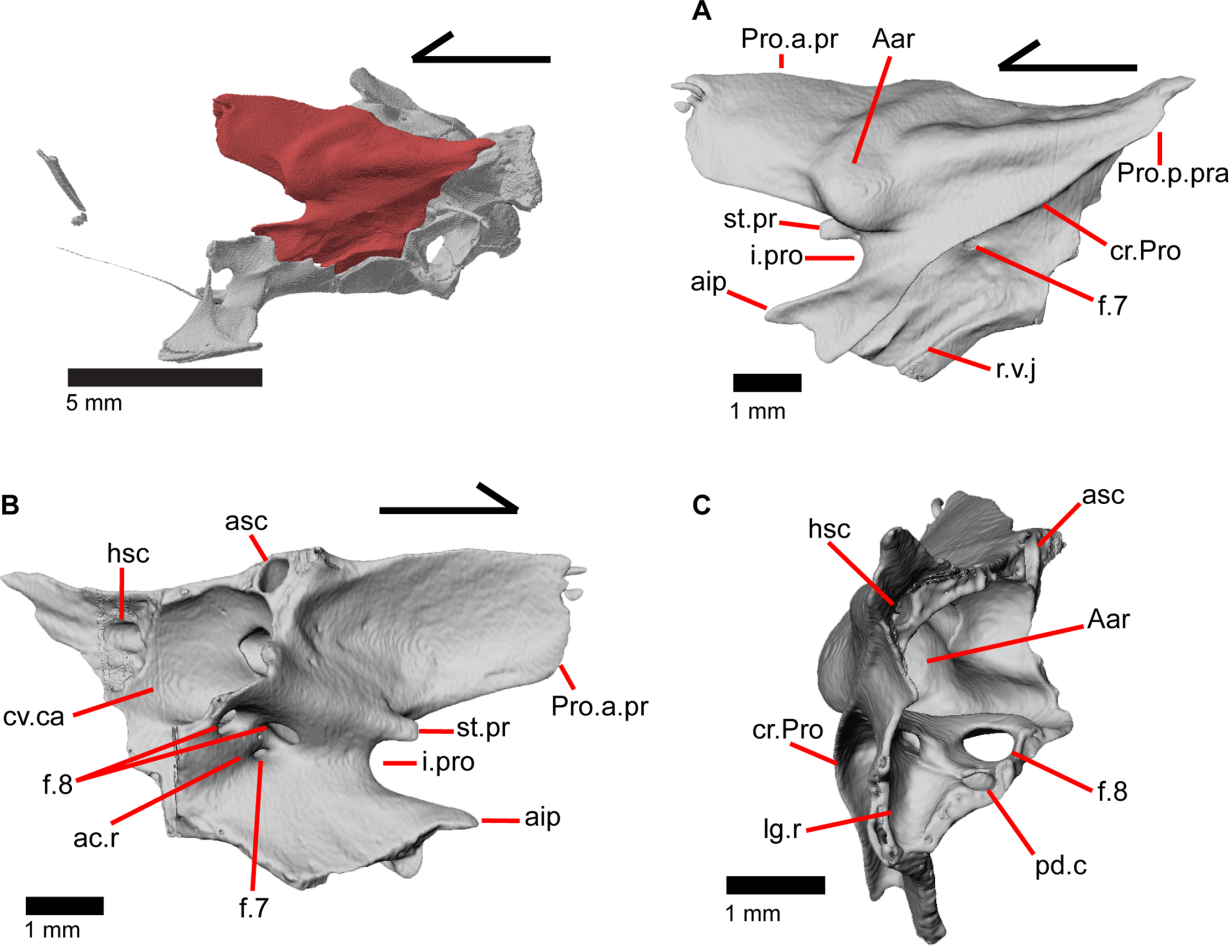
Left prootic of *Elgaria panamintina* MVZ 191076. (A) Lateral view. (B) Medial view. (C) Posterior view.

The prootic contributes the anterolateral portion of the ossified braincase and the anterior portion of the cavum capsularis. It contacts the supraoccipital dorsally, the otooccipital posteriorly, the basioccipital ventrally, and the sphenoid anteroventrally. The prootic possesses mediolaterally compressed alar processes (Fig 34A; dorsal anterior wing of McDowell and Bogert [57]) that extend anteriorly to contact the dorsal portion of the epipterygoid. Contact between the alar process and eipipterygoid may also be observed in dry skeletal specimens of *Elgaria multicarinata* (Fig 31B). The prootic has an anterior inferior process that extends anteriorly approximately to the mid-point of the alar process of the prootic and which articulates dorsally with the sphenoid. The incisura prootica (trigeminal notch of McDowell & Bogert [57]) is formed by the ventral margin of the alar process, the dorsal margin of the anterior inferior process, and an anterior portion of the anterior ampullar recess. The incisura prootica is a distinct notch through which the trigeminal nerves pass [46]. A supratrigeminal process was previously reported to occur and to divide the incisura prootica in *Gerrhonotus* and some *Elgaria* [43]. This supratrigeminal process is small and not visible in lateral view on MVZ 75918, and is only visible in lateral view on the left prootic on MVZ 191076.

The external surface of the ampullar recess is located just posterior to the alar process and has a convex lateral face. The crista prootica is a strongly developed crest that runs from the posteroventral border of the anterior inferior process to the posterior process of the prootic, which contacts the paroccipital process of the otooccipital. The lateral surface of the sphenoid possesses a raised area that continues the crista prootica anteriorly. The facial foramen is most easily seen from a ventrolateral view and lies just under the crista prootica at about the midpoint along the crest. A portion of the ventral edge of the prootic where it contacts the sphenoid forms part of the border of the posterior opening for the vidian canal. From this posterior opening, there is a ridge that begins on the sphenoid and continues onto the prootic and which forms the ventral border of the recessus vena jugularis. The recessus vena jugularis is a groove that extends from the posterior opening of the vidian canal to just ventral and posterior to the facial foramen.

The posteromedial surface of the prootic forms part of the medial border of the cavum capsularis. Ventral to this, the prootic contains a small pocket known as the acoustic recess (Fig 34B) into which the facial foramen (VII) opens medially, and two acoustic foramina open and carry branches of the vesibulocochlear nerve (VIII) [46]. The facial foramen is noticeably smaller than the two acoustic foramina and is located more ventrally. The posterior acoustic foramen opens posteriorly into the cavum capsularis. The anterior acoustic foramen opens into the anterior ampullar recess which is extensive anteriorly. The cavum capsularis is divided into a dorsal vestibular and ventral lagenar cavity (lagenar recess). On the prootic of *Ctenosaura pectinata*, these two regions are separated by a lagenar crest [46]. In many scleroglossans, however, these regions are further divided by a groove, bounded dorsally by the lagenar crest, which carries the perilymphatic duct [45]. In *Elgaria panamintina* the lagenar crest is fused ventrally and creates a canal through which the perilyphatic duct presumably passes (Fig 34C). A small foramen enters the canal and opens medially within the acoustic recess above the facial foramen (Fig 34B). The prootic possesses two openings for semicircular canals (Fig 34C), including a horizontal semicircular canal opening on the posterior process of the prootic ventral to the otooccipital facet. The opening for the anterior semicircular canal is located where the prootic articulates with the supraoccipital. On the posterior edge of the bone, the prootic forms the anterior border of the fenestra ovalis. In MVZ 75918, many of the brain case elements are fused together and there are no obvious sutures between the prootic-otooccipital and the prootic-sphenoid.

### Otooccipital

**Fig 35:**
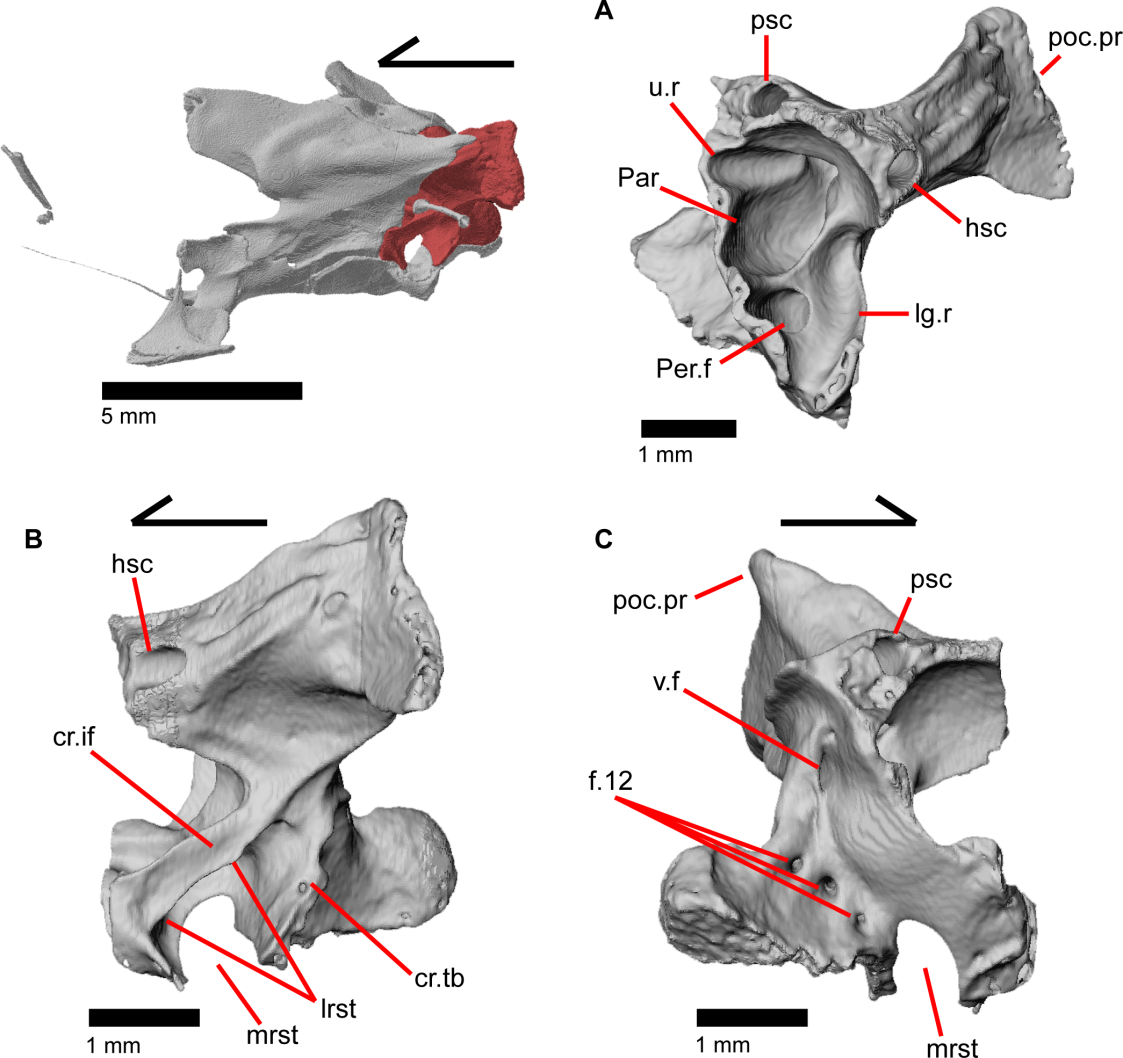
Left otooccipital of *Elgaria panamintina* MVZ 191076. (A) Anterior view. (B) Lateral view. (C) Medial view.

The otooccipital forms the posterodorsal portion of the braincase and the posterior portion of the cavum capsularis. It contacts the supraoccipital dorsally, the basioccipital ventrally, and the prootic anterolaterally. Along the contact with the supraoccipital there is an opening for the posterior semicircular canal. Along the contact with the prootic, there is an opening for the horizontal semicircular canal (Fig 35A). The spinal cord passes through the foramen magnum which is a large opening bordered by the supraoccipital and paired otooccipital. The otooccipitals form the dorsolateral portions of the occipital condyle. The occipital condyle does not appear to be constricted in dorsal or ventral view, however, there is a slight constriction in lateral view on the basioccipital portion of the occipital condyle [3]. On the lateral surface of the otooccipital, just posterior and ventral to the fenestra ovalis, is a low ridge of bone called the crista interfenestralis (Fig 35B) [59]. The crista interfenestralis becomes reduced anteriorly at the contact with the prootic and merges with the crista tuberalis posteriorly. The crista tuberalis is a defined crest that extends dorsally to the ventral portion of the paroccipital process and ventrally to the basal tubercle. The paroccipital process of the otooccipital is large and appears compressed in posterolateral view. The paroccipital process projects posterolaterally from the main body of the bone to contact the posterior end of the supratemporal, the cephalic condyle of the quadrate and the postparietal process. The length of the paroccipital process was previously shown to vary ontogenetically in *Elgaria multicarinata* [55].

**Fig 36:**
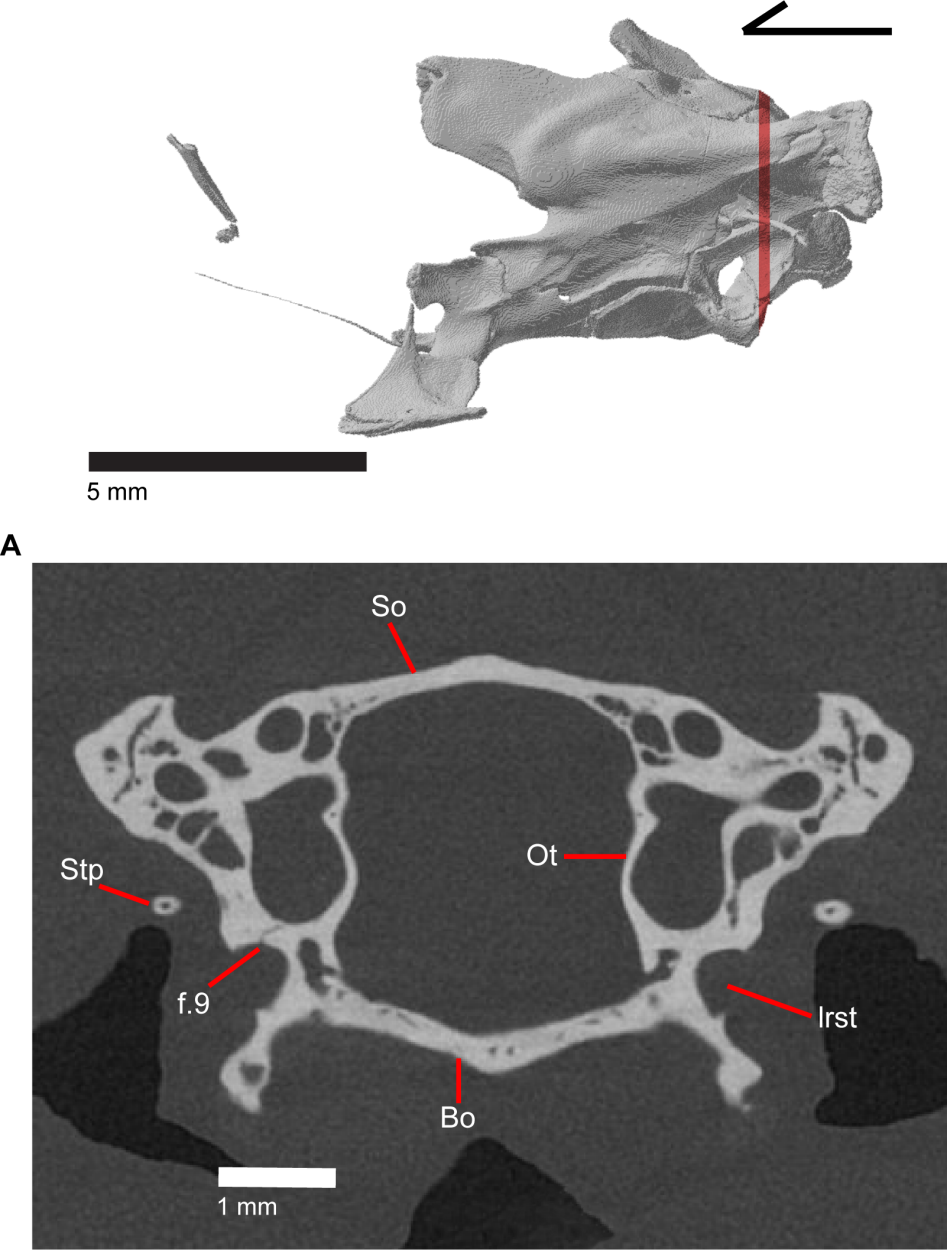
CT coronal slice #1440 of *Elgaria panamintina* MVZ 75918.

The otooccipital forms the posterior border of the fenestra ovalis, a large ovoid fenestra in which the footplate of the stapes sits. The fenestra ovalis is asymmetrically divided among the prootic and otooccipital, with the otooccipital contributing a greater portion of its border than does the prootic. The fenestra ovalis opens medially just above the lagenar recess, which is formed by the ventral articulation between the prootic and otooccipital. The lateral aperture for the recessus scala tympani is a relatively large opening located dorsal to the basal tubercle on the lateral side of the basioccipital. The lateral aperture is bounded dorsally by the crista interfenestralis and crista tuberalis of the otooccipital and ventrally by the basioccipital. The medial aperture of the recessus scala tympani is visible through the lateral aperture and is also bordered by the otooccipital dorsally and basioccipital ventrally. The medial aperature is well-developed and oval shaped but smaller than the lateral aperture. The perilymphatic foramen (fenestra cochlea of Criley [24]) opens from the cavum capsularis (specifically, the dorsal portion of lagenar recess) and empties into the dorsal part of the recessus scala tympani. There is a separate glossopharyngeal foramen sitting under the perilymphatic foramen only in MVZ 75918 (Fig 36A). Dorsal and posterior to the perilymphatic foramen, there is a ridge that separates the lagenar recess from the posterior ampullar recess, which connects to the posterior and horizontal semicircular canals. On the medial surface of the otooccipital portion of the cavum capsularis, there is a distinct groove (utricular recess) dorsal to the posterior ampullar recess. This groove continues anteriorly onto the supraoccipital.

**Fig 37:**
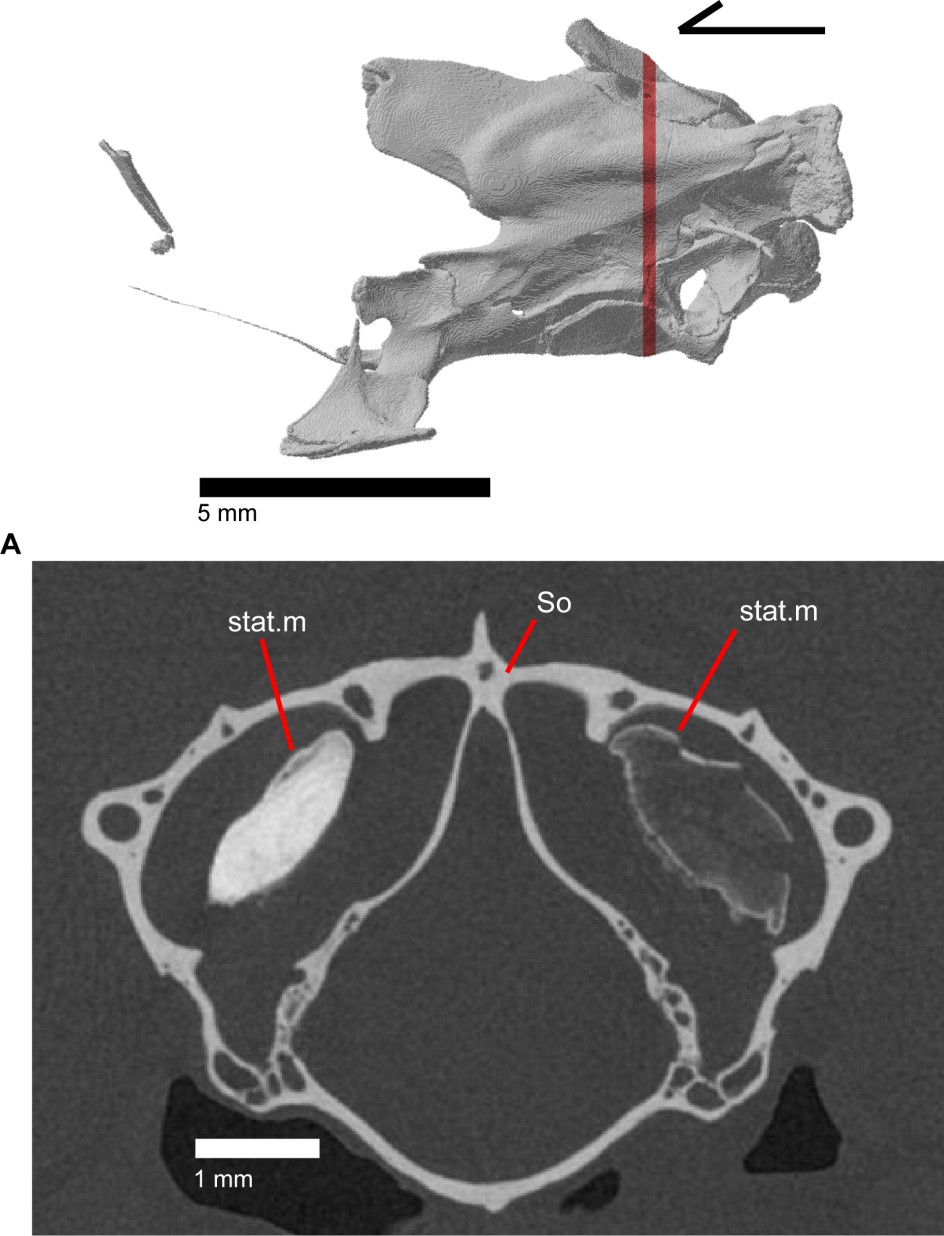
CT coronal slice of *Elgaria panamintina* MVZ 75918.

The external opening for the vagus foramen is located on the posterior surface of the otooccipital and can be found above two external openings for hypoglossal foramina. The vagus foramen is larger than the hypoglossal foramina, and runs horizontal through the medial surface of the otooccipital and opens just inside the foramen magnum (Fig 35C). Two additional external openings for hypoglossal foramina (CN12) are located just posterior to the crista tuberalis. Three internal openings for the hypoglossal foramina can be found on the ventromedial surface of the otooccipital just inside the foramen magnum. Dorsal to the vagus foramen, there is a distinct crest (described by Oelrich [46] but not named) that connects to the posterior edge of the supraoccipital and helps to form the dorsolateral border of the foramen magnum. Just dorsal to this crest, there are one or two foramina. There are large and dense (a high threshold and visible as a bright mass in CT scans) statolithic masses in the vestibules of *Elgaria panamintina* MVZ 75918. They are asymmetric in that the left side is denser while the right side is larger (Fig 37A). The statolithic masses in *Elgaria panamintina* MVZ 191076 are smaller and less dense, making them difficult to observe from the CT data.

Based on fusion of the braincase, *Elgaria panamintina* MVZ 75918 seems to be a skeletally mature adult specimen while the lack of fusion in *Elgaria panamintina* MVZ 191076 may indicate a specimen that is not fully grown [54].

### Stapes

The stapes (columella) is a thin, rod-like bone that is expanded at its ends (Fig 29A and 29B). The two ends are similar in shape, with the medial end being only slightly more expanded than the lateral end. The bone runs posterolaterally with the lateral end positioned posterior to the central column of the quadrate. The stapes is hollow and a canal runs through the bone.

### The hyoid apparatus

**Fig 38:**
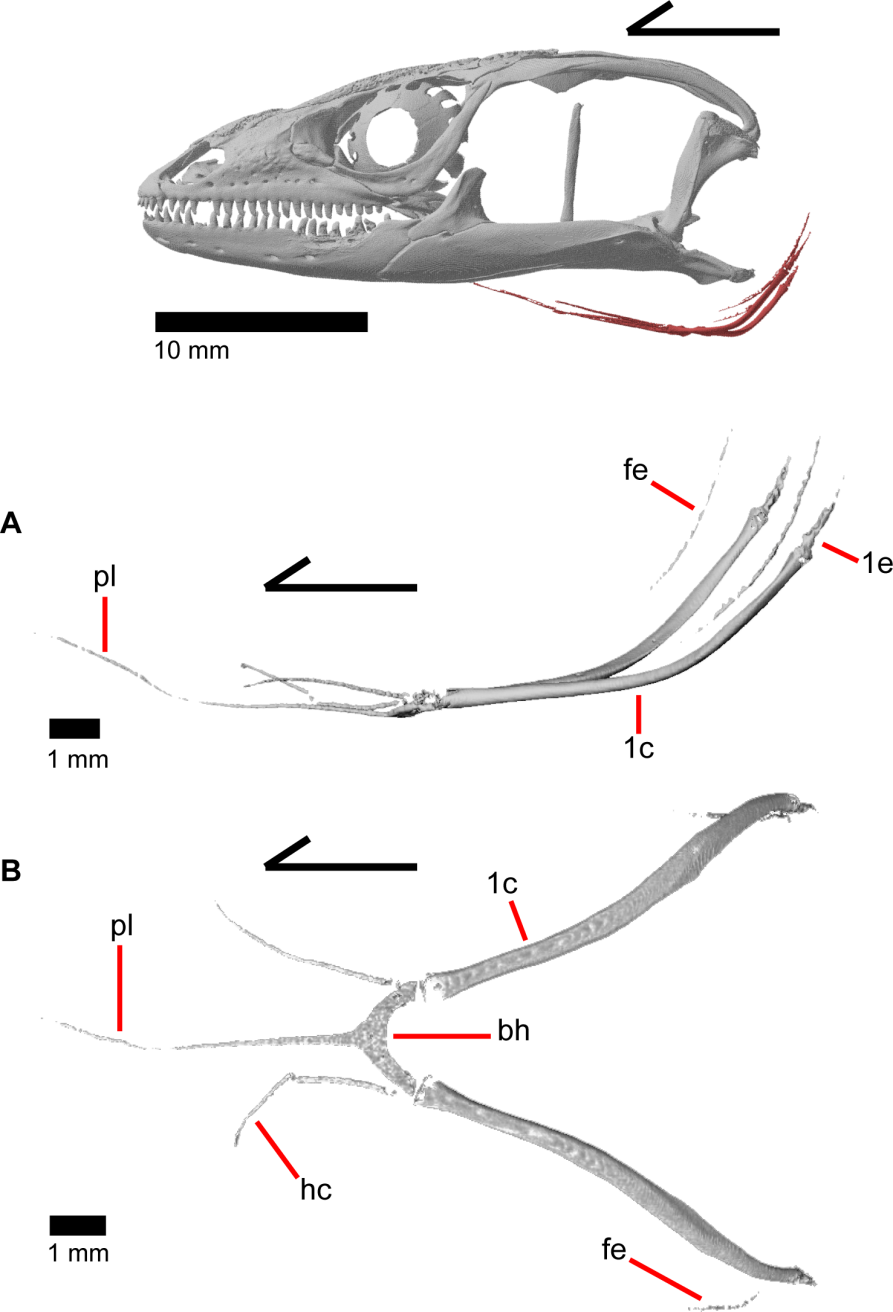
Hyoid apparatus of *Elgaria panamintina* MVZ 75918. (A) Lateral view. (B) Ventral view.

The hyoid apparatus (terminology following Cope [60] and Bell et al. [45]) consists of long, thin ossifications located in the posteroventral region of the skull that are not in contact with other cranial elements (Fig 38A and 38B). The central body, or basihyal, is triradiate and directly ventral to the braincase. It is fused to the anteriorly projecting processus lingualis that extends to a point ventral to the interpterygoid vacuity between the palatal regions of the pterygoids. The lateral ends of the basihyal approach articulation with a pair of lateral hyoid cornua (hypohyal of Cope [60]). The hyoid cornu curves anterolaterally. The hyoid cornu curves laterally to a greater extent in MVZ 75918 than in MVZ 191076, and the left element of MVZ 75918 appears to be slightly fractured (Fig 38B). The hyoid cornu terminates ventral to the articulation between the basipterygoid processes of the sphenoid and the pterygoid. Paired first ceratobranchials approach articulation with the lateral ends of the basihyal. The first ceratobranchial extends posteriolaterally and, with the basihyal, are the thickest element of the hyoid apparatus. The first ceratobranchial increasingly curves dorsally at the posterior end and terminate at a point just posterolateral to the retroarticular process of the articular. In MVZ 191076, the right first ceratobranchial is fractured. The free epibranchial is a posterior element of the hyoid apparatus [45, 61, 62]. The free epibranchial does not contact any other part of the hyoid apparatus, but lies just anterior to the first ceratobranchial and extends more dorsally. The free epibranchial has a curvature that is similar to that of the ceratohyal, however, the element is oriented slightly more in the anteroposterior direction and terminates ventral to the retroarticular process of the articular. Both MVZ 191076 and MVZ 75918 possess additional ossifications on the posterior ends of the ceratohyals that may represent ossification of the first epibranchial.

### Osteoderms

**Fig 39:**
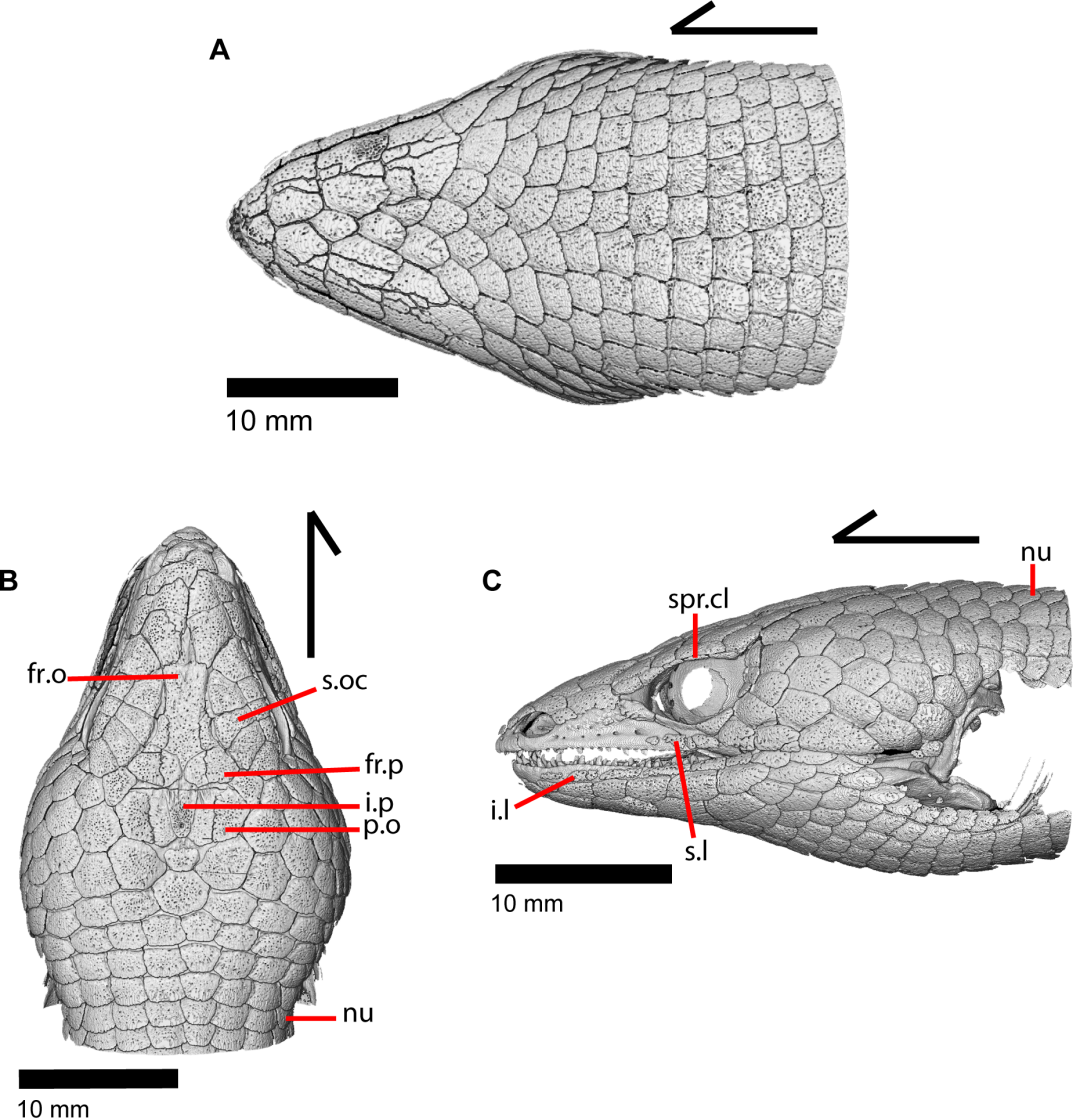
Osteoderms of *Elgaria panamintina* MVZ 75918. (A) Ventral view. (B) Dorsal view. (C) Lateral view.

*Elgaria panamintina*, like other gerrhonotines, possesses well-developed rectangular or trapezoidal-shaped cephalic osteoderms that surround most of the skull [3, 5, 24]. The posteroventral and posterodorsal osteoderms become increasingly square-shaped (Fig 39A and 39B). Like in other anguids, the osteoderms are non-compound and laterally suturing [5, 7, 26]. The pattern and shape of the osteoderms reflects the shape and pattern of some of the overlying scales, but not all. Osteoderms fused to the frontal overlie a large part of the bone. The frontoparietals are excluded from contacting each other by the frontal osteoderm and the smaller interparietal osteoderms. The parietal osteoderms flank the interparietal laterally. Osteoderms are present under the nuchal scales just posterior to the skull, but are not present lateral and dorsolateral to the retroarticular process or lateral to the cephalic condyle (Fig 39C). Additionally, osteoderms are reduced in number (5–6) and less robust along the tooth row of the dentary under the infralabial scales, and are almost completely absent (2–3) and less robust under the supralabial scales along the maxillary tooth row. They are completely absent under the rostral and mental scales, although thin osteoderms are present above the nasal process of the premaxilla. There are four to six long, thin osteoderms above the orbital region under the supracilliary scales, and seven to eight osteoderms beneath the supraocular scales. Two to three osteoderms are present on top of the maxillary facial process, and may in part fuse to the maxilla. Osteoderms frequently fuse to the frontal, parietal, and the nasals, often obscuring part or all of the dorsal surface of those elements from view in dry skeletal material [24].

The dorsal texture of the osteoderms is moderately sculptured and pitted with circular or ovoid pits. Many of the anterodorsal osteoderms do not imbricate and a few do not touch each other, but most of the anteroventral osteoderms contact and some imbricate (especially those that are posterior to the dentary). Both dorsal and ventral postparietal osteoderms imbricate and all have an anterior gliding surface.

Osteoderms in the mandibular and orbital regions are somewhat irregularly shaped, and their arrangement non-uniform. The ventral postparietal osteoderms are regularly shaped and distributed in relatively straight transverse rows that are also straight longitudinally. The osteoderms of *Abronia*, *Barisia*, and *Mesaspis* are more heavily sculptured than those of *Elgaria*, and have a different pitting pattern. Species in the genus *Gerrhonotus* have osteoderms more comparable to *Elgaria*, but may have a different shape and sculpturing pattern, especially on the ventral osteoderms. The osteoderms of *Elgaria panamintina* are comparable to those of other species of *Elgaria*.

### Ontogeny

Both specimens of *Elgaria panamintina* we examined represent adult males, as documented by the collectors of the specimens and suggested by the large size of the specimens. Osteological features also indicate that both specimens represent adult individuals. These features include the relatively small size of the parietal foramen (Fig 18A and 18B) and basicranial fenestra (Fig 33A), medially constricted lateral edges of the parietal that give the parietal table an overall trapezoid-shaped appearance (Figs 1, 18A and 18B), the presence of well-developed and fully ossified osteoderms (Fig 39), and the long extension of the alar process of the prootic (Fig 34A) and the paroccipital process of the otooccipital (Fig 35A) relative to juvenile specimens [55].

Although both specimens appear to represent mature individuals, we hypothesize that MVZ 75918 is older based on some osteological differences between the specimens. The anterior extension of the supratemporal was documented to vary ontogenetically in other species of *Elgaria* [47] and in other lizards [43], wherein the supratemporal extends more anteriorly in older individuals. In MVZ 75918, the supratemporal extends anterior to the posterior tip of the postorbital. In MVZ 191076, the anterior tip of the supratemporal ends just posterior to the posterior tip of the postorbital. MVZ 75918 also possesses increased fusion between elements in the braincase relative to MVZ 191076. Complete fusion of the supraoccipital with the prootic and otooccipital as well as the fusion of the dorsal articulation between the prootic and otooccipital was reported to occur after sexual maturity in *Elgaria coerulea* [54]. In MVZ 75918, the supraoccipital is fused with the prootic and the otooccipital (Fig 31A) and the other elements of the braincase are also fused to each other, suggesting that the specimen represents an individual that had reached sexual maturity. The braincase elements of MVZ 191076 are not fused to each other and sutures between elements are easily distinguished (Figs 29 and 33A–33C), indicating that the individual may not have reached sexual maturity. However, interspecific variation in the timing of complete fusion between braincase elements has been documented in *Xantusia* and could also be the case for species of *Elgaria* [54].

The left jugal of MVZ 191076 lacks a spur. In *Elgaria coerulea*, the jugal spur was reported to begin forming only after adult size was reached [47], but we find that this feature may not be a reliable indicator of life stage for all species of *Elgaria*. Some specimens of juvenile *Elgaria multicarinata* that we examined possess a well-developed jugal spur on both sides, and the presence of this morphology in juvenile *Elgaria* is corroborated by the findings of other researchers [55].

Much work is needed to establish clear understanding of postnatal osteological development in gerrhonotine lizards and in other squamates. Osteological development through ontogeny varies between squamate clades [54], indicating a need for detailed examination of the skeleton throughout ontogeny to better understand patterns of osteological variation in squamates. Documenting variation is necessary for phylogenetic studies using morphology of both modern specimens and fossils [55].

### Comparison of the skulls of *Elgaria panamintina* and *Elgaria multicarinata*

Here, we discuss interspecific variation found between *Elgaria panamintina* and *Elgaria multicarinata*. We describe the osteological differences that we found between these species, providing a benchmark for the osteology of *Elgaria panamintina* using the well-known species *Elgaria multicarinata*. Because there is currently only one dry skeletal specimen of *Elgaria panamintina*, the CT data from the two CT scanned alcohol preserved specimens of *Elgaria panamintina* serve as important osteological exemplars for the species. In the future, an increase in osteological data will shed more light on the types of variation present in this species and build upon the variation discussed in this paper.

The species *Elgaria multicarinata* was chosen for comparison with *Elgaria panamintina* due to the relative abundance of specimens of *Elgaria multicarinata*. In addition, aspects of the osteology of *Elgaria multicarinata* were previously examined or included in many larger analyses [3, 7, 13, 21, 24, 26, 27, 32, 36, 43, 44, 55, 63, 64]. As previously stated, the results of phylogenetic analyses of molecular data recovered *Elgaria panamintina* and *Elgaria multicarinata* as sister species ([6], see [42] for hypothesized mitochondrial introgression in *Elgaria*).

Interspecific variation between *Elgaria panamintina* and *Elgaria multicarinata* is organized by cranial element. For cranial elements that are excluded, no interspecific variation was discovered between the two species.

#### Premaxilla

Specimens of *Elgaria panamintina* lack a foramen on the anterior surface of the alveolar plate of the premaxilla. In all specimens of *Elgaria multicarinata* this foramen is present when not obscured by fused osteoderms (Fig 11C). Although it was not previously reported to occur within *Elgaria*, an ossified bridge extending laterally from the nasal process to exclude the foramen for ophthalmic branch of CN5 (medial ethmoidal foramen of Good [3]) from the naris occurs in at least one specimen of *Elgaria multicarinata* [13]. Although we were not able to examine the specimen, a published figure documented the presence of the bridge on one side of the premaxilla in *Elgaria multicarinata* TMM M-8993 [13]. In a publication currently in preparation, we find that an ossified bridge occurs in other species of *Elgaria* as well. Although both specimens of *Elgaria panamintina* possess relatively straight nasal processes, there is considerable intraspecific variation in the morphology of that process [3]. *Elgaria multicarinata* TNHC 35666 has a nasal process that is greatly broadened at the mid-point and *Elgaria multicarinata* TNHC 4478 has a thin nasal process that is constricted at the base. An oddity was found in a specimen of *Elgaria multicarinata* (TMM M-9004), which only possessed eight tooth positions on the premaxilla as opposed to the typical nine tooth positions found in all other specimens (Fig 11D).

#### Palatine

In both specimens of *Elgaria panamintina*, the cristae cranii of the frontal do not extend ventrally below the dorsal tip of the palatine [2] and the palatine lacks a dorsally expanded apex (Fig 17A and 17B) seen in most specimens of *Elgaria multicarinata*. However, in all specimens of *Elgaria multicarinata* besides TNHC 4478, the cristae cranii project below the apex of the palatine and the bones contact in some dry skeletal specimens (TMM M-8974, TMM M-9005, TMM M-9007). A possible explanation for why the cristae cranii do not project below the palatine in *Elgaria multicarinata* TNHC 4478 (Fig 17C) may be that the cristae cranii do not seem to be fully ossified, because there is free-floating bone at the ventral ends. The inconsistency between frontal-palatine contact in dry skeletal specimens and lack of contact in CT specimens may suggest that contact may be a result of shrinkage resulting from skull preparation [43]. In specimens of *Elgaria panamintina* and *Elgaria multicarinata*, there is a tendency for the lateral maxillary process of the palatine to be thinner and to have a tip that is pointed posterolaterally and in contact with the jugal [3]. Other species of *Elgaria* tend to have more blunt processes and lack contact with the jugal. There is a possibility that the morphology of the lateral maxillary process may change through ontogeny [55].

#### Frontal

Foramina or notches can be found on the anterolateral processes on the frontals (Fig 16B and 16C) of specimens of *Elgaria multicarinata* as well as on the frontal of *Elgaria panamintina* MVZ 75918. However, there are no foramina or notches on the frontal of *Elgaria panamintina* MVZ 191076.

#### Jugal

The jugals of specimens of *Elgaria multicarinata*, particularly those of TNHC 35666 and TNHC 4478, are thicker near the inflection point than are the jugals of *Elgaria panamintina* (Fig 15A, 15C and 15D). This variable morphology may be a result of ontogenetic change in the shape of the jugal as previously shown in *Elgaria multicarinata* [55].

#### Septomaxilla

Although having a dorsally curved posterior process of the septomaxilla was previously reported to be characteristic of *Elgaria* [3], the CT-scanned specimens *Elgaria multicarinata* TNHC 35666 and *Elgaria multicarinata* TNHC 4478 have septomaxillae with a posterior process that is relatively straight (Fig 27C). However, both specimens of *Elgaria panamintina* possess some degree of dorsal curvature on the posterior process of the septomaxilla (Fig 27B). It proved difficult to analyze this morphology because we were only able to find one septomaxilla on a disarticulated dry skull. All other septomaxillae on dry skeletal specimens were examined through the naris and at a slight angle which may cause a skewed perspective of the morphology of the posterior process.

#### Lacrimal

In *Elgaria multicarinata* TNHC 35666 and *Elgaria multicarinata* TNHC 4478, the lacrimal articulates with the maxilla at a noticeably more inclined angle than that in both specimens of *Elgaria panamintina* (Fig 15A, 15C, and 15D). However, in dry skeletal specimens of *Elgaria multicarinata*, there is much more variation in the angle at which the lacrimal lies on the maxilla, potentially because of variation in the height of the anterior portion of the orbital process of the maxilla, which may vary with ontogeny [55]. Although both specimens of *Elgaria panamintina* as well as *Elgaria multicarinata* TNHC 35666 have a relatively short posterior process of the lacrimal, *Elgaria multicarinata* TNHC 4478 and some dry skeletal specimens of *Elgaria multicarinata* (TMM M-8975, TMM M-9007) have an elongated posterior process.

#### Epipterygoid

In both specimens of *Elgaria panamintina*, the epipterygoid does not contact the parietal. With the exception of *Elgaria multicarinata* TNHC 4478 and TMM M-9004, all other examined specimens of *Elgaria multicarinata* with an articulated skull show contact between the epipterygoid, the alar process of the prootic, and the epipterygoid process of the parietal (Fig 31B and 31C). The parietal and alar processes contact each other medially, and the epipterygoid contacts both bones laterally. This variation does not seem to be a result of the preservation of the skull because contact is observed in both CT and dry skeletal specimens.

#### Postorbital

In specimens of *Elgaria panamintina*, the postorbital possesses a large medial expansion just posterior to the anterior end. That expansion is not found to this extent in specimens of *Elgaria multicarinata* (Fig 23B) except for in TMM 8987 (Fig 23C). In addition, the postorbital of *Elgaria panamintina* contributes to more of the length of the border of the supratemporal fenestra than does the postorbital of *Elgaria multicarinata*.

#### Quadrate

In disarticulated dry skeletal specimens of *Elgaria multicarinata*, there is no preserved calcified cartilage forming a squamosal socket. This contrasts with articulated dry skeletal specimens as well as CT data, both of which have preserved and visible calcified cartilage.

#### Coronoid

In both specimens of *Elgaria panamintina*, the posterior coronoid process extends just ventral to the adductor fossa on the fused articular and surangular. There is intraspecific variation in this morphology within *Elgaria multicarinata*; the posterior process of the coronoid extends below the adductor fossa in *Elgaria multicarinata* TMM M-9004 (Fig 6D) and on the right side in *Elgaria multicarinata* TMM M-9007. In all other specimens of *Elgaria multicarinata* the posterior coronoid process lies anterior or anteroventral to the adductor fossa.

#### Orbitosphenoid

The anterior end of the orbitosphenoid is broadened in both *Elgaria panamintina* and in *Elgaria multicarinata* (Fig 30A and 30C), but in specimens of *Elgaria panamintina*, a small notch gives the anterior portion of the bone a slightly bifurcated appearance. The discrepancies in shape may be related to differences in body size or a result of individual variation [53].

## Discussion

Our study provides the first description and illustrations of the skull and hyoid apparatus of *Elgaria panamintina*, and is the first detailed description of the skull for any gerrhonotine lizard species. This description serves as the most comprehensive reference for the cranial morphology of the genus *Elgaria*. Additionally, our description and illustrations are detailed enough to be scored for phylogenetic analysis. We found significant intraspecific variation in *Elgaria panamintina* with a sample size of n = 2, demonstrating the importance of thoroughly examining intraspecific, interspecific, and ontogenetic variation in any morphological study or phylogenetic analysis of morphological data. We discovered novel morphology of *Elgaria panamintina* with respect to other *Elgaria*, including morphology that directly contradicts previously reported diagnostic characters of *Elgaria*. For example, we found that the posterior extent of the apex of the facial process of the maxilla of *Elgaria panamintina* was anterior to the midline of the maxilla [2], and MVZ 75918 lacked pterygoid teeth and instead possessed a pitted texture on the ventral surface of the palatal plate [3]. We also found morphology in *Elgaria multicarinata* that contradicts diagnostic characters of *Elgaria*, such as a straight instead of a curved projection of the posterior process of the septomaxilla [3].

We did not detect many differences between the skulls of *Elgaria panamintina* and *Elgaria multicarinata*. Consequently, we recommend that the identifications of fossil specimens previously referred to *Elgaria multicarinata* be reevaluated. It is difficult to distinguish isolated cranial elements of *Elgaria panamintina* from *Elgaria multicarinata*, and a complete or almost complete skull is probably necessary to confidently do so. Even then, consistent differences are few and subtle. A short posterior process of the lacrimal is present in both *Elgaria panamintina* specimens examined and only one of the examined skeletal and CT-scanned *Elgaria multicarinata* specimens. Both *Elgaria panamintina* specimens lack an anterior premaxillary foramen, which is present in all *Elgaria multicarianta* examined. The anterior portion of the postorbital is wider in *Elgaria panamintina* than in *Elgaria multicarinata*, and the entire postorbital contributes to more of the length of the supratemporal fenestra in *Elgaria panamintina*. Although the bifurcate appearance of the orbitosphenoid of *Elgaria panamintina* distinguishes it from that of *Elgaria multicarinata*, the shape and ossification of the orbitosphenoid varies ontogenetically and between individuals [53] and requires further investigation before it is used in a diagnostic or phylogenetic context. Of the lacrimal, premaxilla, postorbital, and orbitosphenoid, only the premaxilla is commonly encountered in museum collections and in the fossil record. Our results support the conclusions of studies that recommended caution in referring fossils to extant species [65].

A vast amount of work remains investigating the cranial osteology of *Elgaria panamintina* and of all gerrhonotine lizards. Researchers must skeletonize *Elgaria panamintina* and multiple specimens of other gerrhonotine species, as well as continue to CT scan additional specimens. Collections of skeletal specimens and CT data sets with a sample size greater than n = 1 are necessary for documenting patterns of morphological variation [66] and thus are critical for all morphological research. In our future work, we will document intraspecific variation with a larger sample of gerrhonotine taxa, allowing for updated phylogenetic analyses of gerrhonotine relationships using morphology and for an improved understanding of the fossil record and biogeographic history of Gerrhonotinae.

### Skeletal specimens of *Elgaria multicarinata* examined and locality data

TMM 8987, San Bernardino County CA; TMM 8988, Santa Barbara County CA; TMM M-8974, Altadena, Los Angeles County CA; TMM M-8975, Riverside County CA; TMM M-8993; TMM M-9004, Alameda County CA; TMM M-9005, Riverside County CA; TMM M-9007, Riverside County CA, TMM M-8578, Riverside County CA.
